# Design, synthesis, molecular docking and biological evaluation of new carbazole derivatives as anticancer, and antioxidant agents

**DOI:** 10.1186/s13065-023-00961-y

**Published:** 2023-06-16

**Authors:** İrfan Çapan, Mohammed Hawash, Nidal Jaradat, Yusuf Sert, Refik Servi, İrfan Koca

**Affiliations:** 1 Department of Material and Material Processing Technologies, Gazi University, Technical Sciences Vocational College, 06560 Ankara, Turkey; 2 Department of Pharmacy, Faculty of Medicine and Health Sciences, An-Najah National University, 00970 Nablus, Palestine; 3 Yozgat Bozok University, Sorgun Vocational School, Yozgat, Turkey; 4 Department of Anatomy, Faculty of Medicine, Firat University, Elazig, Turkey; 5 Department of Chemistry, Faculty of Art & Sciences, Yozgat Bozok University, Yozgat, Turkey

**Keywords:** Carbazole, Anticancer, Antifibrotic, LX-2, Antioxidant, Doxorubicin, Molecular docking

## Abstract

**Background:**

The carbazole skeleton is an important structural motif occurring naturally or synthesized chemically and has antihistaminic, antioxidant, antitumor, antimicrobial, and anti-inflammatory activities.

**Objectives:**

This study aimed to design and synthesize a novel series of carbazole derivatives and evaluate their antiproliferative and antioxidant activities.

**Methods:**

The synthesized compounds were characterized utilizing HRMS, ^1^H-, and ^13^C_APT_-NMR, and assessed for their anticancer, antifibrotic, and antioxidant effects utilizing reference biomedical procedures. In addition, the AutoDock Vina application was used to perform in-silico docking computations.

**Results:**

A series of carbazole derivatives were synthesized and characterized in the current study. Compounds **10** and **11** were found to have a stronger antiproliferative effect than compounds **2**–**5** against HepG2, HeLa, and MCF7 cancer cell lines with IC_50_ values of 7.68, 10.09, and 6.44 µM, respectively. Moreover, compound 9 showed potent antiproliferative activity against HeLa cancer cell lines with an IC_50_ value of 7.59 µM. However, except for compound **5**, all of the synthesized compounds showed moderate antiproliferative activities against CaCo-2 with IC_50_ values in the range of 43.7–187.23 µM. All of these values were compared with the positive control anticancer drug 5-Fluorouracil (5-FU). In addition, compound **9** showed the most potent anti-fibrotic compound, and the cellular viability of LX-2 was found 57.96% at 1 µM concentration in comparison with the positive control 5-FU. Moreover, 4 and 9 compounds showed potent antioxidant activities with IC_50_ values of 1.05 ± 0.77 and 5.15 ± 1.01 µM, respectively.

**Conclusion:**

Most of the synthesized carbazole derivatives showed promising antiproliferative, antioxidant, and antifibrotic biological effects, and further in-vivo investigations are needed to approve or disapprove these results.

**Supplementary Information:**

The online version contains supplementary material available at 10.1186/s13065-023-00961-y.

## Background

**Fig. 1:**
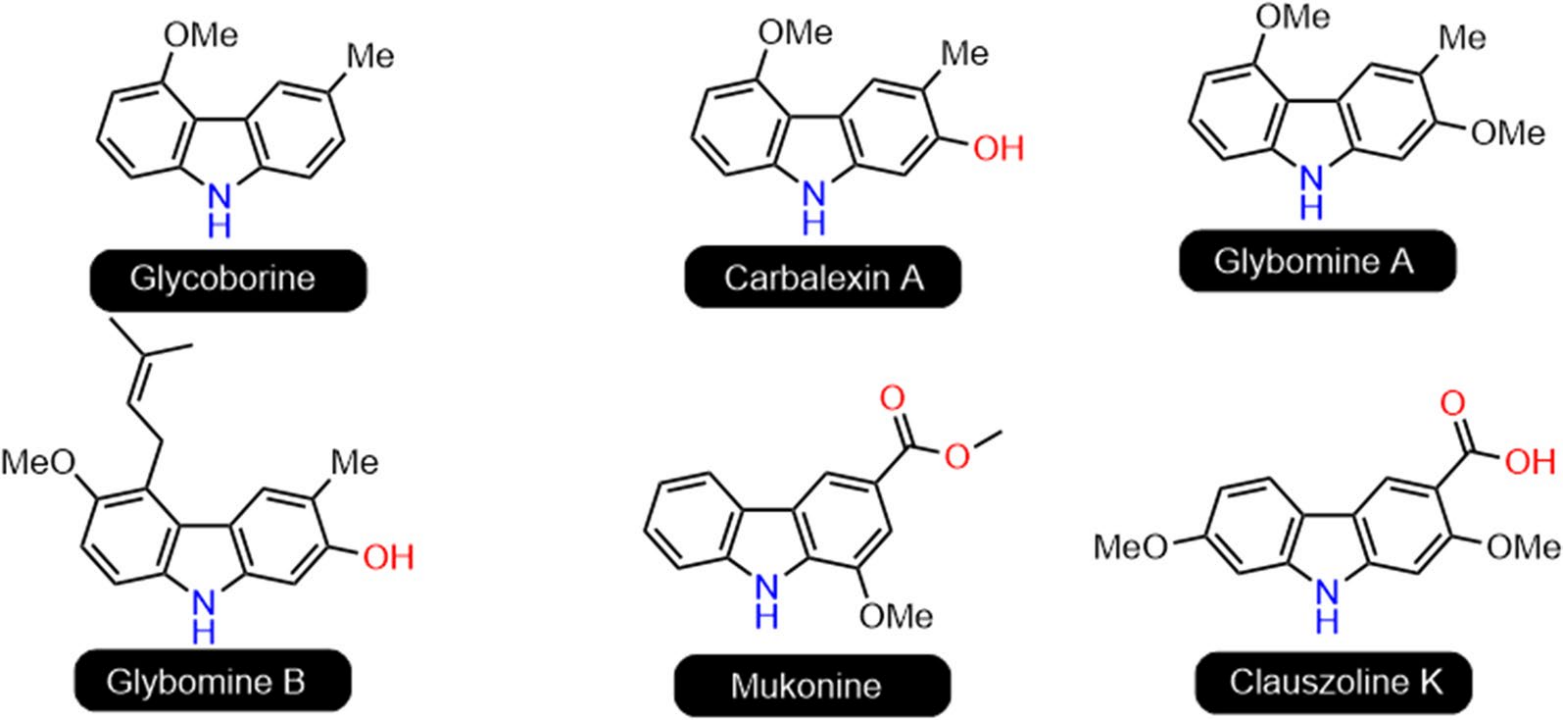
The structures of some carbazole alkaloids with anticancer activities

Carbazole, a tricyclic structure consisting of two six-membered benzene rings fused on either side of the pyrrole ring, is a natural product skeleton abundant in the leaves, fruits, roots and bark of the *Rutaceae* family [1]. Several previous studies have focused on developing anti-cancer agents that containing carbazole moiety as shown in Fig. 1 [2].

Carbazole is a privileged structure because it finds application in many fields. Due to its fluorescent properties, the carbazole skeleton is a structural element of many compounds used for the production of electroluminescent materials, dyes, or polymers [3, 4]. Due to its wide range of pharmacological effects, it is also a useful model that scientists often use to design novel drugs. Carbazole ring-containing molecules display various bioactivities, such as antitumor, neuroprotective, antimicrobial, antihistaminic, antioxidant, anti-inflammatory, and anticoagulant [1, 5–8]. Carbazole is found in some anti-cancer drugs, such as ellipticine, which is a naturally occurring alkaloid that is very effective against a wide range of cancer cell lines. Ellipticine works by inhibiting DNA synthesis and inducing apoptosis in cancer cells. Another example is carbazole-based compounds such as N-methyl carbazole-3-carboxamide, which have shown potent anti-cancer activity against a variety of cancer cell lines. These compounds work by inhibiting the activity of the proteasome, a protein complex that plays a critical role in the regulation of the cell cycle and apoptosis. In addition to these examples, several other anti-cancer agents contain the carbazole moiety, such as carbazomycin A, carbazomycin B, and carbazostatin A, which have shown promising anti-cancer activity in preclinical studies. These compounds work by targeting specific pathways involved in the growth and proliferation of cancer cells, such as the MAPK and AKT signaling pathways [8, 9].

The chemistry and biology of these molecules have received considerable attention due to their use in the synthesis of DNA linkers because of their desired electronic structure and large-β-conjugate surfaces. Furthermore, different functional groups can be attached to the rigid carbazole ring to improve its pharmacological properties [10–12]. The ligands containing the carbazole moiety bind to the minor groove of A/T-rich sequences and interact by intercalation as they contain a flat chromophore [13, 14].

Among the anticancer candidate molecules developed in recent years, there are many carbazole derivatives. These compounds have been designed as hybrids or skeletal functional group derivatizations of carbazole and other anticancer pharmacophores in a single molecule to modulate multiple drug targets for cancer simultaneously. In these ways, carbazole hybrids have the potential to circumvent the serious side effects and drug resistance associated with a single drug molecule [15, 16]. Moreover, some carbazole derivatives have shown potential antiproliferative activity against cancer cell lines by various mechanisms to arrest the cell cycle and induce apoptosis [17]. For example, alectinib, clausnamin A, carvedilol, carprofen, celiptium, LCY-2-CHO, datelliptium, ellipticine, ollivacine, and carazostatin are carbazole-based anticancer agents approved for cancer treatment [18, 19].

The abnormal wound healing response is known as liver fibrosis, which is a result of various chronic liver injuries and is characterized by an excessive buildup of diffuse extracellular matrix (ECM) and abnormal connective tissue hyperplasia. After that, it may develop into liver cirrhosis, liver failure, or liver cancer [20–23]. Although many anti-fibrotic candidate drugs have shown good results in experimental animal models, their anti-fibrotic effects in clinical trials are still limited [24].

Oxidative stress is an important factor in liver damage and liver fibrosis, which produces excess reactive oxygen species and active free radicals in the liver. This makes the antioxidant function weaker and increases the number of active free radicals in the hepatocytes. This causes the hepatocyte membrane to break down and excrete less [25, 26]. Natural or synthetic compounds with antioxidant properties can prevent the oxidation of other molecules, even at low concentrations [27–29]. High levels of free radicals and reactive oxygen species (ROS) play an important role in the emergence of various diseases such as carcinogenesis, drug-induced toxicity, inflammation, atherogenesis, and aging in aerobic organisms [30]. Carbazole-based heterocyclic systems demonstrate strong antioxidant activity on a range of reactive oxygen species [31, 32]. Carazostatin, which is a carbazole derivative, exhibits strong inhibitory activity against free radical-induced lipid peroxidation and shows stronger antioxidant activity in liposomal membranes than α-tocopherol [33]. Therefore, carbazole derivatives are quite valuable molecules for antioxidant studies due to their ability to donate hydrogen or electrons to acceptors like 2,2-diphenyl-1-picrylhydrazyl (DPPH) and to reduce the production of free radicals [34].

In our previous studies on the subject, carbazole-containing molecules were synthesized from the nucleophilic addition-elimination reactions of the corresponding benzoyl chloride and 2-(9* H*-carbazol-9-yl) acetohydrazide to obtain new biologically active compounds [35]. For that, the current investigation aims to assess the anticancer, antifibrotic, and antioxidant activities of new carbazole derivatives synthesized with electron donors, such as aromatic and heterocyclic substituted nitrogen, oxygen, and sulfur using carbazole as a starting material.

## Methods

### Chemistry

The chemicals and solvents used were obtained from commercial sources, and the solvents used were of analytical purity. The melting points of the compounds were determined with the SMP50 Automatic Melting Point device (Stuart, Florida, USA), and the values were given without correction. Aluminum plates coated with silica gel 60 F_254_ (Merck KGaA. Frankfurter, Germany) were used to check the purity of the compounds. The different solvent systems were used in thin-layer chromatography (TLC) such as hexane/ethyl acetate, dichloromethane/methanol, and dichloromethane/ethyl acetate. CAMAG^@^ UV 254 and 366 nm lamp (Munich, Germany) was used to monitor the reactions. Purification of the synthesized compounds was performed by crystallizing techniques. The purity of the compounds was checked by TLC and UPLC/MS-TOF analyses. The ^1^H-NMR spectra were recorded on Bruker 400-MHz spectrometer and are reported in ppm (δ) relative to tetramethylsilane (TMS) as the internal standard, and ^13^C-NMR (100 MHz) is referenced to CDCl_3_ and DMSO-*d*_6_. Chemical shifts were reported in ppm (parts per million) values. The coupling constants were given as Hertz (Hz). The HRMS spectra of the compounds were obtained from their solutions in methanol with positive ion (ESI + ) electrospray ionization techniques, using Waters LCT Premier XE UPLC/MSTOF system and MassLynx 4.1 software. Equity BEH C18 column (2.1 × 100 mm 1.7 µM, flow rate: 0.3 mL/min) was used as the stationary phase, and CH_3_CN/H_2_O (1–90%) gradient solvent system containing formic acid (0.1%) as the mobile phase. Whereas PerkinElmer Spectrum was for the analysis of FT-IR spectra and ChemDraw19 programs were used for the molecule drawing, MestReNova 12 program was used for the processing of NMR FID, LC-MS data, and analysis of the spectra.

### Synthesis of compounds 2–5

Compound **1** was synthesized according to the literature [36]. To a solution of the compound **1** (1 eq.) in THF (15 mL) was added Et_3_N (1.1 eq.) and appropriate sulfonyl chloride (1 eq.). The reaction mixture was stirred at rt for 18 h. then, the suspension was filtered and THF was removed from the filtrate. The raw product was dissolved in 3 mL of methanol and added to 150 mL of water. The precipitated solid was filtered, dried, and recrystallized using EtOH to give the desired product as colorless crystals.

### N′-(2-(9*H*-carbazol-9-yl) acetyl) methanesulfonohydrazide (2)

White solid; isolated yield: 87%, mp 287–288 °C; ^1^H NMR (400 MHz, DMSO) δ 10.79 (s, 1 H), 9.66 (s, 1 H), 8.17 (dd, *J* = 7.7, 1.0 Hz, 2 H), 7.57 (dd, *J* = 8.1, 1.1 Hz, 2H), 7.50–7.42 (m, 2H), 7.28–7.19 (m, 2H), 5.17 (s, 2H), 2.89 (s, 3H). ^13^C NMR (101 MHz, DMSO) δ 167.79, 141.04, 126.14, 126.03, 122.68, 120.67, 120.62, 119.56, 119.44, 109.88, 109.76, 44.39, 31.42. HRMS (m/z) [M + H]^+^ calcd for C_15_H_16_N_3_O_3_S: 318.0886, found: 318.0896.

### N′-(2-(9*H*-carbazol-9-yl) acetyl)-4-chlorobenzenesulfonohydrazide (3)

White solid; isolated yield: 90%, mp 272–273 °C; ^1^H NMR (400 MHz, DMSO) δ 10.82 (d, *J* = 1.7 Hz, 1H), 9.74 (d, *J* = 2.1 Hz, 1H), 8.18 (d, *J* = 8.2 Hz, 2H), 7.64–7.53 (m, 2H), 7.57–7.35 (m, 6H), 7.31–7.16 (m, 2H), 5.20 (s, 2H), 4.31 (s, 2H). ^13^C NMR (101 MHz, DMSO) δ 168.17, 141.03, 133.57, 133.27, 128.92, 128.76, 126.24, 126.15, 122.79, 120.73, 120.67, 120.63, 119.67, 119.57, 109.78, 57.34, 44.40. HRMS (m/z) [M+H]^+^ calcd for C_21_H_19_ClN_3_O_3_S: 428.0857, found: 428.0838.

### N′-(2-(9*H*-carbazol-9-yl) acetyl)-4-methoxybenzenesulfonohydrazide (4)

White solid; isolated yield: 85%, mp 238–240 °C; ^1^H NMR (400 MHz, DMSO) δ 10.61 (d, *J* = 2.9 Hz, 1H), 9.83 (d, *J* = 3.2 Hz, 1H), 8.14 (dd, *J* = 7.7, 1.0 Hz, 2H), 7.69 (d, *J* = 8.9 Hz, 2H), 7.47–7.37 (m, 5H), 7.22 (ddd, *J* = 7.9, 6.8, 1.3 Hz, 3H), 6.89 (d, *J* = 8.9 Hz, 2H), 4.98 (s, 2H), 3.73 (s, 3H). ^13^C NMR (101 MHz, DMSO) δ 166.51, 163.05, 140.85, 130.85, 130.34, 130.26, 126.08, 122.66, 120.57, 119.53, 114.47, 109.77, 55.94, 44.04. HRMS (m/z) [M+H]^+^ calcd for C_21_H_20_N_3_O_4_S: 410.1002, found: 410.0986.

### N′-(2-(9*H*-carbazol-9-yl) acetyl)-4-methylbenzenesulfonohydrazide (5)

White solid; isolated yield: 92%, mp 240–242 °C; ^1^H NMR (400 MHz, DMSO) δ 10.60 (d, *J* = 3.2 Hz, 1H), 9.90 (d, *J* = 3.1 Hz, 1H), 8.18–8.10 (m, 2H), 7.65 (d, *J* = 8.0 Hz, 2H), 7.48–7.36 (m, 4H), 7.22 (dd, *J* = 7.8, 5.1 Hz, 4H), 4.97 (s, 2H), 2.27 (s, 3H). ^13^C NMR (101 MHz, DMSO) δ 166.57, 143.69, 140.84, 135.96, 129.77, 128.13, 126.06, 122.67, 120.56, 119.52, 109.77, 44.08, 21.45. HRMS (m/z) [M +H]^+^ calcd for C_21_H_20_N_3_O_3_S: 394.1147, found: 394.1159.

### Synthesis of compounds 6 and 7

2-(9*H*-carbazol-9-yl) acetohydrazide (1 eq.) was dissolved in ethanol (20 mL), and then the requisite isothiocyanate (1 eq.) was added in the equivalent molar ratio to this solution. The reactions that occurred at the boiling temperature of the solvent were completed after 5 h. The obtained solid after the removal of the solvent in vacuo was purified via recrystallization using ethanol.

### 2-(2-(9*H*-carbazol-9-yl) acetyl)-N-phenylhydrazine-1-carbothioamide (6)

White solid; isolated yield: 88%, mp 235–237 °C; ^1^H NMR (400 MHz, DMSO-*d*_6_) δ 10.52 (s, 1H), 9.86–9.67 (m, 2H), 8.17 (d, *J* = 7.7 Hz, 2H), 7.61 (d, *J* = 8.1 Hz, 2H), 7.47 (t, *J* = 8.1 Hz, 4H), 7.39 (q, *J* = 7.6 Hz, 2H), 7.24 (tt, *J* = 7.0, 3.3 Hz, 3H), 5.18 (d, *J* = 2.1 Hz, 2H). ^13^C NMR (101 MHz, DMSO) δ 184.16, 167.77, 141.09, 139.50, 128.70, 126.14, 126.02, 122.75, 120.65, 119.58, 109.96, 44.56, 40.64, 40.43, 40.22, 40.01, 39.80, 39.59, 39.39. HRMS (m/z) [M+H]^+^ calcd for C_21_H_19_N_4_OS: 375.1201, found: 375.1240.

### 2-(2-(9*H*-carbazol-9-yl) acetyl)-N-(4-chlorophenyl) hydrazine-1-carbothioamide (7)

White solid; isolated yield: 90%, mp 244–246 °C; ^1^H NMR (400 MHz, DMSO-*d*_6_) δ 10.49 (s, 1H), 9.75 (d, *J* = 28.4 Hz, 2H), 8.16 (d, *J* = 7.7 Hz, 2H), 7.60 (d, *J* = 8.2 Hz, 2H), 7.53 (d, *J* = 8.8 Hz, 2H), 7.50–7.39 (m, 4H), 7.23 (t, *J* = 7.4 Hz, 2H), 5.17 (s, 2H). ^13^C NMR (101 MHz, DMSO) δ 171.38, 167.88, 141.20, 141.06, 138.50, 128.62, 126.14, 126.02, 122.73, 120.66, 120.62, 119.59, 119.44, 109.95, 109.83, 44.54. HRMS (m/z) [M +H]^+^ calcd for C_21_H_18_ClN_4_OS: 409.1492, found: 409.1520.

### Synthesis of compounds 8 and 9

A mixture of compound **6** or **7** (1 eq.), ethyl bromoacetate (1 eq.), and fused sodium acetate (4 eq.) were heated under reflux for 3 h in anhydrous ethanol (25 mL). The reaction mixture was cooled, diluted with water (75 mL), and allowed to stand overnight. The precipitate was filtered, dried, and recrystallized from ethanol.

### 2-(9*H*-carbazol-9-yl)-N′-(4-oxo-3-phenylthiazolidin-2-ylidene) acetohydrazide (8)

White solid; isolated yield: 73%, mp 272–274 °C; ^1^H NMR (400 MHz, DMSO) δ 11.37 (s, 1H), 8.17 (dd, *J* = 7.6, 1.0 Hz, 2H), 7.68 (d, *J* = 8.2 Hz, 2H), 7.47–7.36 (m, 4H), 7.27–7.15 (m, 3H), 6.99–6.90 (m, 2H), 5.42 (d, *J* = 17.3 Hz, 1H), 5.27 (d, *J* = 17.3 Hz, 1H), 4.18 (s, 2H). ^13^C NMR (101 MHz, DMSO) δ 168.77, 167.13, 152.16, 147.79, 141.02, 129.90, 126.13, 125.05, 122.84, 121.19, 120.61, 119.69, 110.06, 44.53, 30.46. HRMS (m/z) [M +H]^+^ calcd for C_23_H_19_N_4_O_2_S: 415.1250, found: 415.1255.

### 2-(9*H*-carbazol-9-yl)-N′-(3-(4-chlorophenyl)-4-oxothiazolidin-2-ylidene) acetohydrazide (9)

White solid; isolated yield: 70%, mp 287–289 °C; ^1^H NMR (400 MHz, DMSO) δ 11.38 (s, 1H), 8.17 (d, *J* = 7.7 Hz, 2H), 7.66 (d, *J* = 8.2 Hz, 2H), 7.50–7.34 (m, 4H), 7.23 (t, *J* = 7.5 Hz, 2H), 6.94 (dd, *J* = 8.6, 1.3 Hz, 2H), 5.41 (d, *J* = 17.2 Hz, 1H), 5.27 (d, *J* = 17.3 Hz, 1H), 4.20 (s, 2H). ^13^C NMR (101 MHz, DMSO) δ 168.75, 167.15, 153.15, 146.68, 141.00, 129.90, 129.10, 126.14, 123.08, 122.83, 120.62, 119.71, 110.04, 44.51, 30.59. HRMS (m/z) [M+ H]^+^ calcd for C_23_H_18_ClN_4_O_2_S: 449.0761, found: 449.0876.

### Synthesis of compound 10

To a solution of the hydrazide (compound **1**) (1 eq.) in DMF (5 mL) was added CDI (1.1 eq.) and stirred at rt for 3 h. The reaction mixture was then poured onto water and filtered off to give the crude product, which was purified via recrystallization using methanol.

### 5-((9*H*-carbazol-9-yl)methyl)-1,3,4-oxadiazol-2(3 H)-one (10)

White solid; isolated yield: 85%, mp 209–210 °C; ^1^H NMR (400 MHz, DMSO) δ 13.40 (s, 1H), 8.20 (d, *J* = 7.8 Hz, 2H), 7.72 (d, *J* = 8.2 Hz, 2H), 7.57–7.49 (m, 3H), 7.34–7.21 (m, 2H), 5.81 (s, 2H). ^13^C NMR (101 MHz, DMSO) δ 161.80, 154.57, 140.38, 126.49, 123.02, 120.82, 120.28, 110.16, 38.59, 30.90. HRMS (m/z) [M +H]^+^ calcd for C_15_H_12_N_3_O_2_: 266.0851, found: 266.0917.

### Synthesis of compound 11

To a solution of compound **10** (1 eq.) in DMF (10 mL) was added Et_3_N (1.1 eq.) and 4-fluoro benzoyl chloride (1 eq.) stirred at rt for 8 h. The reaction mixture was then poured onto water and filtered off to give the crude product, which was purified via recrystallization using ethanol.

### 5-((9*H*-carbazol-9-yl)methyl)-3-(4-fluorobenzoyl)-1,3,4-oxadiazol-2(*3H*)-one (11)

White solid; isolated yield: 89%, mp 190–192 °C; ^1^H NMR (400 MHz, DMSO) δ 8.19 (dd, *J* = 7.7, 1.1 Hz, 2H), 7.91–7.83 (m, 2H), 7.74 (d, *J* = 8.2 Hz, 2H), 7.58–7.49 (m, 2H), 7.33–7.24 (m, 4H), 5.81 (s, 2H). ^13^C NMR (101 MHz, DMSO) δ 166.55, 164.04, 163.16, 153.26, 149.83, 140.43, 133.72, 133.62, 127.98, 127.95, 126.45, 123.02, 120.81, 120.22, 120.13, 115.78, 115.56, 110.23, 109.95, 38.54. HRMS (m/z) [M+ H]^+^ calcd for C_22_H_15_FN_3_O_3_: 388.1019, found: 388.1086.

## Biological methods

### Cell culture and MTS assay

Hepatocellular carcinoma (Hep3B and HepG2), cervical adenocarcinoma (HeLa), breast carcinoma (MCF-7), melanoma (B16F1), colorectal adenocarcinoma (Caco-2), and colon adenocarcinoma (Colo205), as well as human hepatic stellate (LX-2), were used as cancer and normal cell lines and were cultured in RPMI-1640 media and supplemented with 10.0% fetal bovine serum, 1.0% l-Glutamine, and 1.0% Penicillin/Streptomycin antibiotics. After that, the cells matured in a moist atmosphere with 5.0% CO_2_ at 37 °C. In a 96-well plate, the cells were seeded at 2.4 × 104 cells/well. After 72 h, the cells were confluent, the media was changed, and then the cells were incubated at various concentrations (250, 100, 50, 10, and 1 µM). The viability of cells was assessed by the Cell Tilter 96® Aqueous One Solution Cell Proliferation (MTS) Assay according to the manufacturer’s procedures (Promega Corporation, Madison, WI). However, at the end of the treatment, about 20 µL/100 µL of MTS solution/media was added to each well and for 2 h, they were incubated at 37 °C. Finally, the absorbance was measured at 490 nm [37].

### Antioxidant activity method (in-vitro)

The free 2,2-diphenyl-picrylhydrazyl (DPPH) radical scavenging assay was employed to measure the antioxidant activity of the Carbazole derivatives. A stock solution (1000 µg/mL) of each derived molecule was prepared in methanol. In addition, a solution of Trolox (1000 µg/mL) was also prepared (the reference drug). A dilution series was prepared from the stock solutions for each compound, giving seven serial dilutions at 1000, 300, 100, 50, 10, and 1 µM. One mL of each compound dilution was mixed with 1.0 mL 0.002 g/mL DPPH in methanol. One mL of methanol was added to give a final working volume of 3.0 mL. The DPPH solution was freshly prepared, as it was very sensitive to light. The blank control of the series concentrations was DPPH in methanol in a ratio of 1:2, without the addition of any compound. All working solutions were incubated at room temperature (25 ^°^C) in the dark for about 30 min. The optical densities were then measured with a spectrophotometer at a wavelength of 517 nm. The following equation was used to calculate the percent DPPH inhibition for each plant fraction, with Trolox as the standard compound:

DPPH inhibition % = (AB − Ats)/AB × 100%.

where AB is the recorded absorbance of the blank solution, and Ats is the recorded absorbance of the tested sample solution [38].

### Computational details

The optimized structures of the **2**–**5** and **8**–**11** compounds were computed and constructed using the Gaussian 09 W package and Gauss View 5.0 tools, utilizing the DFT/B3LYP theory/functional and 6-311 + + G(d,p) basis set [39, 40]. The AutoDock Vina application was used to perform in silico docking computations [41], and the CB-Dock server was utilized to support docking scores [42, 43]. Then, the SwissADME [44–46] was utilized to determine drug similarity or likeness, as well as some ADMET attributes. Finally, the pkCSM web server [47, 48] was used to perform the toxicity assessment [47].

### Drug-likeness prediction and ADME/T analyses

The Pfizer Rule of Five is a rule of thumb for determining whether an inhibitor with specific biological and pharmacological features would be an orally active medicine in the human body [49, 50]. To assure the safety profile of candidate compounds in drug development, preclinical safety, and pharmacokinetics investigations are required. Despite the extensive use of in vivo and in vitro tests, experimental evaluations have time and expense limitations. For each of these preclinical endpoints, in silico projections have been made during the last few decades. However, only a few web-based tools, have combined several models into a simple one-step platform to assist researchers in accurately evaluating possible drug candidates [49].

### Molecular dynamics (MD) simulations

The online WEBGRO Macromolecular Simulations server (https://simlab.uams.edu/ProteinWithLigand/index.html) was utilized to conduct molecular dynamics simulations based on GROMAC. It is a common public service that includes a GRACE High-Performance Computing Facility administrated by the University of Arkansas for Medical Sciences (UAMS) [51, 52]. At first, the ligand topology was created utilizing the PRODRUG 2.3 online server (http://davapc1.bioch.dundee.ac.uk/cgi-bin/prodrg/submit.html) at GROMOS96 54a7 force field [53]. The prepared prodrug structures and their related ligand-protein complexes were subjected to molecular dynamics simulations using the Protein with Ligand Simulation tool integrated into the WEBGRO server. The GROMOS96 54a7 force field was used to describe the interactions between the atoms in the system. The SPC water model was used to solvate the system, and a triclinic box was used to contain the system. Na + and/or Cl− ions were added as salt types to neutralize the system. The incorporated structures (9, 10, and 11) and complexes were energetically minimized to a maximum of 5000 steps using the steepest descent integrator. In terms of equilibration and MD run parameters, NVT/NPT at 300 K, 1.0 bar pressure, and Leap-frog MD integrator type were chosen. The number of frames per MD simulation for each system was set to 5000 and performed for a simulation time of 50 ns. As a result, various trajectories such as the radius of gyration (Rg) and the root-mean-square deviation (RMSD) were obtained, which were used to estimate the formation and stability of complexes within XDH binding pocket [54, 55].

## Results and discussion

### Chemistry

**Scheme 1:**
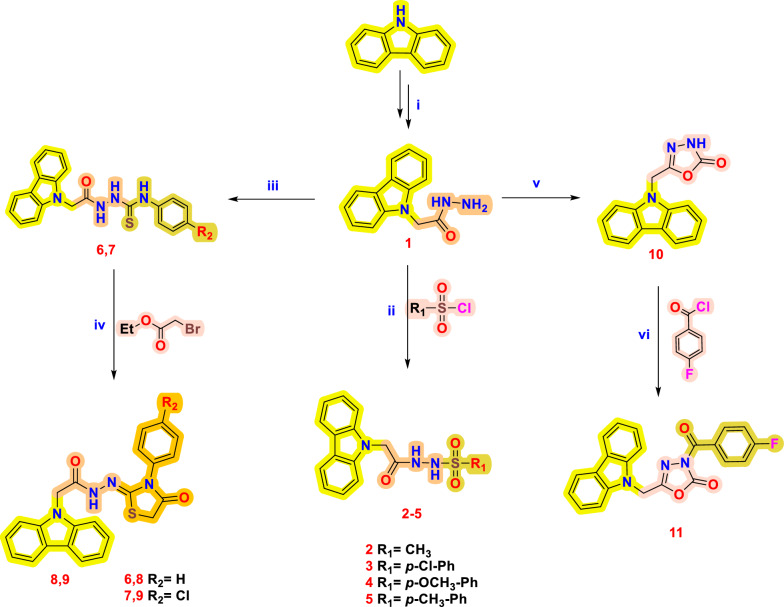
(**i**) Ethyl bromoacetate, NaH, DMF; then, hydrazine hydrate (80%), ethanol, reflux, 24 h; (**ii**) sulfonyl chloride, Et_3_N, THF, rt, 18 h; (**iii**) -Ph (or *p*-Cl-Ph) isothiocyanate, ethanol, reflux, 3 h; (**iv**) Ethyl bromoacetate, sodium acetate, ethanol, reflux, 3 h; (**v**) CDI, DMF, rt, 1d. (**vi**) 4-Fluorobenzoyl chloride, Et_3_N, DMF, 4 h

In the general reaction Scheme1, the synthesis steps of compounds by four different functional groups with a carbazole skeleton performed in the study are observed. The optimal reaction times were determined by following the reaction with TLC and LC-MS. The crystallization technique was used to purify all compounds. HRMS, ^1^H-, ^13^C_APT_-NMR spectra of all synthesized compounds are presented in the Additional file section. When the HRMS spectra are examined, the calculated and found [M+H]^+^ ion signals of all final products were seen to be completely compatible.

We started the synthesis steps with carbazole, which is a starting compound with known biological activity and fluorescence properties. The intermediate product with the ester functional group formed because of the reaction of carbazole in DMF with ethyl bromoacetate accompanied by a strong base such as NaOH was synthesized without additional purification. In the next step, the reaction of hydrazine hydrate with this product was carried out according to the literature [36]. Firstly, the reactions of some sulfonyl chlorides (-methyl, *p*-chlorophenyl, *p*-methoxyphenyl, and *p*-methyl phenyl sulfonyl chloride) with compound **1** were carried out in THF accompanied by Et_3_N and at room temperature. In the ^1^H-NMR spectra of sulfonohydrazide compounds obtained with very high yields, characteristic (–NH–NH–) doublet signals, which have a total of two protons, are observed at 9–11 ppm.

Secondly, because of the addition reactions of phenyl and *p*-chlorophenyl isothiocyanate and compound **1** in ethyl alcohol, compounds **6** and **7** were synthesized, respectively. In the ^13^C_APT_-NMR spectrum of this compound, which has a thiosemicarbazide functional group, the carbonyl carbon (C= O) and the thion carbon (C= S) signals are observed at 167 and 184 ppm, respectively. In the next step, compounds **8** and **9**, each having a thiazolidine ring, were synthesized from the reactions of compounds **6** and **7** with ethyl bromo acetate in the presence of sodium acetate. In the ^1^H-NMR spectra of compounds **8** and **9**, we observed that the methylene protons present in the thiazolidine ring are different, and each of their signals is a doublet at 5.44–5.24 ppm.

Finally, because of the reaction of compound **1** with CDI (carbonyldiimidazole) in DMF medium at room temperature, compound **10** was obtained, from which the oxadiazole-one ring system was synthesized. The derivative reaction with *p*-fluorobenzoyl chloride was carried out on this compound. The disappearance of the NH signal at 13.40 ppm in the ^1^H-NMR spectrum and an increase in the amide carbonyl carbon signal in the ^13^C_APT_-NMR spectrum are important indicators (all specturms of NMR and HRMS were provided in the Additional file 1: Figures S1–S31).

### Biological evaluations

#### Cytotoxic evaluation of the compounds (2–5, 8–11)

To evaluate the antiproliferative activities of the synthesized compounds, the MTS assay was performed on B16-F1, Colo205, HepG2, Hep3B, CaCo-2, HeLa, and MCF7 cells. As shown in Table 1, solutions of various concentrations were used. Based on the results given in Table 1, carbazole derivatives containing oxadiazole (compounds **10** and **11**) were found to have a stronger antiproliferative effect than those containing benzenesulfonohydrazide (compounds **2**–**5**). Compound **10** with IC_50_ values of 7.68 and 10.09 µM, respectively, showed strong activity against HepG2 and HeLa cancer cell lines. Compounds **10** was found to be the strongest compound against the MCF7 cancer cell line, with an IC_50_ of 6.44 µM. Moreover, compound **9** showed strong activity against HeLa cancer cell lines with an IC_50_ value of 7.59 µM. However, except for compound **5**, all of the synthesized compounds showed moderate antiproliferative activities against CaCo-2 with IC_50_ values in the range of 43.7–187.23 µM. All of these values were compared with the IC_50_ values of the positive control 5-FU anticancer drug.

**Table 1:** IC_50_ (µM) of carbazole derivatives on various cell lines

Cell line/ code	IC_50_ (µM)							
	2	3	4	8	9	10	11	5-FU
B16F1	Ni	Ni	Ni	107.65±	40.22 ± 1.25	**Ni**	118.30 ± 1.08	83.43 ± 2.50
Colo205	Ni	Ni	Ni	Ni	252.01 ± 2.58	173.304 ± 2.41	Ni	12.04 ± 1.87
HepG2	Ni	Ni	Ni	37.62 ± 2.02	107.07 ± 2.42	**7.686 ± 1.07**	18.91 ± 1.26	3.85 ± 0.75
CaCo-2	187.23 ± 2.47	71.23 ± 2.04	46.83 ± 1.58	95.37 ± 1.08	86.35 ± 1.015	73.80 ± 2.11	43.7 ± 2.33	7.08 ± 0.54
HeLa	Ni	Ni	210.11 ± 2.19	64.94 ± 1.85	**7.59 ± 0.89**	**10.09 ± 0.78**	17.17 ± 1.02	1.26 ± 0.27
Hep3B	Ni	Ni	Ni	152.85 ± 1.49	171.86 ± 2.07	38.335 ± 2.11	22.16 ± 1.75	23.44 ± 1.71
MCF-7	Ni	Ni	79.70 ± 2.07	19.18 ± 1.05	18.16 ± 1.45	18.41 ± 0.29	**6.44 ± 1.25**	1.82 ± 0.88
LX-2	Ni	Ni	Ni	90.09 ± 2.15	4.67 ± 1.80	Ni	**2.87 ± 0.57**	15.92 ± 0.95

Bold values indicates to the most potent compound and cell lines

*Ni* no inhibition or the IC_50_ values >250 µM

*P*-value ≤ 0.05

**Fig. 2:**
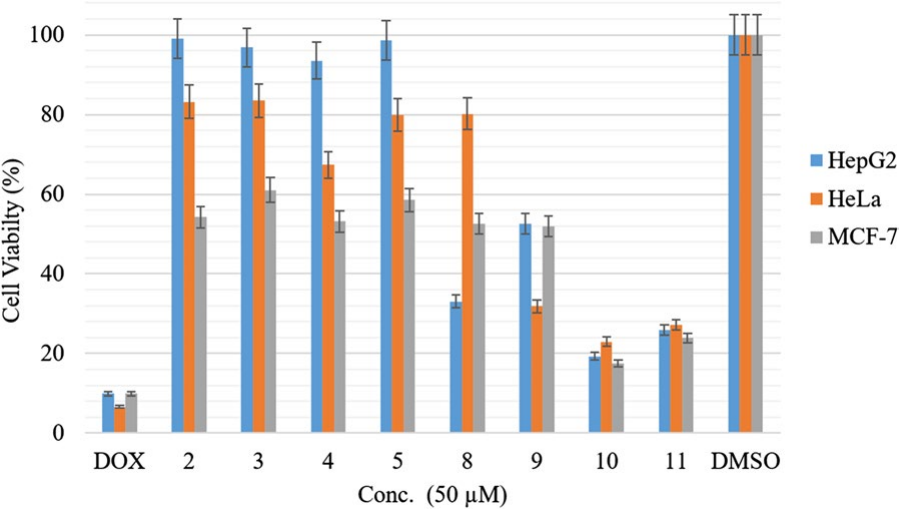
Cell viability percentages against HeLa, HepG2, and MCF-7 for all synthesized compounds versus Dox (positive control) and DMSO (negative control)

Percent cell viability was calculated for MCF-7, HeLa, and HepG2 cancer cells at 50 µM concentrations. As shown in Fig. 2, the positive control Doxorubicin (Dox) was compared with the negative control (DMSO). The percent viability of HepG2 versus heterocyclic compounds was found to be below 53%. The most active compound (compound **10**) had a value very close to the positive control DOX, while the percentages for the other group were very close to the negative control. For all synthesized compounds, the percentage of cell viability against MCF-7 cancer cell lines was determined to be below 61%, and compounds **10** and **11** were found to be the most active compounds.

#### Anti-fibrotic activities of the synthesized compounds

**Fig. 3:**
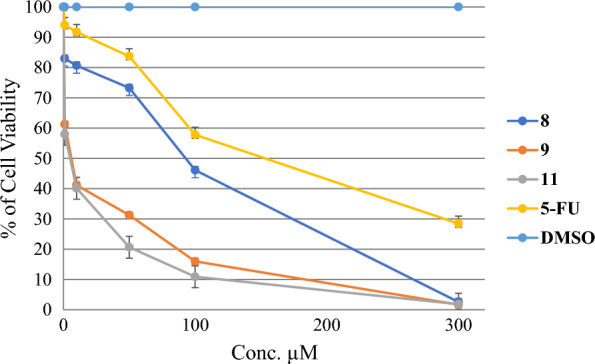
The cell viability of the LX2 cell line after treatment with the synthesized **9–11** compounds and positive control 5-FU

To investigate the anti-fibrotic effects of these compounds on the human hepatic stellate cell (HSC) line LX-2, the viability of LX-2 cells following various compounds’ treatment was determined by the MTS assay. Except for compounds **10** and **11**, most of the synthesized compounds showed weak or negligible anti-fibrotic activities and showed very potent activities against this liver cell line with IC_50_ values 2.87 ± 0.57 and 4.67 ± 1.80 µM. The most active compounds were selected, presented, and the cell viability was calculated for these compounds at different concentrations in comparison with positive control 5-FU (Fig. 3). The most potent was found in compound **11** and the cellular viability on LX-2 was found at 57.96% at 1 µM concentration in comparison with the positive control 5-FU cell viability value of 94.02%. The results suggested that these compounds have better anti-fibrotic activities than 5-FU at 1 µM concentration, and further biological investigation into the LX2 cell line is requested shortly.

#### Antioxidant activity

**Fig. 4:**
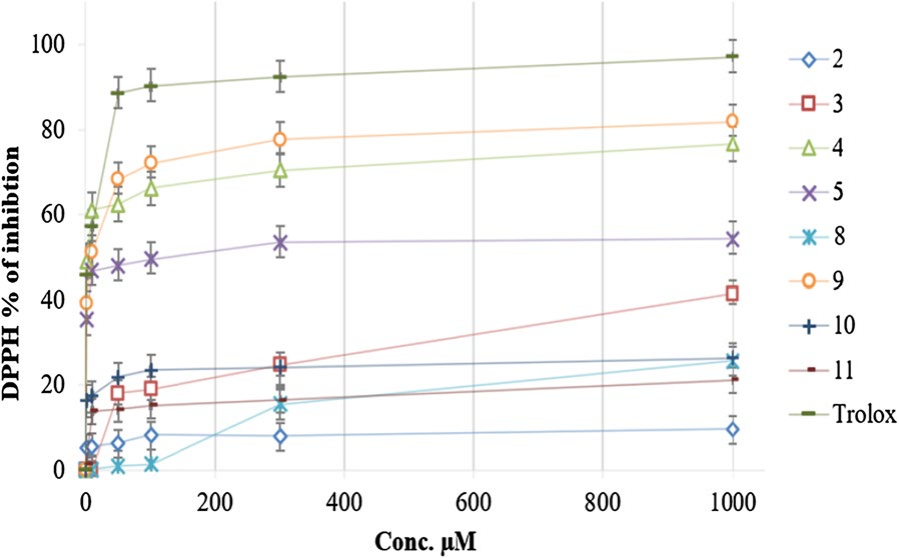
The percentage of inhibition against DPPH after treatment with different concentrations of synthesized compounds (**2**–**5**, **8**–**11**) and positive control Trolox

Evaluation of the free radical scavenging activity of compounds (**2**–**5**, **8**–**11**) synthesized using Trolox as the reference antioxidant agent was expressed as percent DPPH inhibition (Fig. 4). Among the compounds evaluated, two compounds (**4** and **9**) showed potent antioxidant activities against DPPH with IC_50_ values of 1.05 ± 0.77 and 5.15 ± 1.01 µM, respectively, compared with Trolox (IC_50_ = 2.08 ± 0.57 µM).

#### Molecular docking analysis

The goal of computer-aided drug design is to help with drug candidate research and discovery by lowering the cost of the drug design process. In silico methodologies, technological advancements in this subject are useful in delivering speedier optimization and identification procedures. Modern drug development relies heavily on structure-based computational modeling of ligand-receptor interactions. Molecular docking calculations are frequently employed in structure-based drug design studies to discover conformational changes that vary with the environment and to characterize the interaction of the molecule with the protein with which it interacts within the body. In other words, docking is a molecular modeling technique that assures predicting how a small molecule interacts with macromolecules such as a protein with a simple determination [56–58]. However, this simple method gave very good results for initial calculations and it is preferred for this section. To begin, the structures of **2**–**5** and **8**–**11** ligands were optimized in the gas phase using DFT/B3LYP theory/functional and 6-311 + + G(d,p) basis set via Gaussian 09 W package and Gauss View 5.0 programs [39, 40] and their PDB forms were generated. At this stage, the sum of electronic and zero-point energy values of the optimized ligands (**2**–**5** and **8**–**11**) with Gaussian 09 W were obtained as − 1368.70874469 a.u., − 2020.03956005 a.u., − 1674.97393828 a.u., − 1599.76801021 a.u., − 1654.96329614 a.u., 2114.55523961 a.u., − 892.95192843 and − 1336.57245190 a.u., respectively. In other words, as a result of the calculation, it was seen that the most stable structure was 9 molecules. Then, taking into consideration the torsion angles in the structure, the pdbqt forms of the ligands were recorded in the same file with the help of Discover Studio Visualizer 4.0 (DSV 4.0) software. In the next step, XDH/PDB: 3UNI [59] and PPARG/PDB: 3VSO [60] targets were selected for docking following the literature [59–61]. The XDH gene codes for xanthine dehydrogenase, an enzyme that breaks down xanthine. Purines, which are building blocks of DNA and its chemical cousin, RNA, are broken down normally by this enzyme [62]. On the other hand, the PPARG gene in humans encodes the peroxisome proliferator-activated receptor gamma, also known as the glitazone reverse insulin resistance receptor or NR1C3. It is a type II nuclear receptor that functions as a transcription factor [63]. Here, the PDB structures of these receptors (PDBs: 3UNI and 3VSO) were downloaded from RCSB (Protein Data Bank). Within receptors, hetero groups (water and other co-ligands) were removed, and polar hydrogen bonds were added and re-saved with Discover Studio Visualizer 4.0 (DSV 4.0) software. Just before the docking calculations, the active residues in the receptors were detected as follows: SER1082, VAL1081, ALA1079, ALA1078, ILE1063, TYR1062, LEU1014, VAL1011, THR1010, PHE1009, SER1008, ALA910, THR909, SER907, ILE877, ASP872, ARG871, SER870, ASN869, ILE835, MET826, CYS825, ARG839, ARG804, GLU802, GLY799, ARG793, GLN767, ILE666, HIS665, PHE604, ASP594, LEU580, ASP461, ARG394, TYR393, ILE353, LYS271, ILE264, LEU257, LYS256, CYS148, CYS73, CYS51, GLU45, GLY44, CYS43, GLY42 for XDH/PDB: 3UNI; TYR473, LEU469, LEU453, HIS449, MET364, MET348, TYR327, HIS323, SER289, ARG288, GLN286, CYS285, GLY284, PHE282, PHE264 for PPARG/PDB: 3VSO. Thus, the grid parameters to be used in the calculation were determined as follows, including the active residues: 90 × 100 × 108 Å3 for x, y, z dimensions, 0.375 space, and 29.81, 50.17, 98.399 for x,y,z centers and 34 × 38 × 50 Å3 for x, y, z dimensions, 0.375 space, and 16.474, 70.389, 14.83 for x,y,z centers for XDH/PDB: 3UNI and PPARG/PDB: 3VSO, respectively. After molecular docking, the binding energies, inhibition constant (K_i_) values, and the number of hydrogen bonds are given in Table 2.

**Table 2:** Molecular docking scores of compounds **2**–**5** and **8**–**11**

Compounds	XDH/PDB: 3UNI (A chain)			PPARG/PDB: 3VSO (A chain)		
	Binding Energy with Vina (kcal/mol)	K_i_ values (μM) with Vina	The number of Hydrogen Bonding	Binding Energy with Vina (kcal/mol)	K_i_ values (μM) with Vina	The number of Hydrogen Bonding
**2**	− 10.1	0.039505	5	− 8.6	0.496769	1
**3**	− 11.3	0.005212	5	− 9.4	0.128751	2
**4**	− 9.8	0.065546	3	− 9.5	0.108755	2
**5**	− 9.0	0.252902	3	− 9.5	0.108755	1
**8**	− 9.4	0.128751	2	− 10.3	0.028187	2
**9**	− 10.1	0.039505	4	− 10.6	0.016988	3
**10**	− 9.9	0.055367	2	− 8.3	0.824244	3
**11**	− 10.9	0.010239	3	− 11.1	0.007306	0

**Fig. 5:**
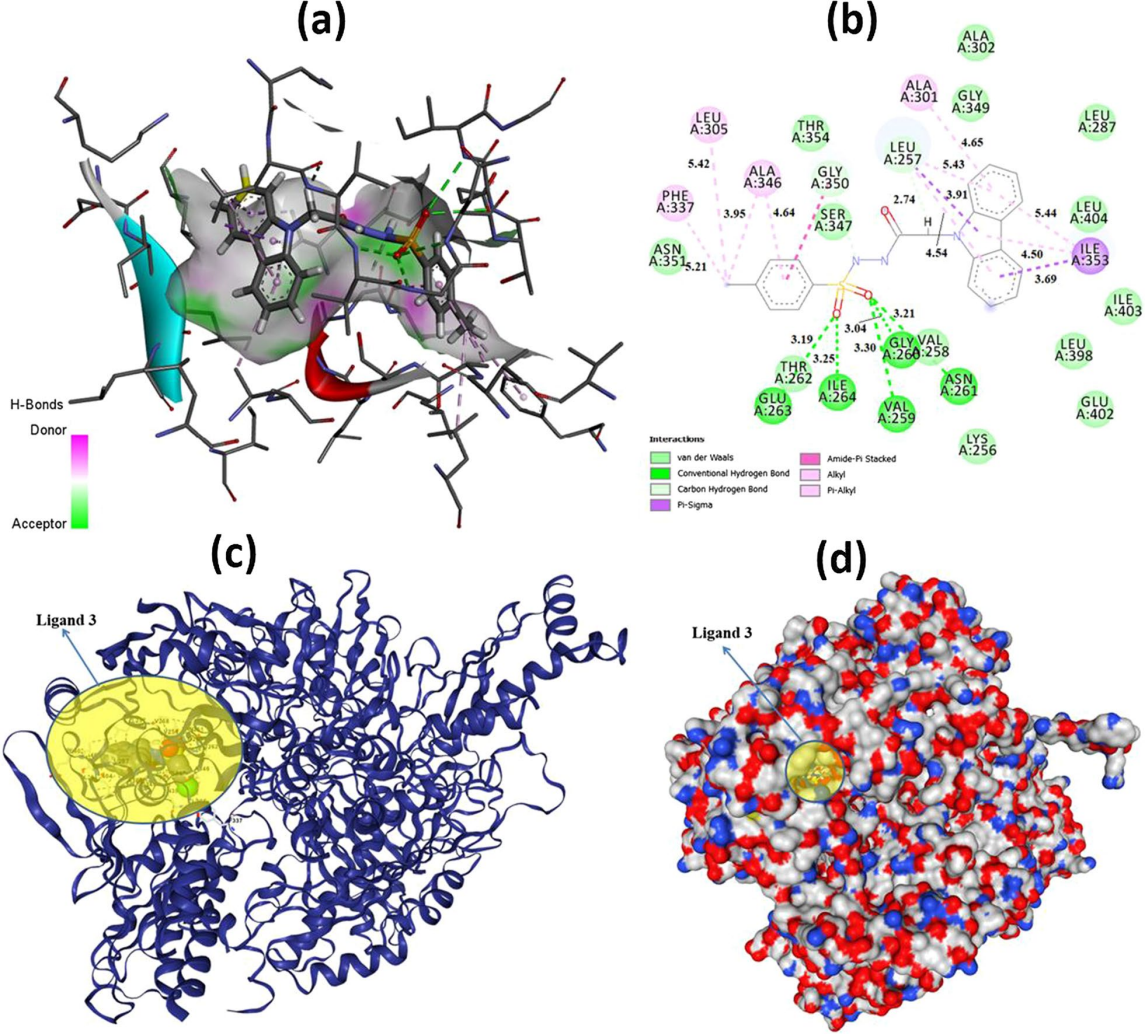
3D (**a**) and 2D (**b**) molecular docking results with Autodock Vina and cartone (**c**) and surface (**d**) forms with CB-Dock for 3 + XDH/PDB: 3UNI (A Chain)

Now let’s interpret the results obtained for XDH/PDB: 3UNI and PPARG/PDB: 3VSO, respectively: When XDH/PDB: 3UNI is selected as the receptor, according to the obtained results as shown in Table 2, theoretically the most active molecule is 3 with − 11.3 kcal/mol binding energy and 0.005212 M inhibition constant and its potential to inhibit this protein is quite high. When the obtained results were compared according to their binding energies, the following order was reached: 3 > 11 > 2 = 9 > 10 > 4 > 8 > 5. Since compound 3 is the most active molecule in the calculations, the interpretation of the interactions was made on this molecule and docking outcomes are given in Fig. 5 as 3D (a), 2D (b), cartone (c), and surface forms (d).

**Fig. 6:**
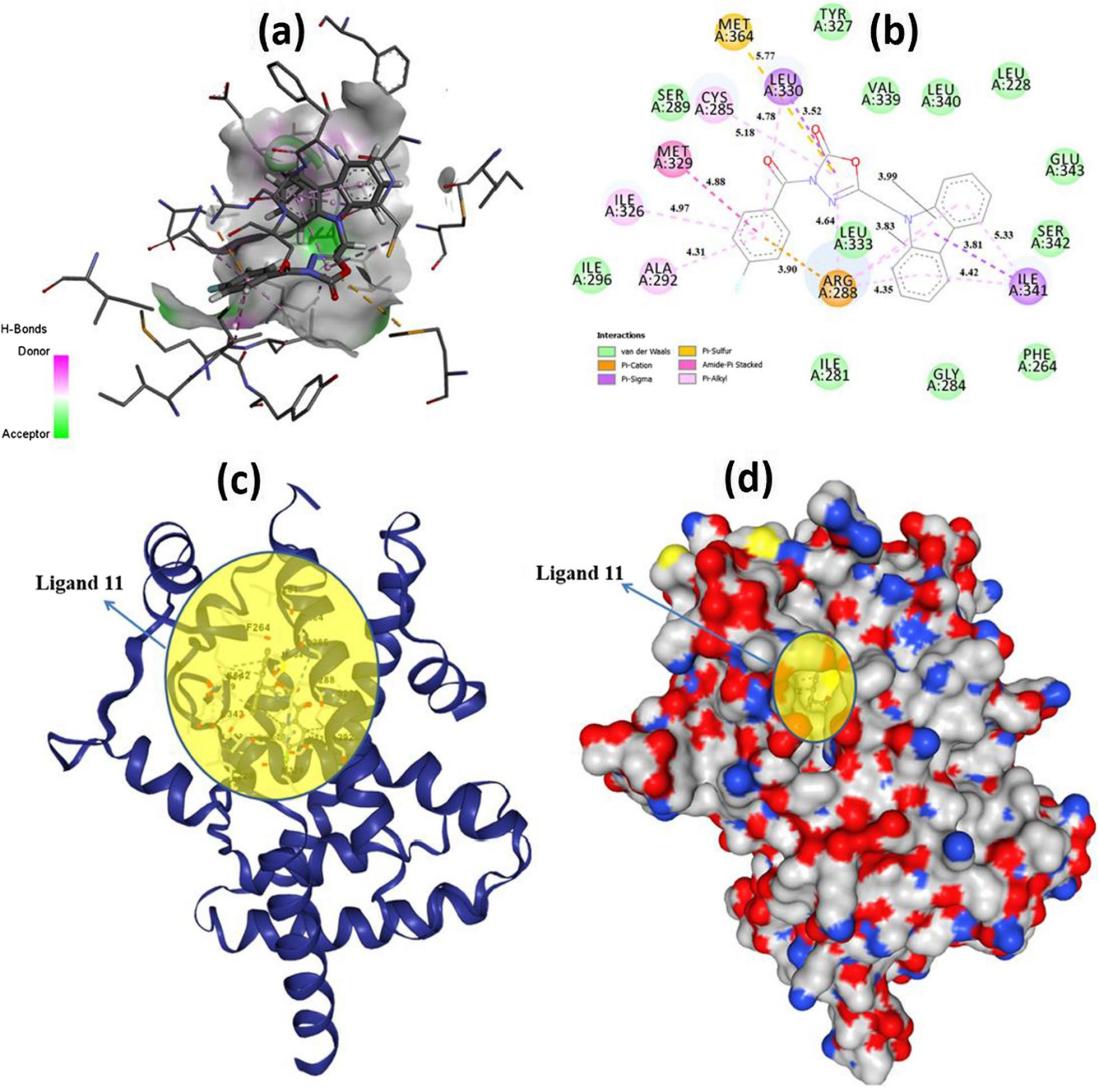
3D (**a**) and 2D (**b**) molecular docking results with Autodock Vina and cartone (**c**) and surface (**d**) forms with CB-Dock for 11 + PPARG/PDB: 3VSO (A Chain)

As seen in Fig. 6, five conventional hydrogen bonds were observed between THR262 and O with 3.19 Å, ILE264, and O with 3.25 Å, VAL259, and O with 3.30 Å, GLY260, and O with 3.04 Å, ASN261, and O with 3.21 Å. Apart from these interactions from Fig. 4 (b), van der Waals, carbon-hydrogen bond, π-sigma, amide-π-stacked, alkyl, and π-alkyl interactions and their bond lengths were observed clearly. Additionally, for 2, 4, 5, 8, 9, 10, and 11 + XDH/PDB: 3UNI (A Chain), the docking interactions are given as Additional file 1: Figs. S32–S38, respectively. Secondly, when PPARG/PDB: 3VSO is selected as the receptor, according to the obtained results as shown in Table 2, theoretically the most active molecule is **11** with − 11.1 kcal/mol binding energy and 0.007306 M inhibition constant, and its potential to inhibit this protein is quite high. When the obtained results were compared according to their binding energies, the following order was reached: 11 > 9 > 8 > 4 = 5 > 3 > 2 > 10. Since compound **11** is the most active molecule in the calculations, the interpretation of the interactions was made on this molecule and docking outcomes are given in Fig. 6 as 3D (a), 2D (b), cartone (c), and surface (d).

**Fig. 7:**
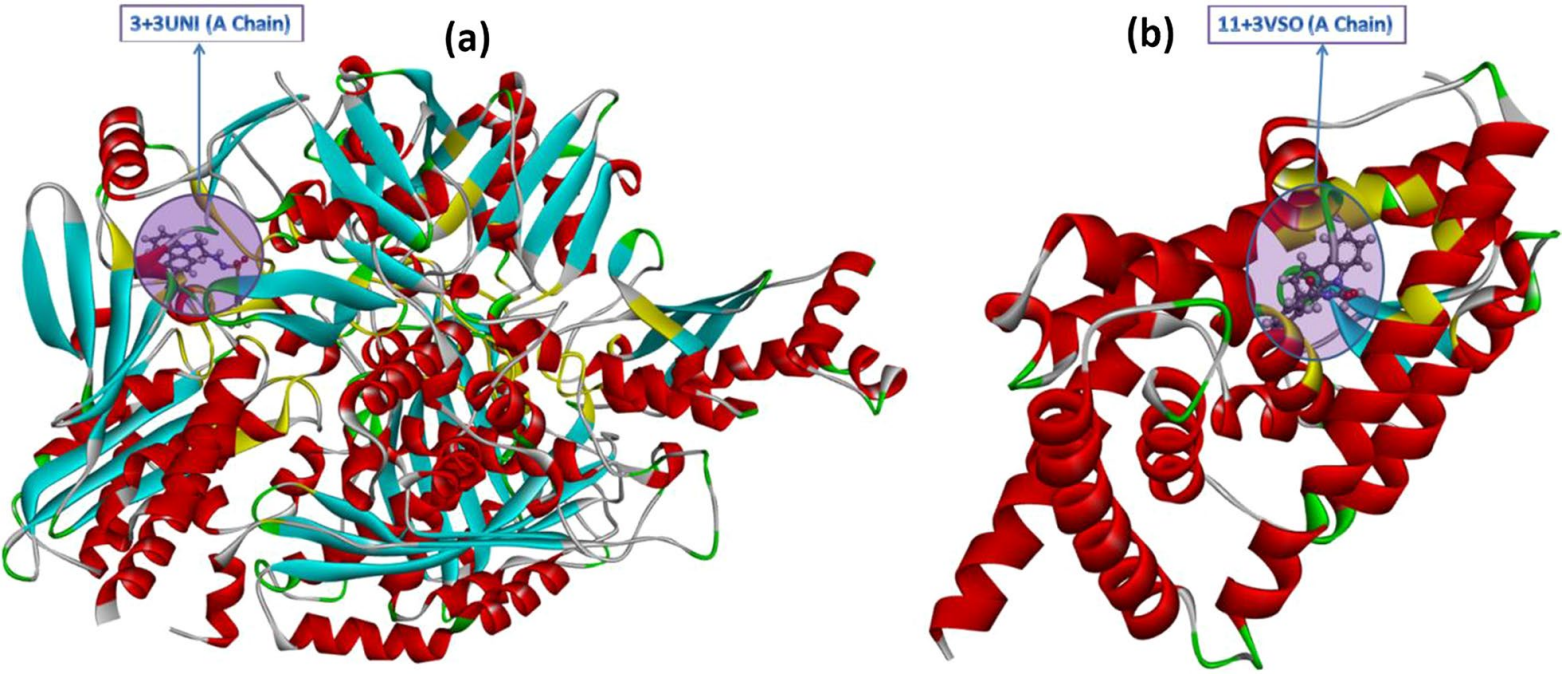
The placement within XDH/PDB: 3UNI (A Chain) and PPARG/PDB: 3VSO (A Chain) of **3** and **11** molecules

As seen in Fig. 5, the conventional hydrogen bonding could not be observed, but van der Waals, π-cation, π-sigma, π-sulfur, amide-π-stacked, and π-alkyl interactions and their bond lengths were determined. Furthermore, for 2, 3, 4, 5, 8, 9, and 10 + PPARG/PDB: 3VSO (A Chain), the docking interactions as 3D and 2D are shown in Additional file 1: Figs. S39–S45, respectively. Regarding 3 + XDH/PDB: 3UNI and 11 + PPARG/PDB: 3VSO interactions, we can see in detail and clearly how molecules attach to proteins in Fig. 7.

Finally, we think that the calculated Ki values in Table 2 are based on the Ki = exp(ΔG/RT) formula (G: binding energy, R: gas constant = 1.987203610-3 kcal/mol, and T: rt = 298.15 K) and that the results will guide more advanced simulation techniques in the future.

#### Drug-likeness prediction and ADME/T analyses

To be effective with this strategy, a preclinical evaluation platform must not only anticipate critical ADME/T (absorption, distribution, metabolism, excretion, and toxicity) features but also provide recommendations on how to ameliorate the undesirable characteristics. If two or more of these thresholds are met, a ligand or inhibitor can be absorbed/active orally. These rules can be classified into the following categories: a) Molecular mass or weight (MW) ≤ 500 g/mol, b)The n–octanol/water partition coefficient (high lipophilicity: MLogP) ≤ 5, c) Number of H-bond acceptor (HBA) ≤ 10 and donors (HBD) ≤ 5, d) Number of rotatable bonds (nRot) ≤ 10 and e) TPSA: topological polar surface area < 140 Å². In this section, these parameters were investigated using the SwissADME web page [50, 64] and Lipinski’s five principles, with the results presented in Table 3.

**Table 3:** Drug-Likeness properties of compounds **2**–**5** and **8**–**11**

Compound	MW	nRot	HBA	HBD	TPSA	MLog P	Lipinski rule violation	Synthetic accessibility
**2**	317.36	5	4	2	88.58	1.13	0	2.75
**3**	413.88	6	4	2	88.58	2.86	0	3.10
**4**	409.46	7	5	2	97.81	2.06	0	3.13
**5**	393.46	6	4	2	88.58	2.60	0	3.12
**8**8	414.48	5	3	1	92.00	3.44	0	3.71
**9**	448.92	5	3	1	92.00	3.91	0	3.68
**10**	265.27	2	3	1	63.82	2.31	0	2.55
**11**	387.36	4	5	0	70.03	3.90	0	3.19

**Fig. 8:**
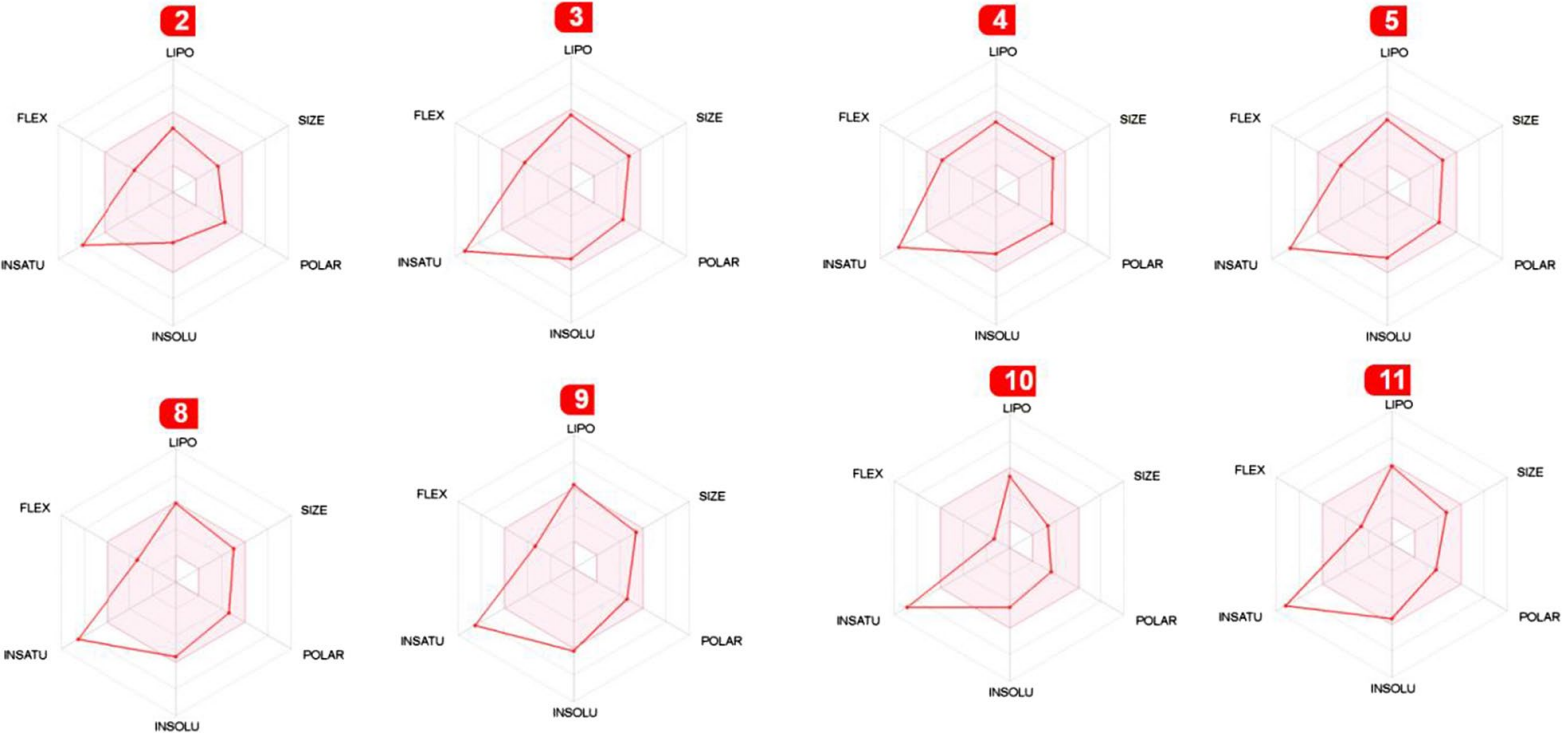
The bioavailability radars of **2–5** and **8–11** compounds

Our candidate compounds were quite successful in fitting the Lipinski rules with zero violations, as seen in the penultimate column of Table 3. When these physicochemical qualities are depicted visually as bioavailability radar, which considers six physicochemical properties: lipophilicity, size, polarity, solubility, flexibility, and saturation, the results in Table 3 become easier to understand. For each description, a physicochemical range is displayed as a pink band inside which the molecule’s radar plot must fall completely to be categorized as drug-like. The use of radars enables a quick evaluation of drug similarity guidelines. The red line of the researched molecule must be completely contained inside the pink zone to be classified as drug-like; any deviation outside the pink zone indicates a negative physicochemical trait [44, 46]. Figure 8 depicts the results of this study. When looking carefully at Fig. 8, it is seen that although the INSATU value in all molecules exceeds the pink region, other parameters remain within the region.

**Fig. 9:**
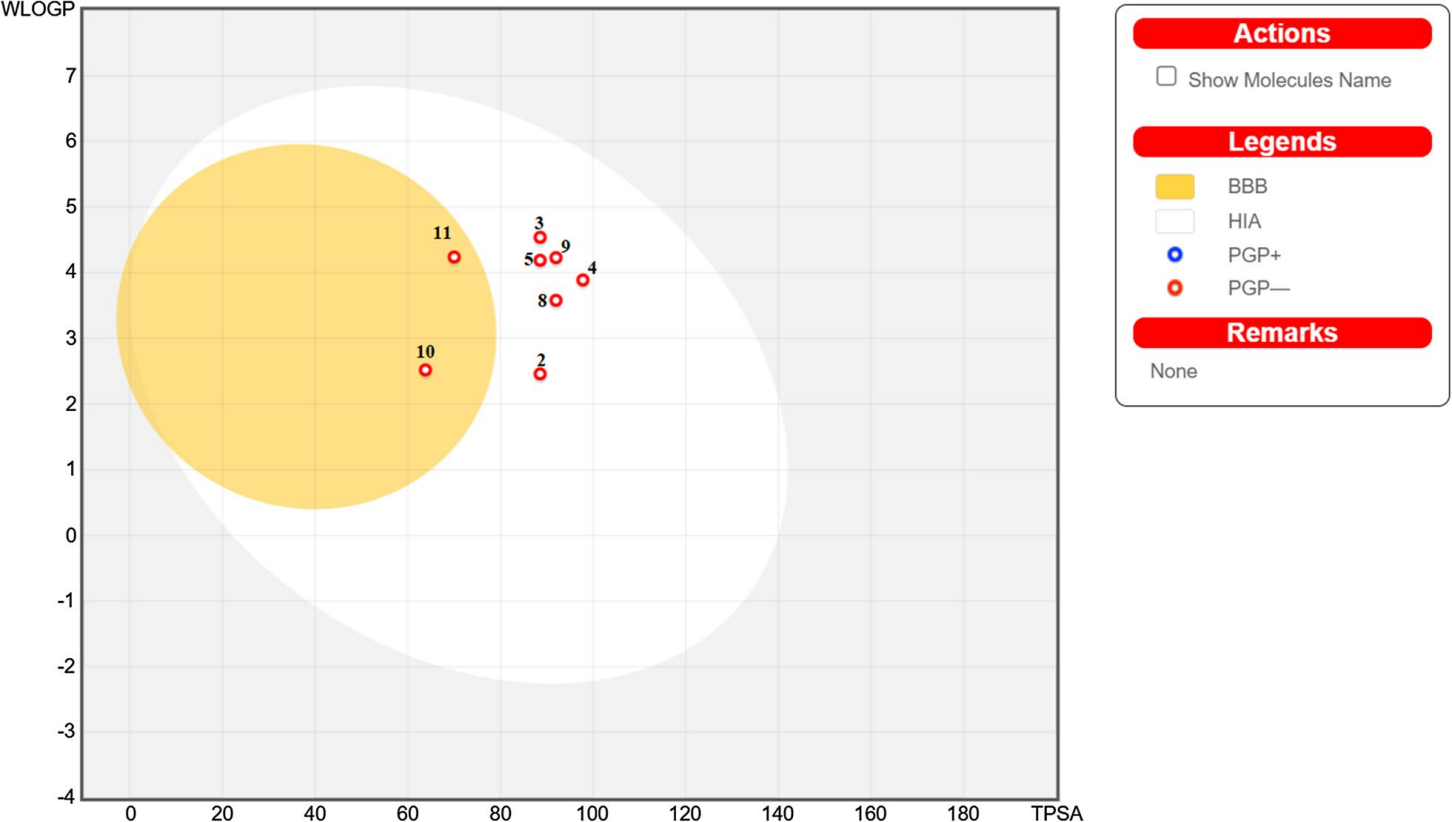
The boiled-egg plots of compounds **2**–**5** and **8**–**11**

Additionally, the synthetic accessibility values of the **2–5** and **8–11** compounds were determined as 2.75, 3.10, 3.13, 3.12, 3.71, 3.68, 2.55, and 3.19, respectively. Human intestinal absorption (HIA) and blood-brain barrier (BBB) access were predicted using the drugs’ WLogP and TPSA values (Fig. 9). The boiled-egg plot is divided into three parts: grey (no HIA or BBB access), white (HIA), and yellow yolk (BBB access). As seen in Fig. 8, all molecules are PGP (P-glycoprotein) negative, but six of the molecules are in the white region (2, 3, 4, 5, 8, and 9), on the other hand, molecules **10** and **11** are in the yellow region on the graph, that is, they (**10** and **11**) are located within the BBB barrier.

As a result, when the toxicity parameters of **2**–**5** and **8**–**11** molecules such as AMES toxicity, Max. tolerated dose (human), hERG I inhibitor, hERG II inhibitor, Oral Rat Acute Toxicity (LD50), Oral Rat Chronic Toxicity (LOAEL), Hepatotoxicity, skin sensitisation, Tetrahymena pyriformis (T. Pyriformis) toxicity, and Minnow toxicity values were examined using the pkCSM database [47] and the obtained values were given in Table 4.

**Table 4:** Toxicity scores of compounds **2–5** and **8–11**

Parameters	2	3	4	5	8	9	10	11
AMES toxicity	Yes	Yes	Yes	Yes	Yes	Yes	Yes	Yes
Max. tolerated dose (human)	0.373	0.244	0.214	0.237	0.421	0.331	0.063	0.505
hERG I inhibitor	No	No	No	No	No	No	No	No
hERG II inhibitor	Yes	Yes	Yes	Yes	Yes	Yes	Yes	Yes
Oral Rat Acute Toxicity (LD_50_ )	2.392	2.053	2.024	2.038	2.158	2.242	2.158	2.453
Oral Rat Chronic Toxicity (LOAEL)	1.75	1.655	1.358	1.608	1.634	1.467	1.081	0.224
Hepatotoxicity	Yes	Yes	Yes	Yes	Yes	Yes	No	Yes
Skin Sensitisation	No	No	No	No	No	No	No	No
*Tetrahymena pyriformis* toxiciity	0.638	0.323	0.306	0.327	0.34	0.335	0.447	0.288
Minnow toxicity	0.389	− 0.142	− 0.078	0.076	− 2.991	− 3.226	2.08	− 5.514

Positive toxicity levels for AMES were found, showing that the compounds are mutagenic and consequently carcinogenic. Max. tolerated dose (human) values were found as 0.373, 0.244, 0.214, 0.237, 0.421, 0.331, 0.063, and 0.505 for **2**–**5** and **8**–**11** molecules in terms of log mg/kg/day, respectively. For all compounds, hERG II inhibitors were proven to be YES, while hERG I inhibitors were determined to be NO. In terms of mol/kg, the oral rat acute toxicity (LD50) values were determined as 2.392, 2.053, 2.024, 2.038, 2.158, 2.242, 2.158, and 2.453 for 2–5 and 8–11 molecules, respectively. Oral Rat Chronic Toxicity (LOAEL) values were detected as 1.75, 1.655, 1.358, 1.608, 1.634, 1.467, 1.081, 0.224 for 2–5 and 8–11 molecules in terms of log mg/kg_bw/day, respectively. Hepatotoxicity values were found to be yes, however, molecule 10 was found to be no, and Skin Sensitisation descriptions for all compounds were found to be no. Toxicity values for T. Pyriformis were found to be 0.638, 0.306, 0.327, 0.340, 0.335, 0.447, and 0.288 in log ug/L, respectively; while minnow toxicity values were found to be 0.389, − 0.142, − 0.078, 0.076, − 2.991, − 3.226, 2.08, and − 5.514 in log mM for molecules 2–5 and 8–11, respectively.

#### Molecular dynamics simulations

The goal of the molecular dynamic (MD) simulation study was to determine the stability of ligand-protein complexes over time. The process of ligand fitting into the protein binding pocket changes the conformation of both the ligand and the protein backbones. These structural dynamics were simulated, and various trajectories were generated and analyzed to obtain specific parameters such as the root mean square deviation (RMSD) plot, which is used to evaluate the compactness and stability of the entire ligand-target enzyme complex [56]. The lower the RMSD value, the lower the structural fluctuations, and an RMSD value less than 3 nm indicates that the ligand is optimally fitted and docked within the binding pocket, resulting in a highly stable ligand-protein complex. The binding pocket XDH/PDB (3UNI) was selected for this experiment because the binding energy were − 10.1 and − 9.9 Kcal/mol for the most active compounds 9 & 10 respectively, in comparison with − 10.6 and − 8.3 Kcal/mol for PPARG/PDB (3VSO) binding pocket.

**Fig. 10:**
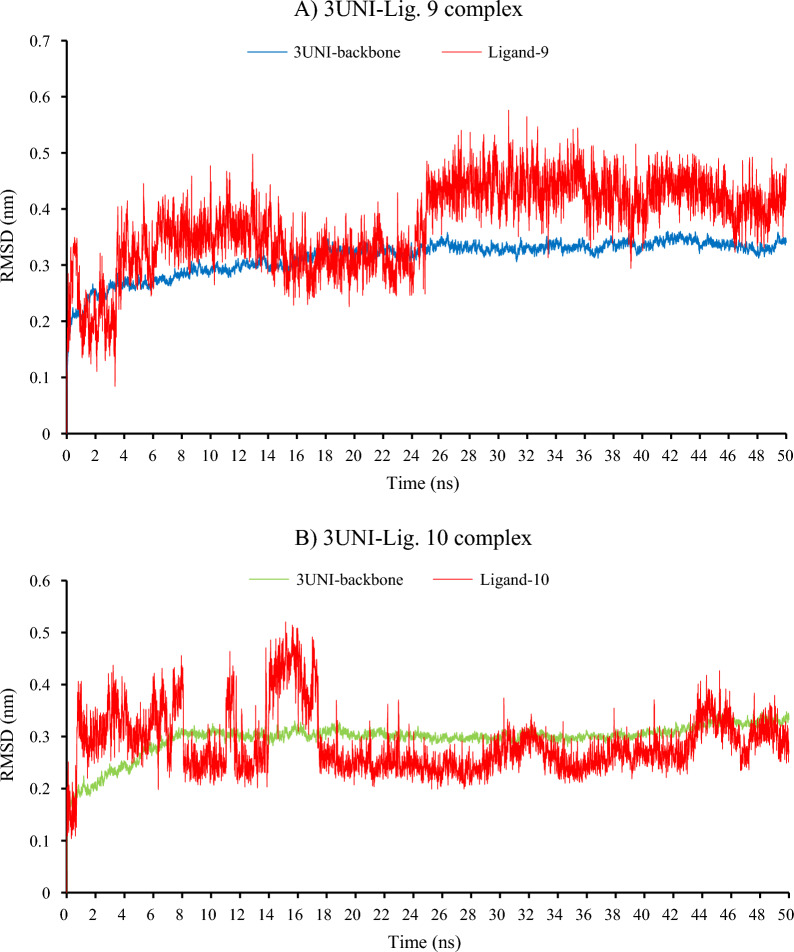
The RMSD/time plots presenting the molecular dynamics simulation trajectory of **A** 3UNI-Ligand 9 complex, **B** 3UNI-Ligand 10 complex, the red color represents the 3UNI protein backbone, the blue and green colors represent the ligands 9, and 10 respectively

As presented in Fig.10, the protein (3UNI) backbone upon **9** and **10** ligands fitting shows trivial fluctuations (within 0.5 nm), and the simulation trajectories reached the plateau of equilibrium at approximately 16 ns in both complexes. Concerning ligand trajectories, the presented ligand-RMSD plots indicate that the **10** candidate has a higher structural dynamicity than the **9** structure within the binding pocket. Both compounds 9 and 10 show low structural fluctuations and their plateau of equilibrium started at approximately 10 nm and retain the stable state. Both candidates show ideal simulation trajectories and minimal structural fluctuations (do not exceed 0.55 nm) which emphasize their high stability within the binding site.

## Conclusion

Eight molecules (**2–5** and **8–11**) were synthesized in the present investigation, which showed various degrees of biological activity. Indeed, **10** and **11** compounds were found to have a stronger antiproliferative effect than **2**–**5** compounds against HepG2, HeLa, and MCF7 cancer cell lines. Moreover, compound **9** showed strong activity against the HeLa cancer cell line. Moreover, all of the synthesized compounds, except for compound **5**, showed moderate antiproliferative activities against CaCo-2. In addition, compound **9** showed the most potent anti-fibrotic activity. In addition, **4** and **9** compounds showed potent antioxidant activity against DPPH. The validation rules and binding active pockets of molecules **2–5** and **8–11**, which are categorized as XDH/PDB: 3UNI and PPARG/PDB: 3VSO inhibitors, were examined in this research using the Autodock Vina program. When the docking results were examined, it was observed that molecule **3** had greater binding energy (− 11.3 kcal/mol) than the other compounds against the PDB: 3UNI receptor. Molecule**-11**, on the other hand, displayed a larger binding energy (− 11.1 kcal/mol) against the PDB: 3VSO receptor than the other compounds. Additionally, the Autodock Vina molecular docking results were compared and supported with CB-Dock, and the results were determined to be consistent. All substances met the parameters of Lipinski’s drug-likeness recommendations, according to pharmacokinetic studies. As seen from the results, all molecules are PGP negative, but six of the molecules are in the white region. On the other hand, molecules **10** and **11** are located within the BBB barrier. As a result, the chemicals tested can be orally available drugs, and the theoretical data can be used to guide future experimental research based on the findings. Finally, the toxicity of compounds was explored using molecular docking and pharmacokinetic outcomes, and the results were analyzed using pkCMS. These ligands could be used as a starting point for building more effective molecules, with various structural changes, based on the findings.

## Supplementary Information

**Additional file 1:** Contains chemical structures, LC-MS, NMR spectrums, and Docking Interactions of Compounds 2, 4, 5, 8, 9, 10, and 11.

## Data Availability

All data generated or analyzed during this study are included in this published article (and its additional files).
