# Supplementary material for: Design, synthesis, molecular docking and biological evaluation of new carbazole derivatives as anticancer, and antioxidant agents
Source: BMC Chem. 2023 Jun 16;17(1):60. doi: 10.1186/s13065-023-00961-y (PMC10276425; doi:10.1186/s13065-023-00961-y)
Supplement: Supplementary file 1 — Additional file 1: Contains chemical structures, LC-MS, NMR spectrums, and Docking Interactions of Compounds 2, 4, 5, 8, 9, 10, and 11. [file 13065_2023_961_MOESM1_ESM.docx]

Supplementary Information

**Design, Synthesis, Molecular docking and Biological Evaluation of New Carbazole Derivatives as Anticancer, and Antioxidant Agents**

**İrfan Çapan ^a,*^, Mohammed Hawash ^b,*^, Nidal Jaradat^b^, Yusuf Sert^c^, Refik Servi^d^, İrfan Koca^e^**

^1*^Gazi University, Technical Sciences Vocational College, Department of Material and Material Processing Technologies, 06560 Ankara, Türkiye.

^2^An-Najah National University, Faculty of Medicine and Health Sciences, Department of Pharmacy, Nablus 00970, Palestine.

^3^ Yozgat Bozok University, Sorgun Vocational School, Yozgat, Türkiye.

^4^Firat University, Faculty of Medicine, Department of Anatomy, Elazig, Türkiye

^5^Yozgat Bozok University, Department of Chemistry, Faculty of Art & Sciences, Yozgat, Türkiye.

^∗^Corresponding author: **İrfan Çapan**, Gazi University, Technical Sciences Vocational College, Department of Material and Material Processing Technologies, 06560 Ankara, Türkiye, E-mail: [irfancapan@gazi.edu.tr](mailto:irfancapan@gazi.edu.tr), and **Mohammed Hawash**, Department of Pharmacy, Faculty of Medicine and Health Sciences, An-Najah National University, Nablus, PALESTINE, orcid.org/0000-0001-5640-9700; Phone: +972569939939; Email: [mohawash@najah.edu](mailto:mohawash@najah.edu)

Table of Contents

[Spectrums of Compound 2 3](#_Toc128342954)

[Spectrums of Compound 3 5](#_Toc128342955)

[Spectrums of Compound 4 7](#_Toc128342956)

[Spectrums of Compound 5 9](#_Toc128342957)

[Spectrums of Compound 6 11](#_Toc128342958)

[Spectrums of Compound 7 13](#_Toc128342959)

[Spectrums of Compound 9 17](#_Toc128342960)

[Spectrums of Compound 10 19](#_Toc128342961)

[Spectrums of Compound 11 21](#_Toc128342962)

[Docking Interactions of Compounds 2, 4, 5, 8, 9, 10, and 11 23](#_Toc128342963)

# Spectrums of Compound 2


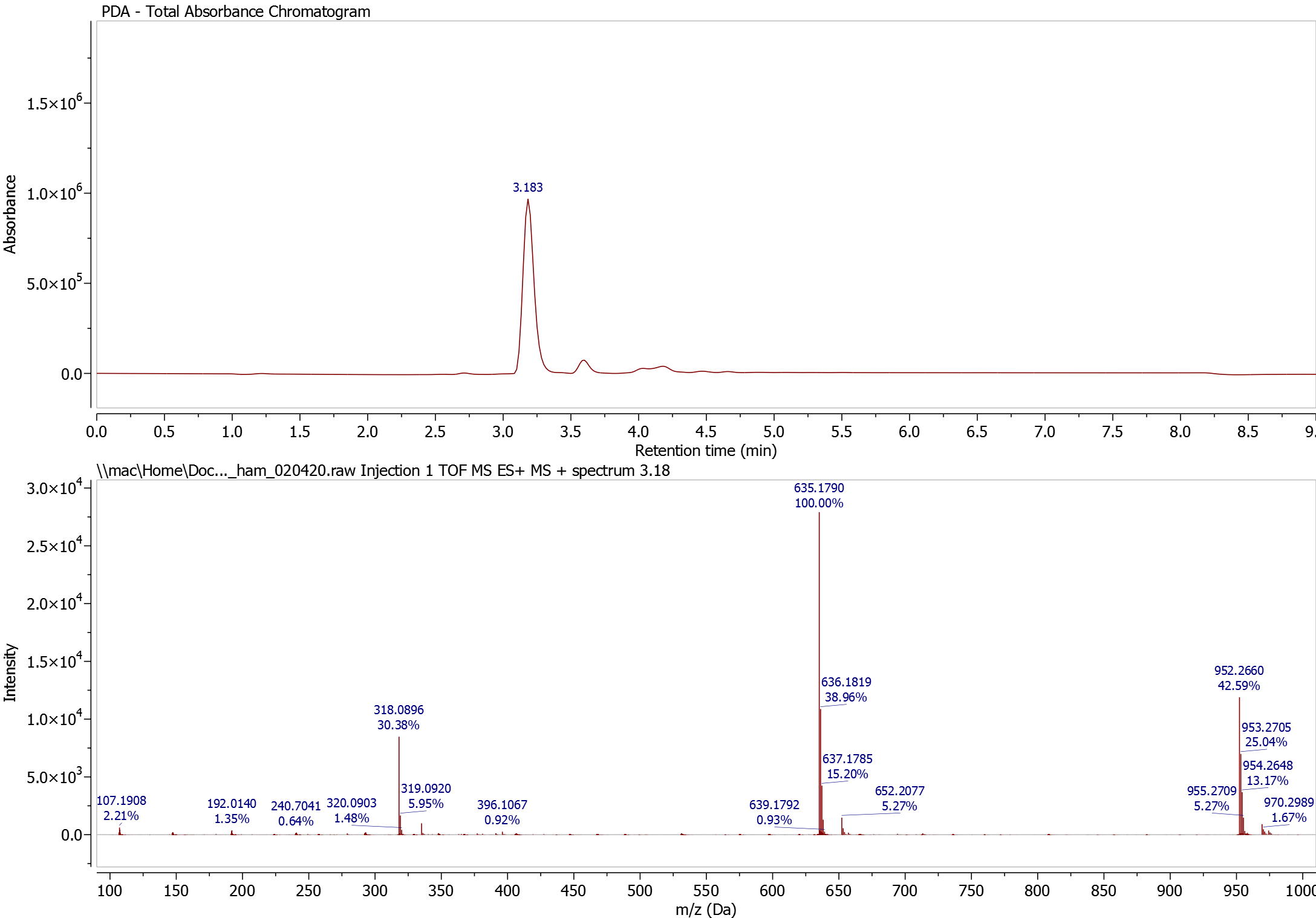


Figure S1. LC-MS Spectrum of Compound **2**


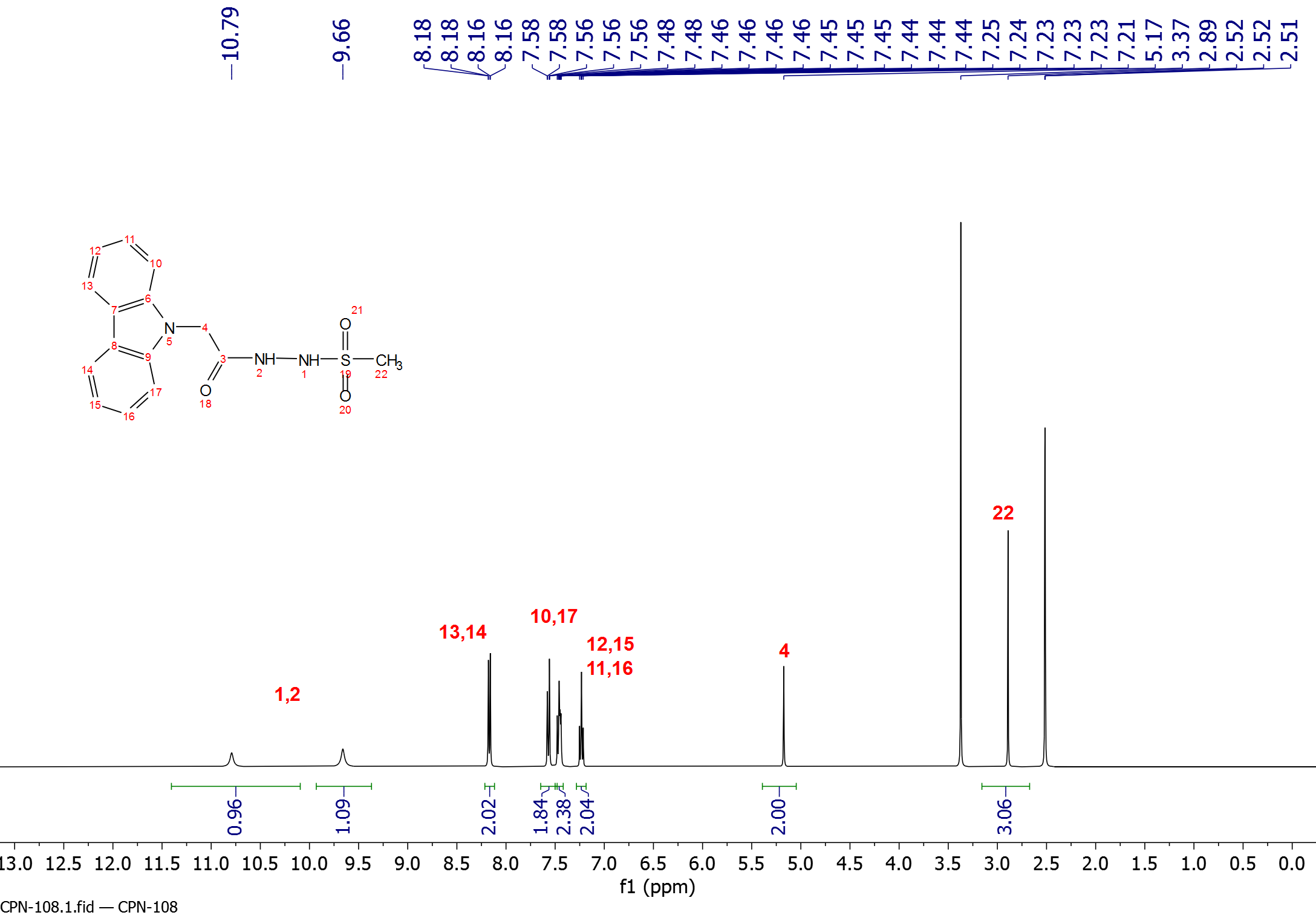

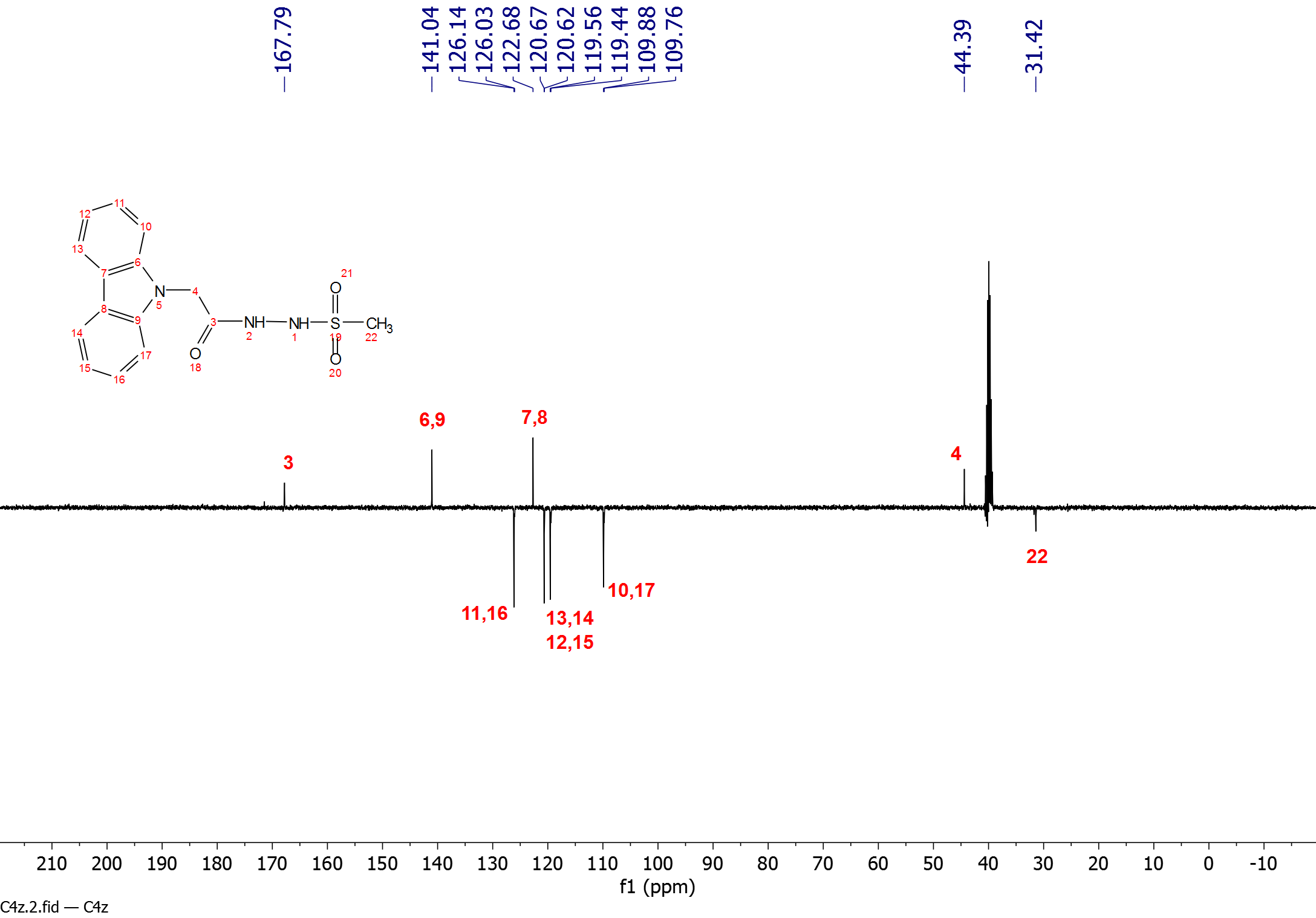
Figure S2. ^1^H-NMR Spectra of Compound **2**

Figure S3. ^13^C_APT_-NMR Spectra of Compound **2**

# Spectrums of Compound 3


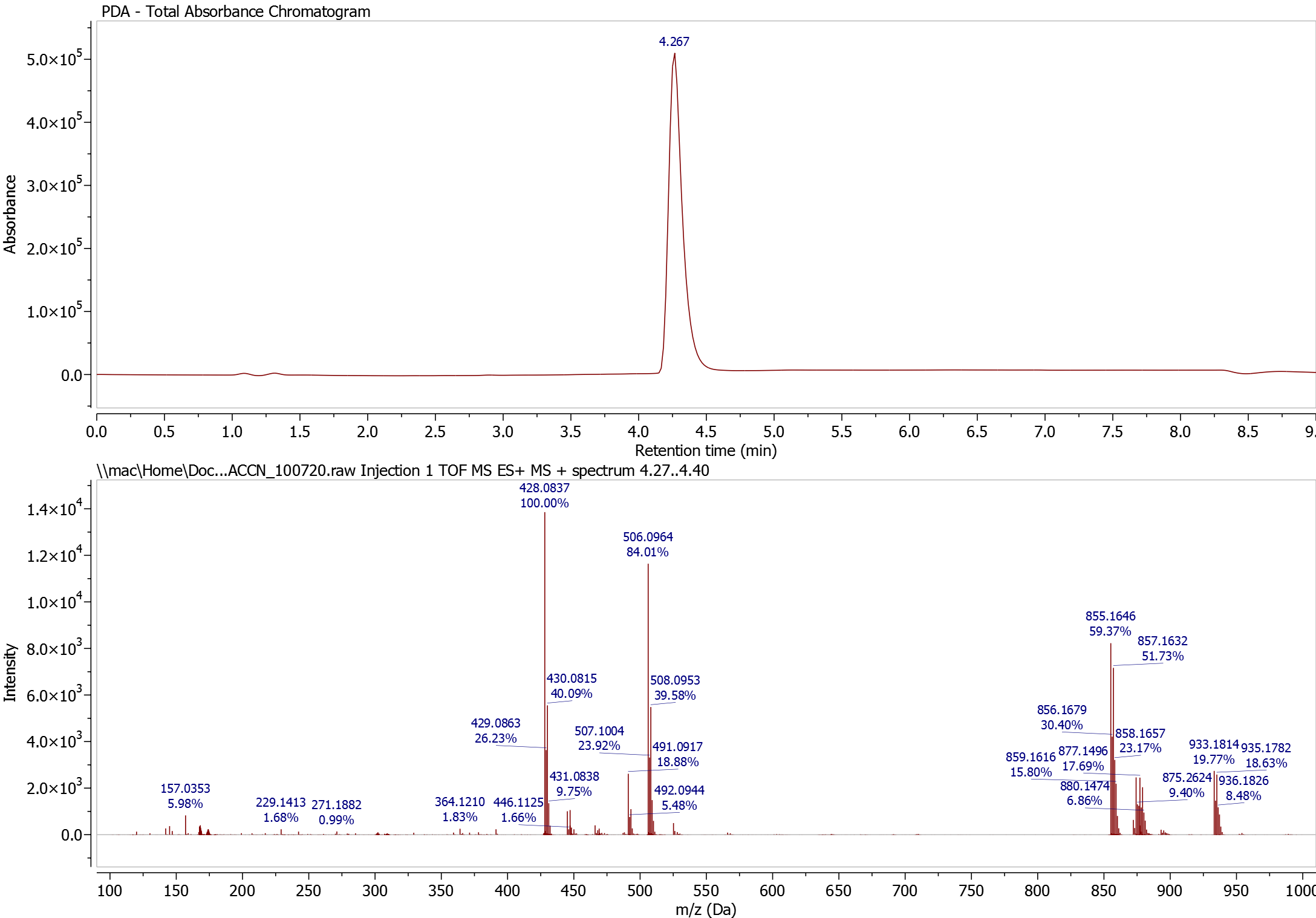


Figure S4. LC-MS Spectrum of Compound **3**


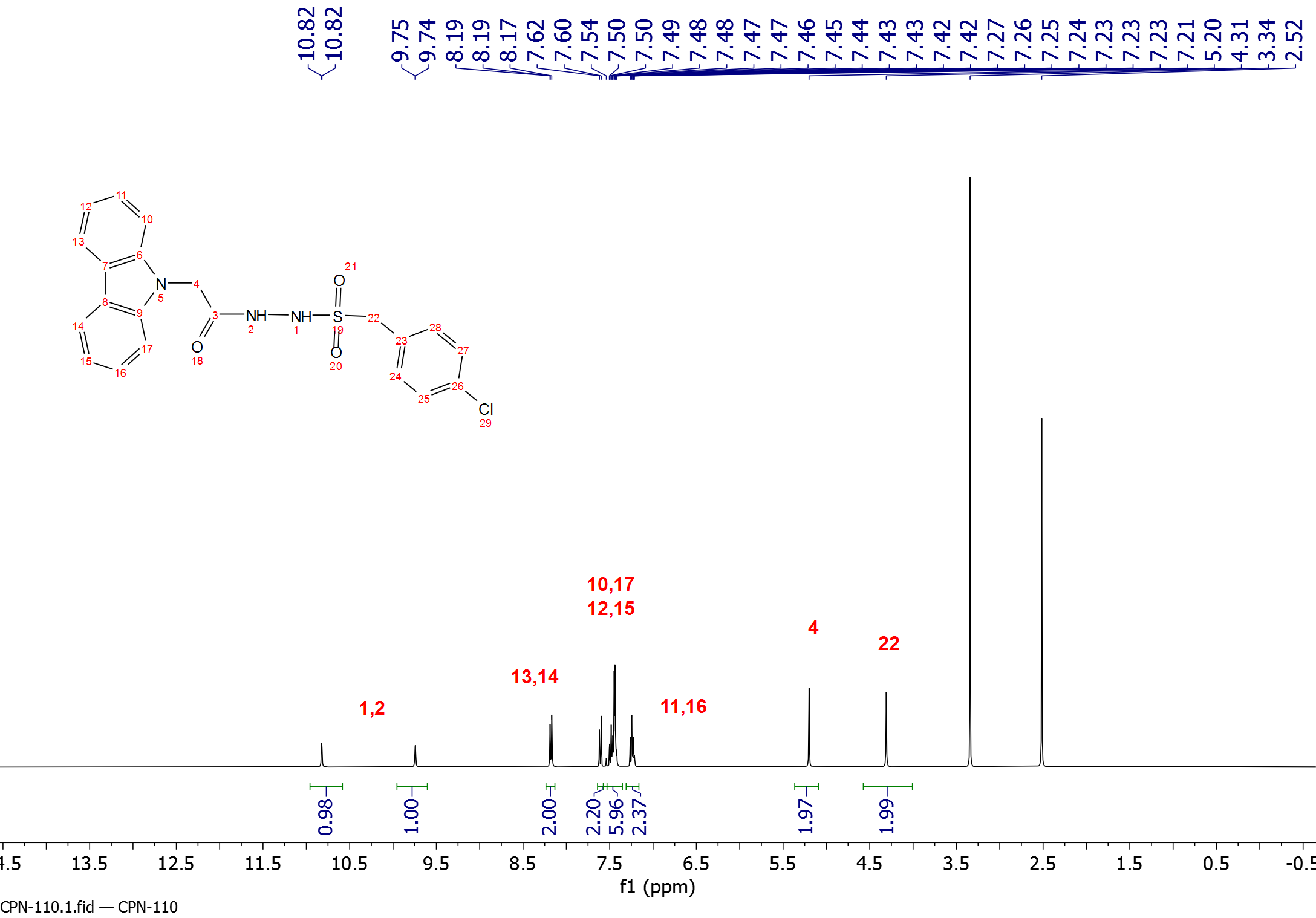
Figure S5. ^1^H-NMR Spectra of Compound **3**


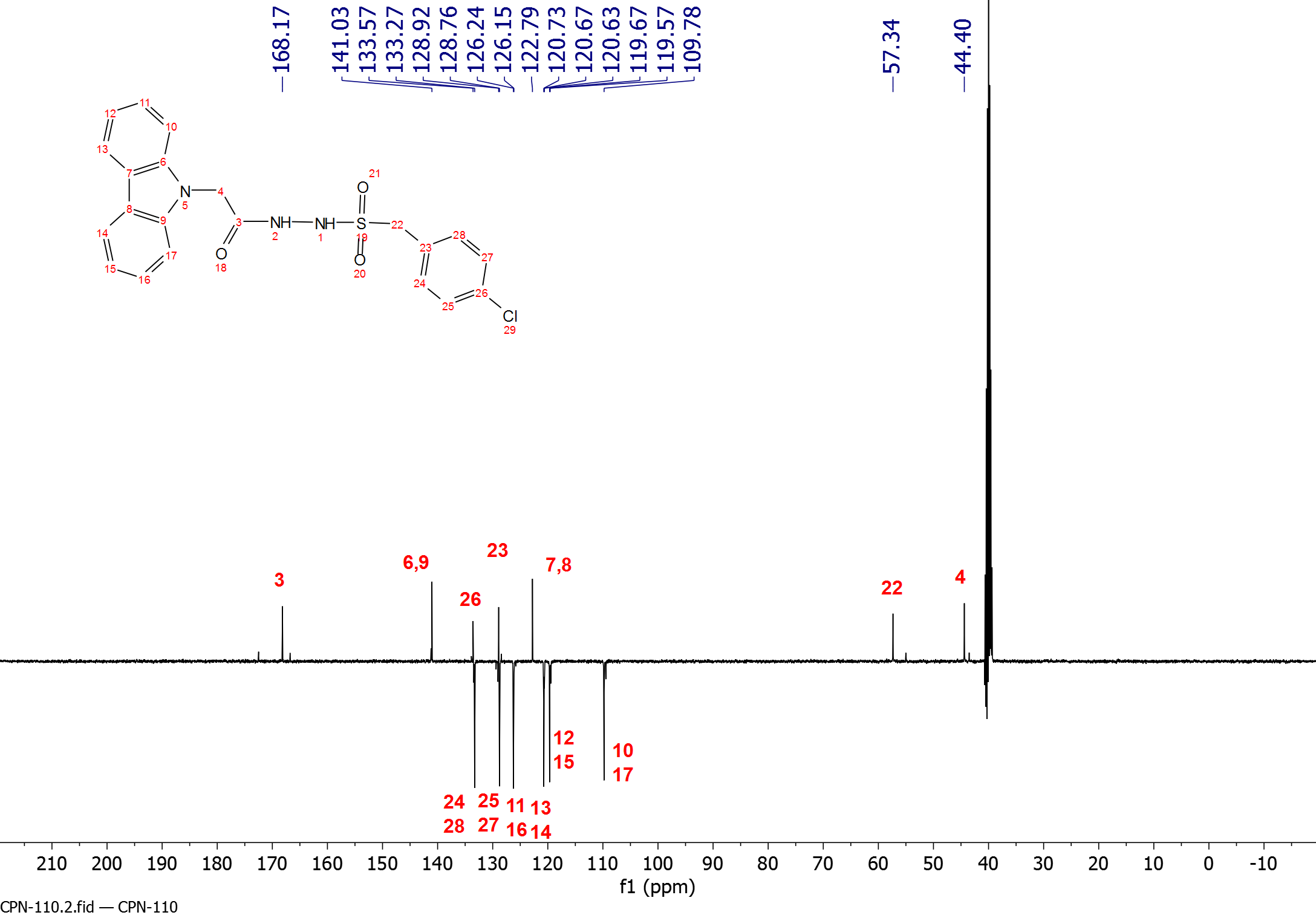


Figure S6. ^13^C_APT_-NMR Spectra of Compound **3**

# Spectrums of Compound 4


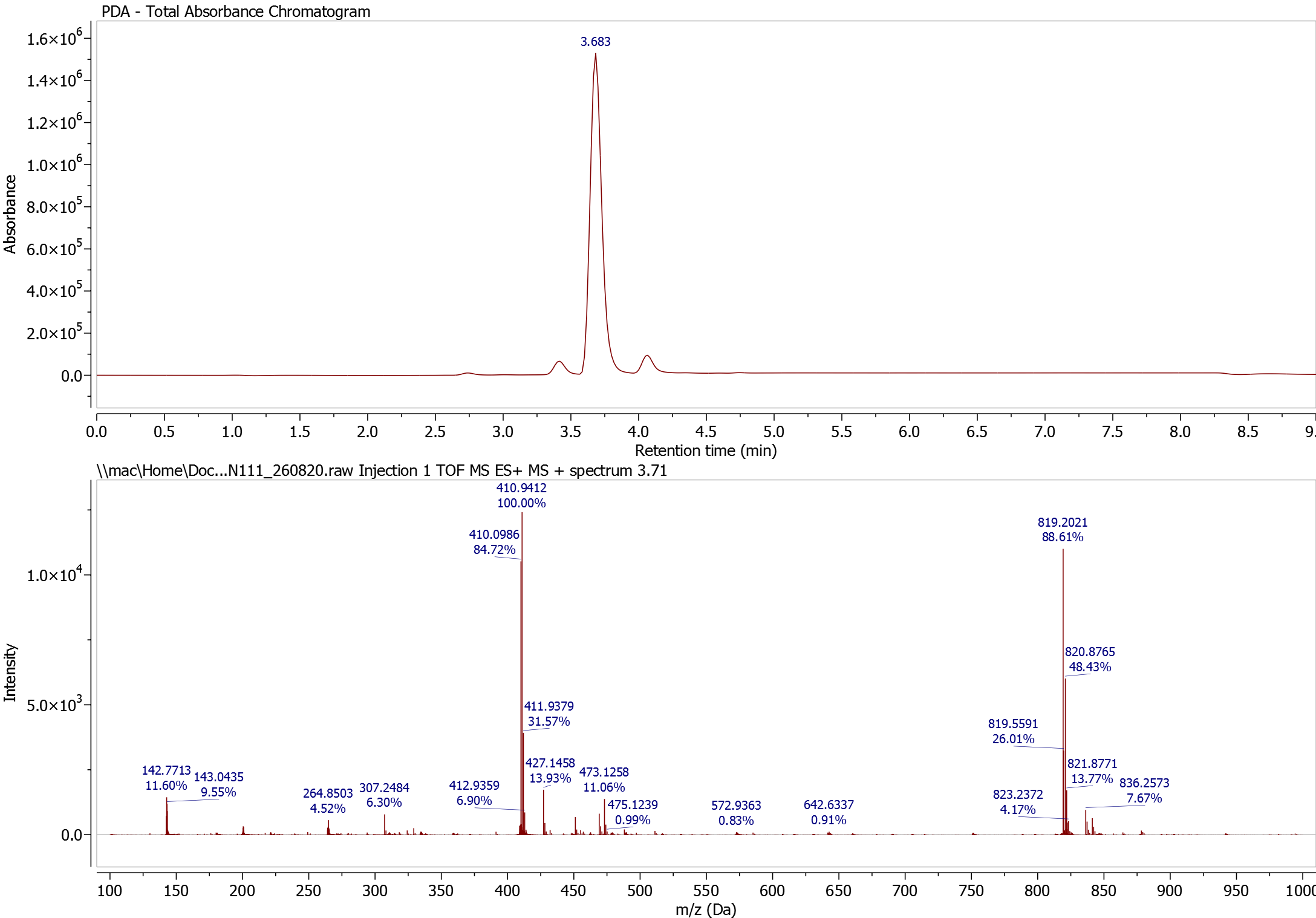


Figure S7. LC-MS Spectrum of Compound **4**


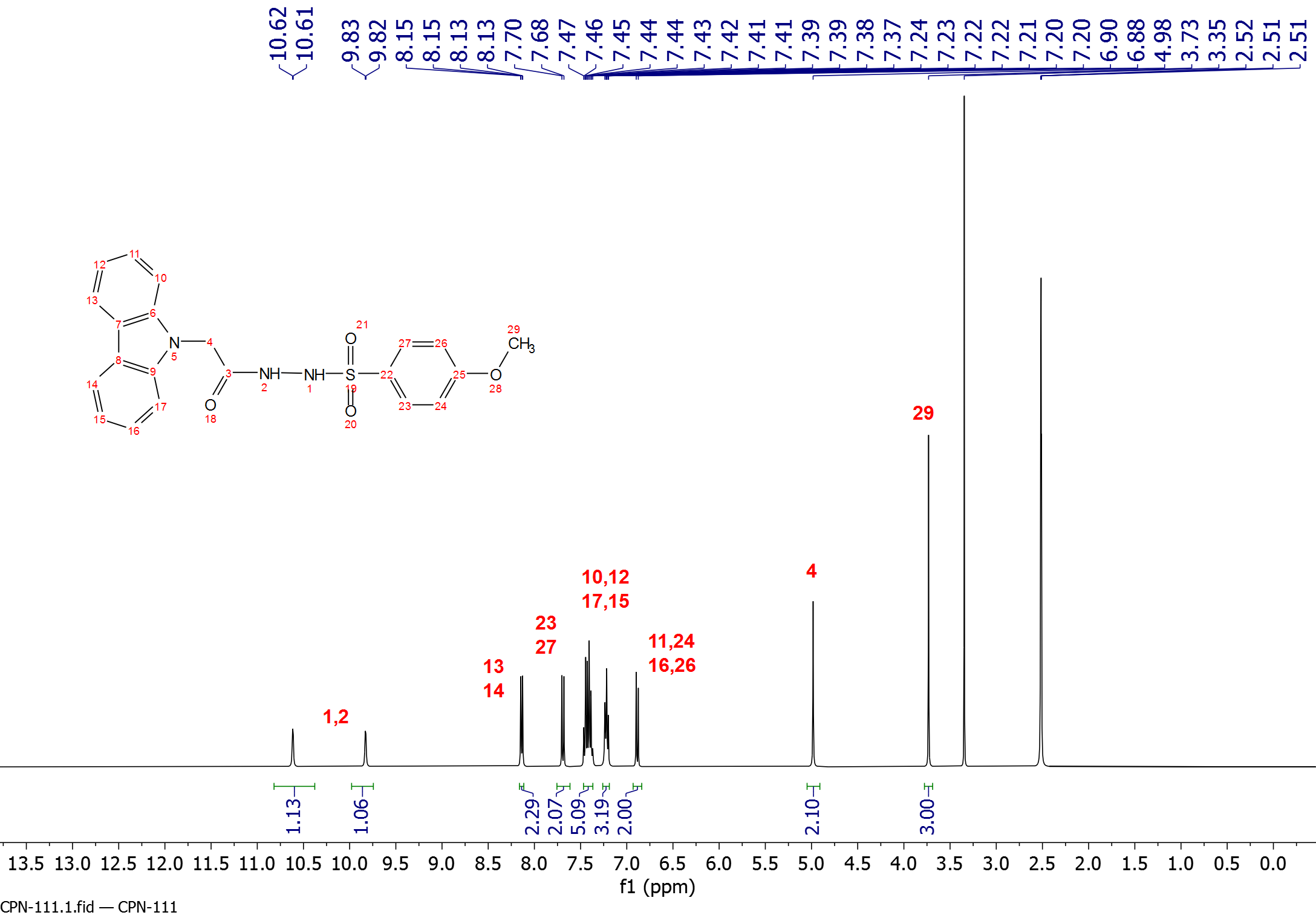
Figure S8. ^1^H-NMR Spectra of Compound **4**


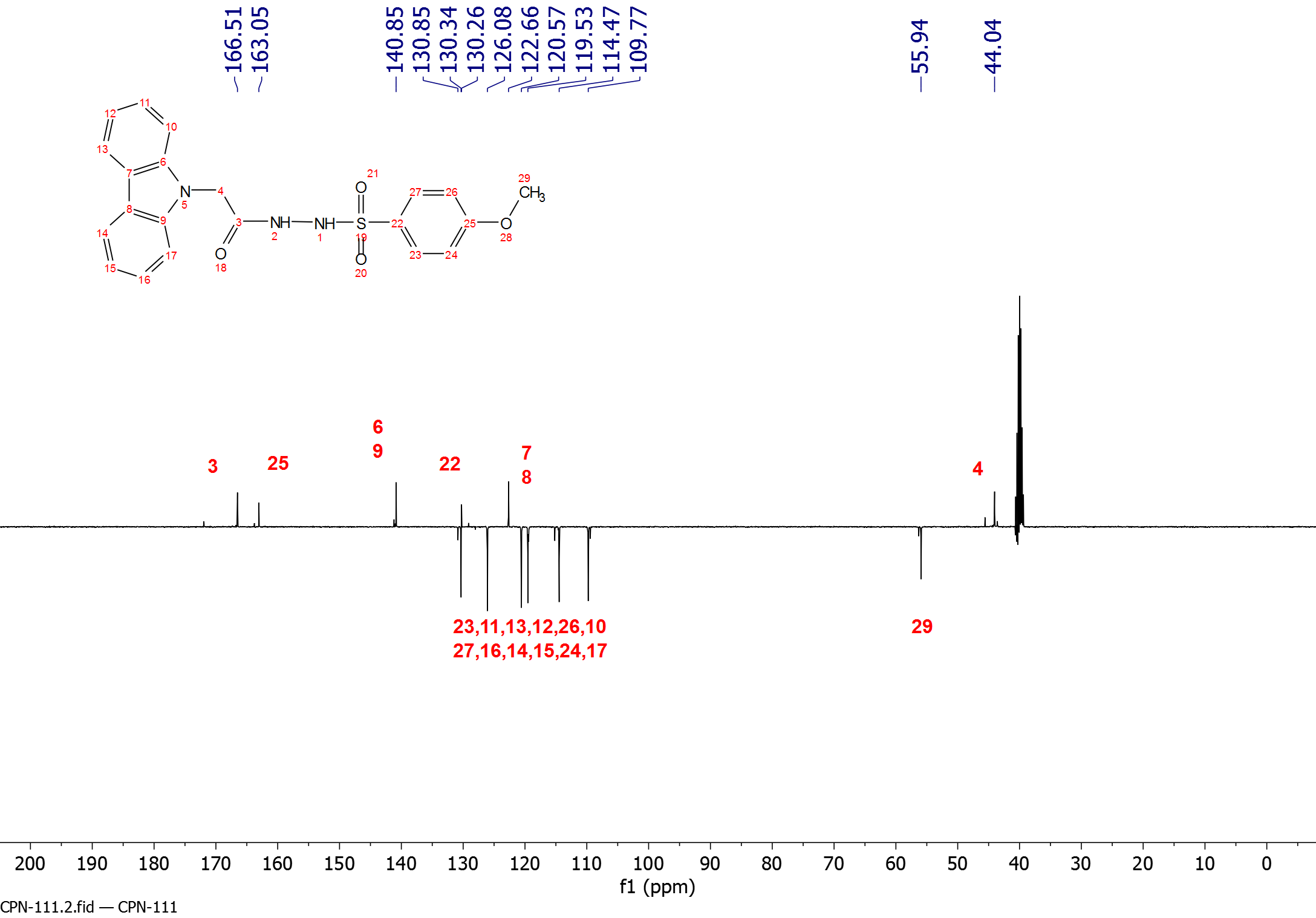


Figure S9. ^13^C_APT_-NMR Spectra of Compound **4**

# Spectrums of Compound 5


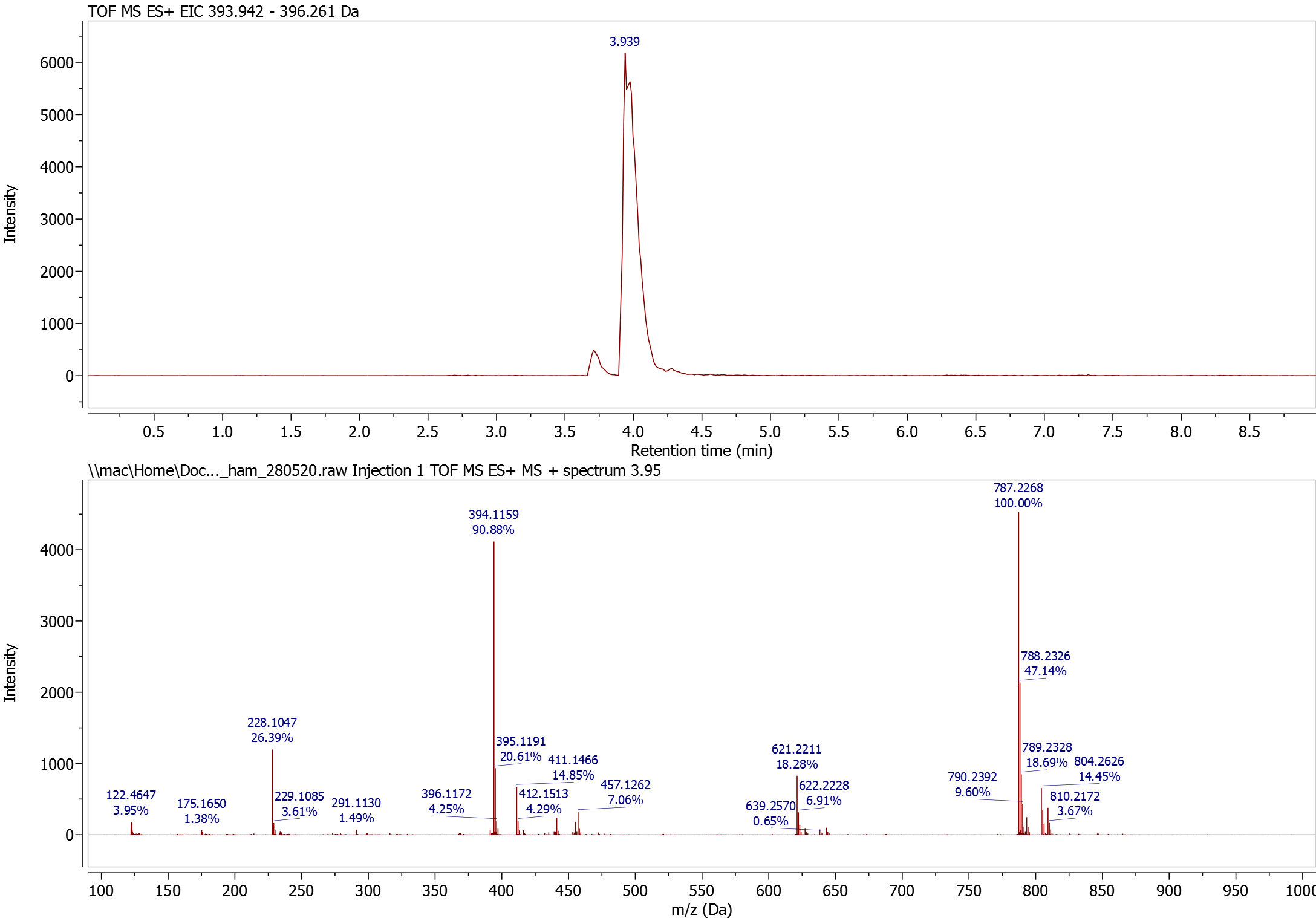


Figure S10. LC-MS Spectrum of Compound **5**


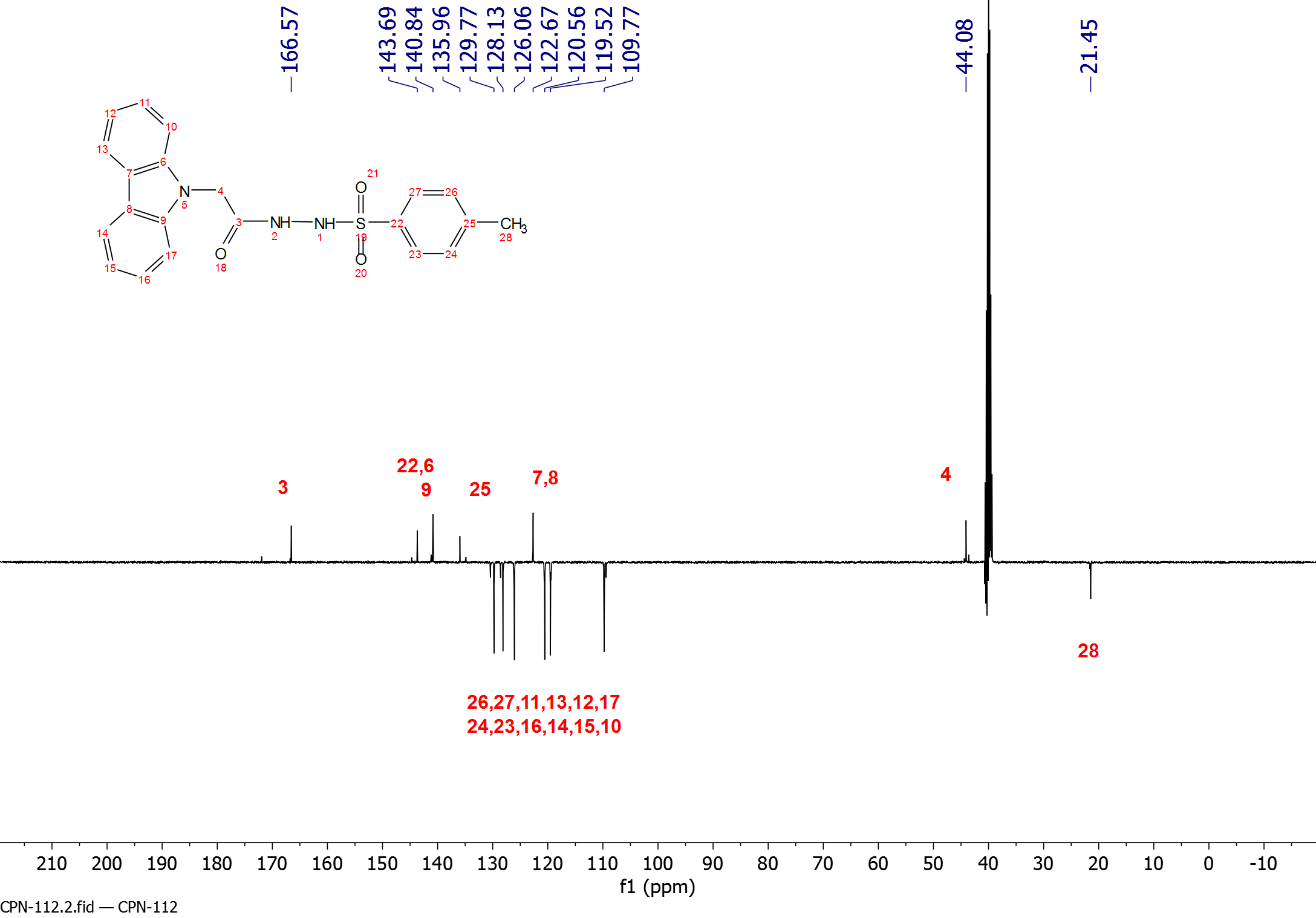

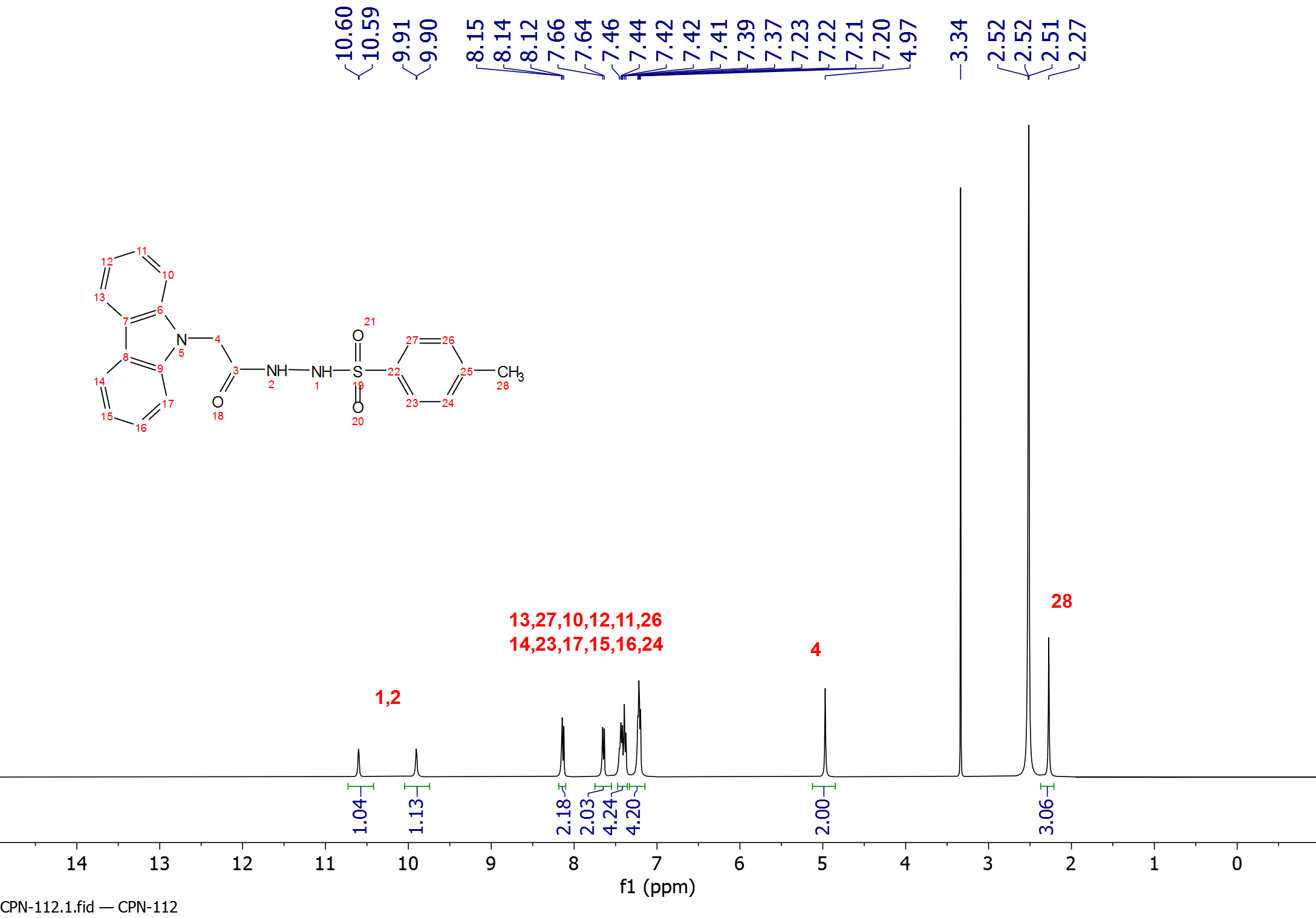
Figure S11. ^1^H-NMR Spectra of Compound **5**

Figure S13. ^13^C_APT_-NMR Spectra of Compound **5**

# Spectrums of Compound 6


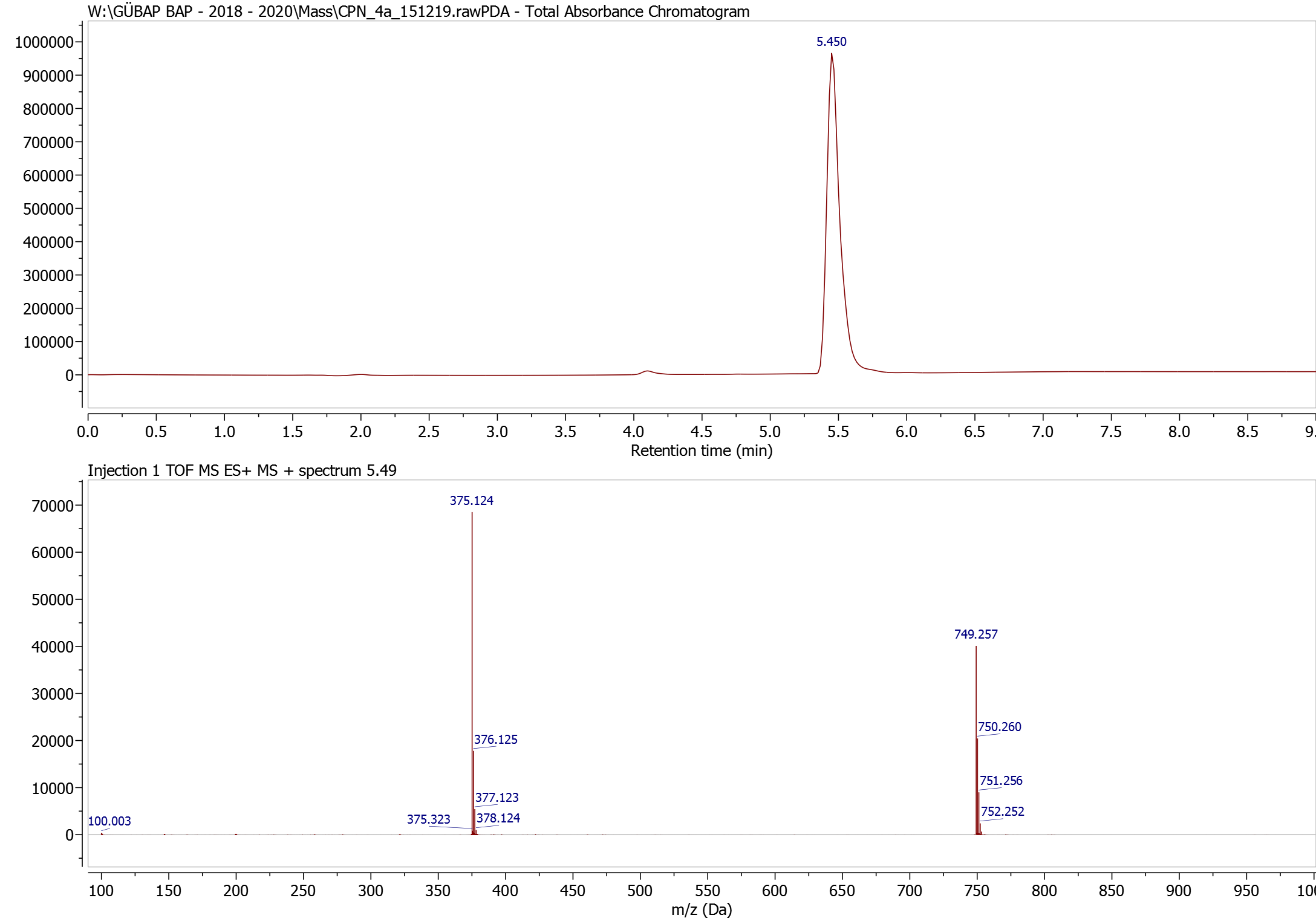


Figure S14. LC-MS Spectrum of Compound **6**


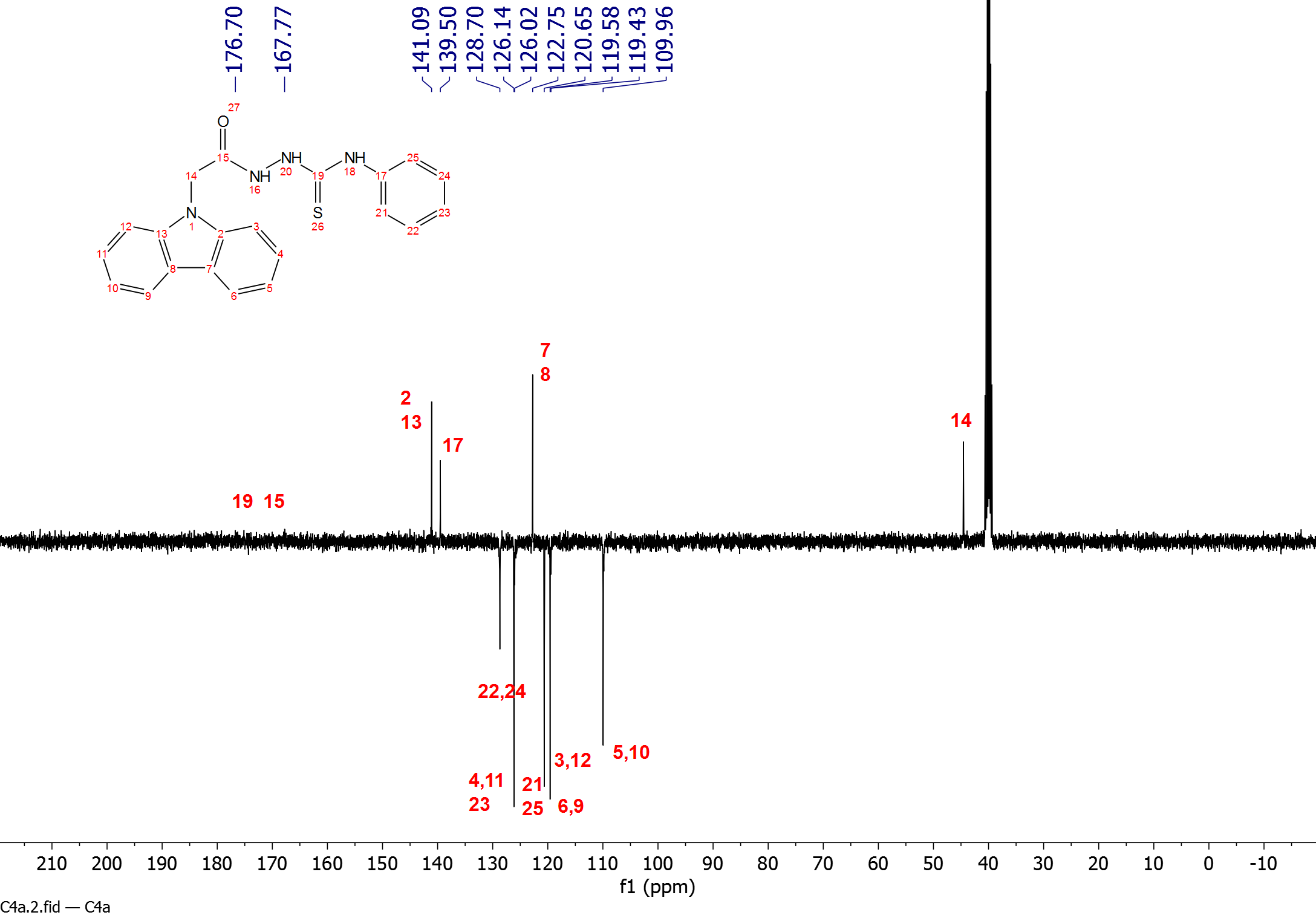

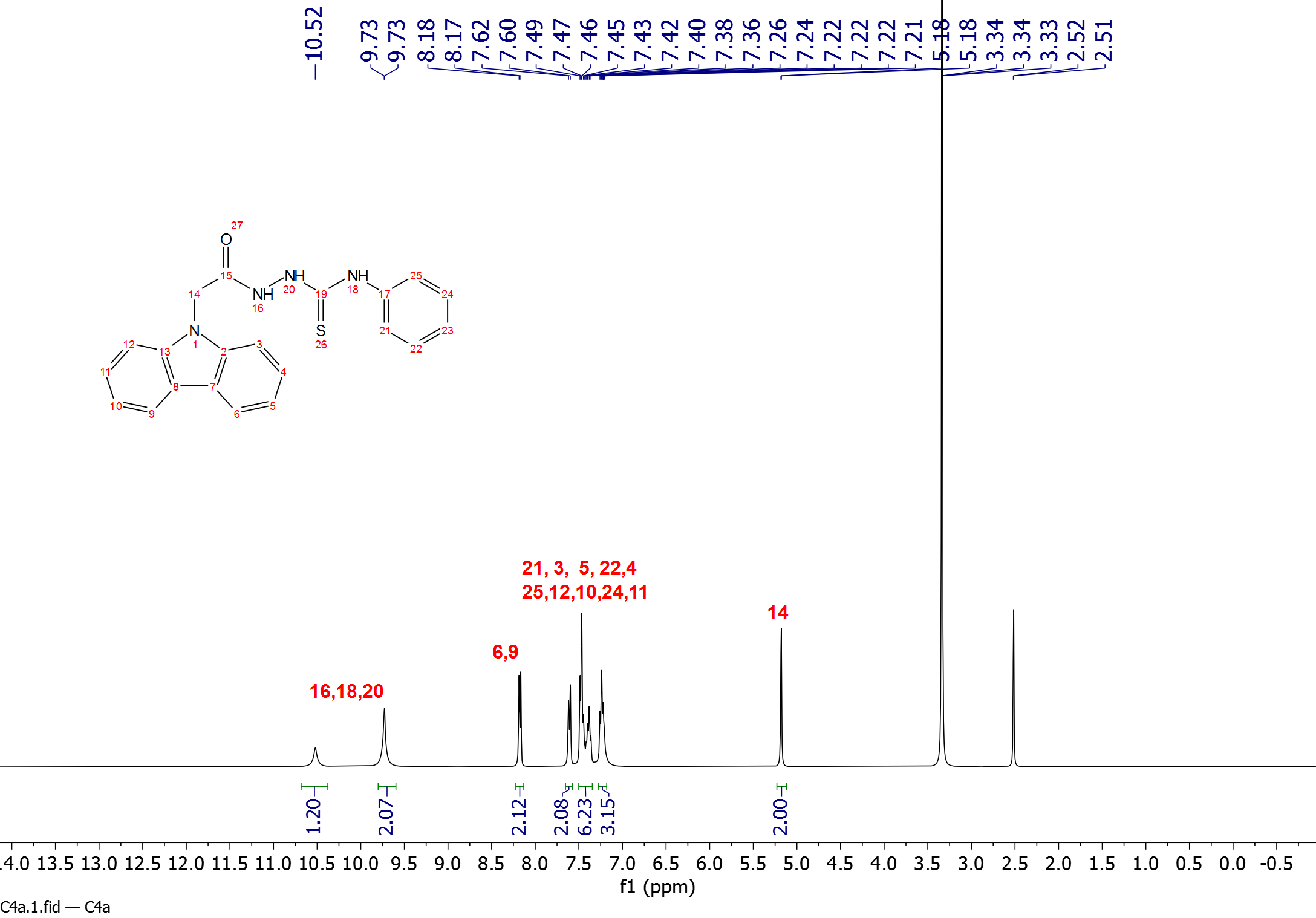
Figure S15. ^1^H-NMR Spectra of Compound **6**

Figure S16. ^13^C_APT_-NMR Spectra of Compound **6**

# Spectrums of Compound 7


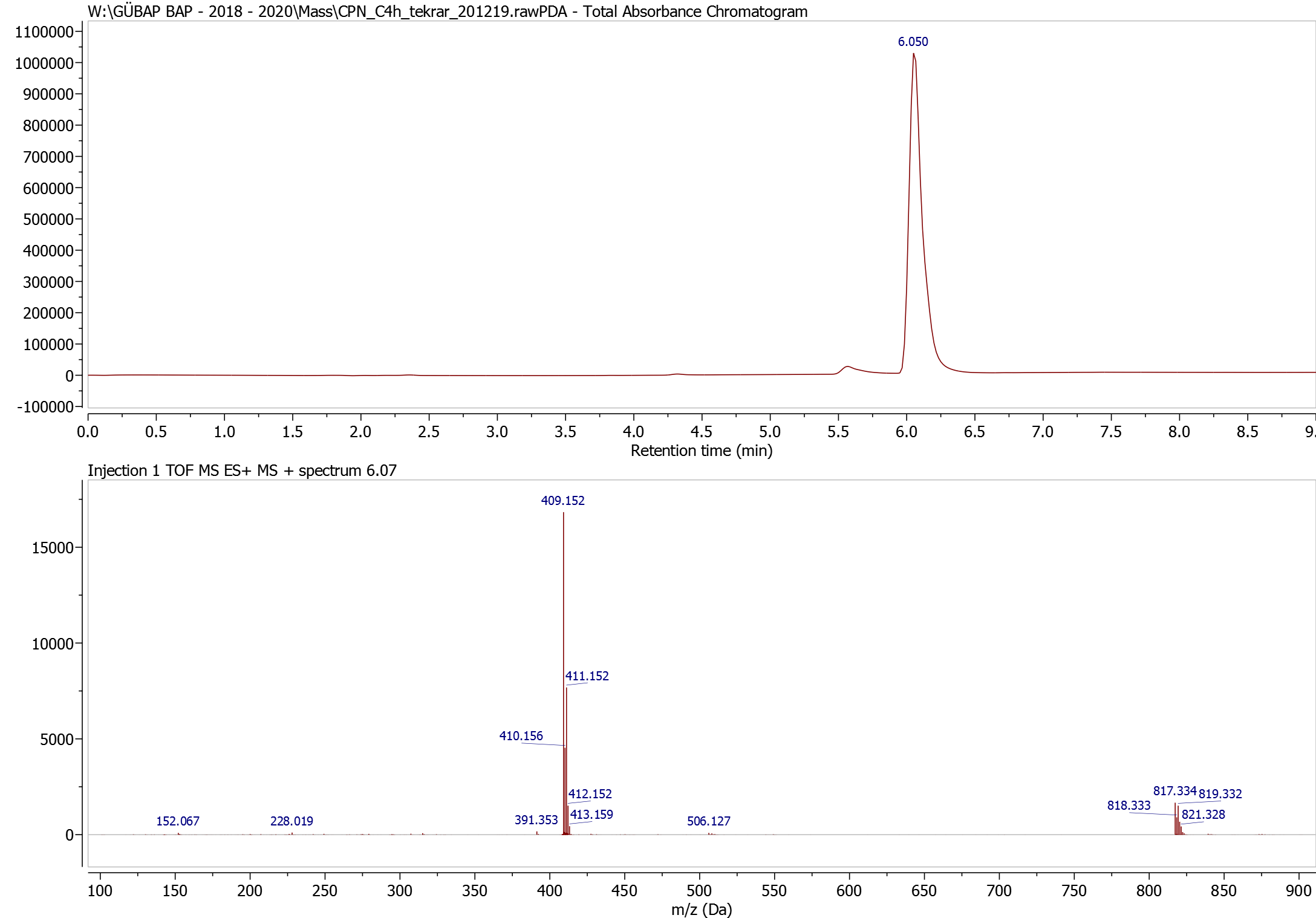


Figure S17. LC-MS Spectrum of Compound **7**


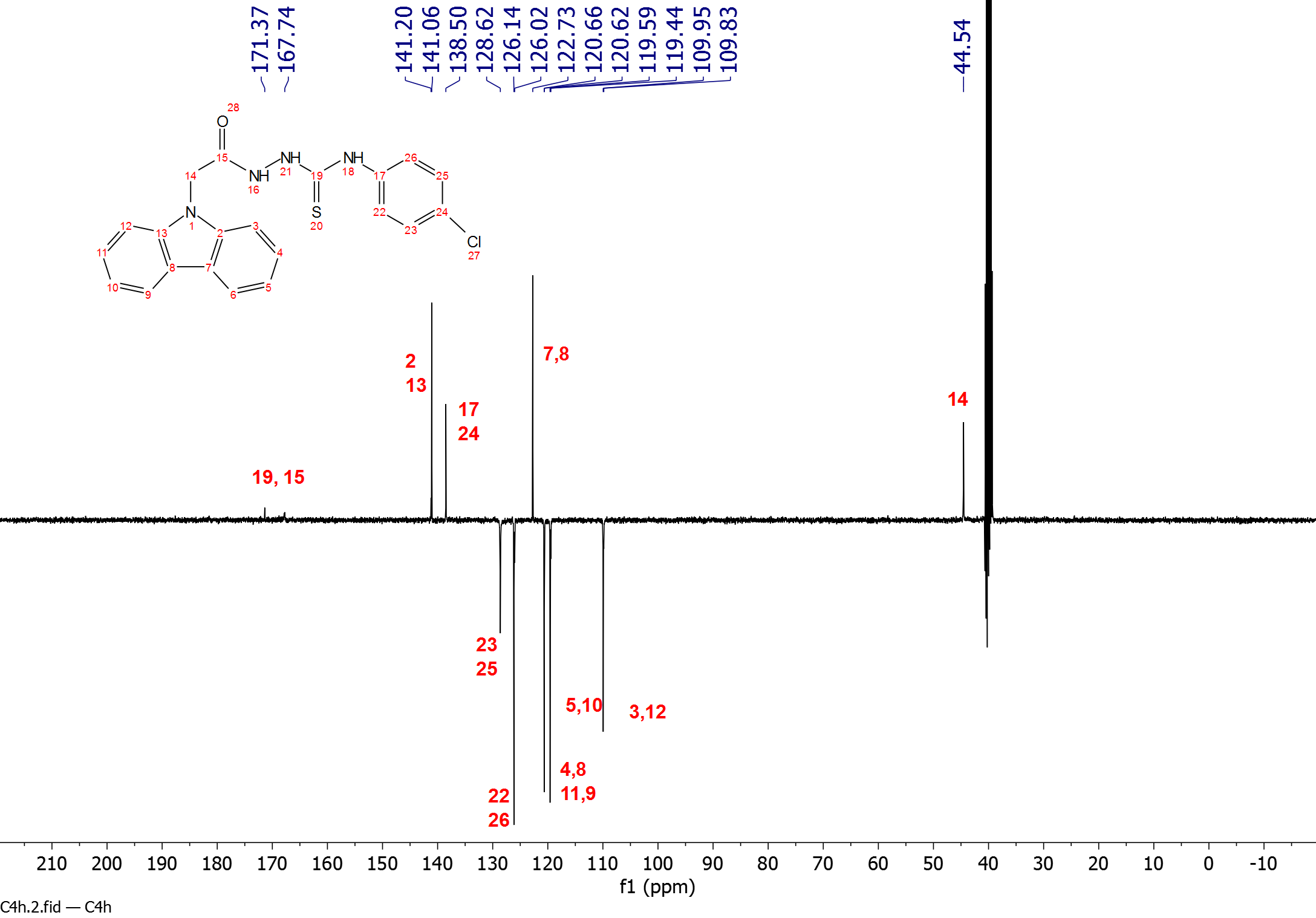

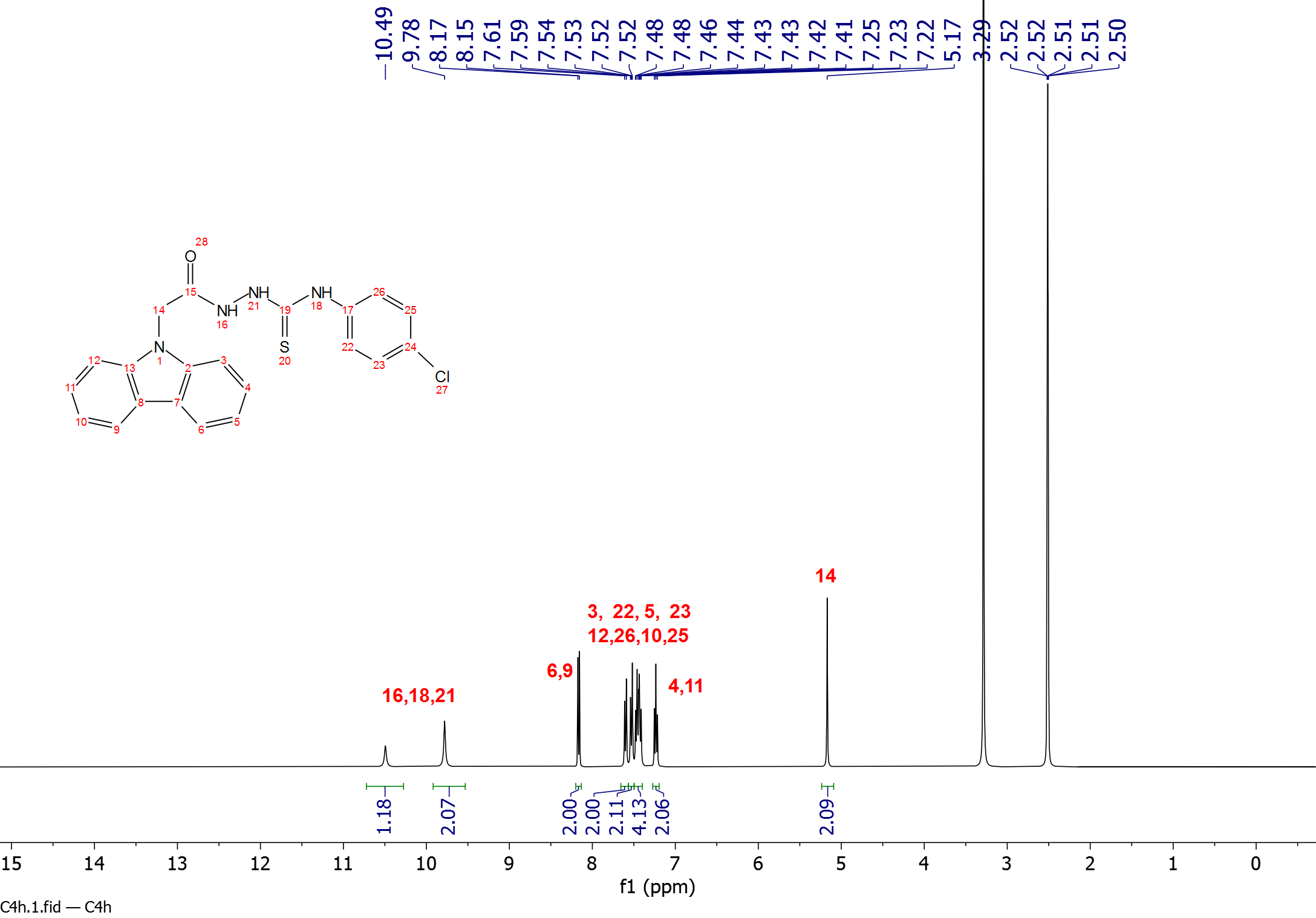
Figure S18. ^1^H-NMR Spectra of Compound **7**

Figure S19. ^13^C_APT_-NMR Spectra of Compound **7**

**Spectrums of Compound 8**


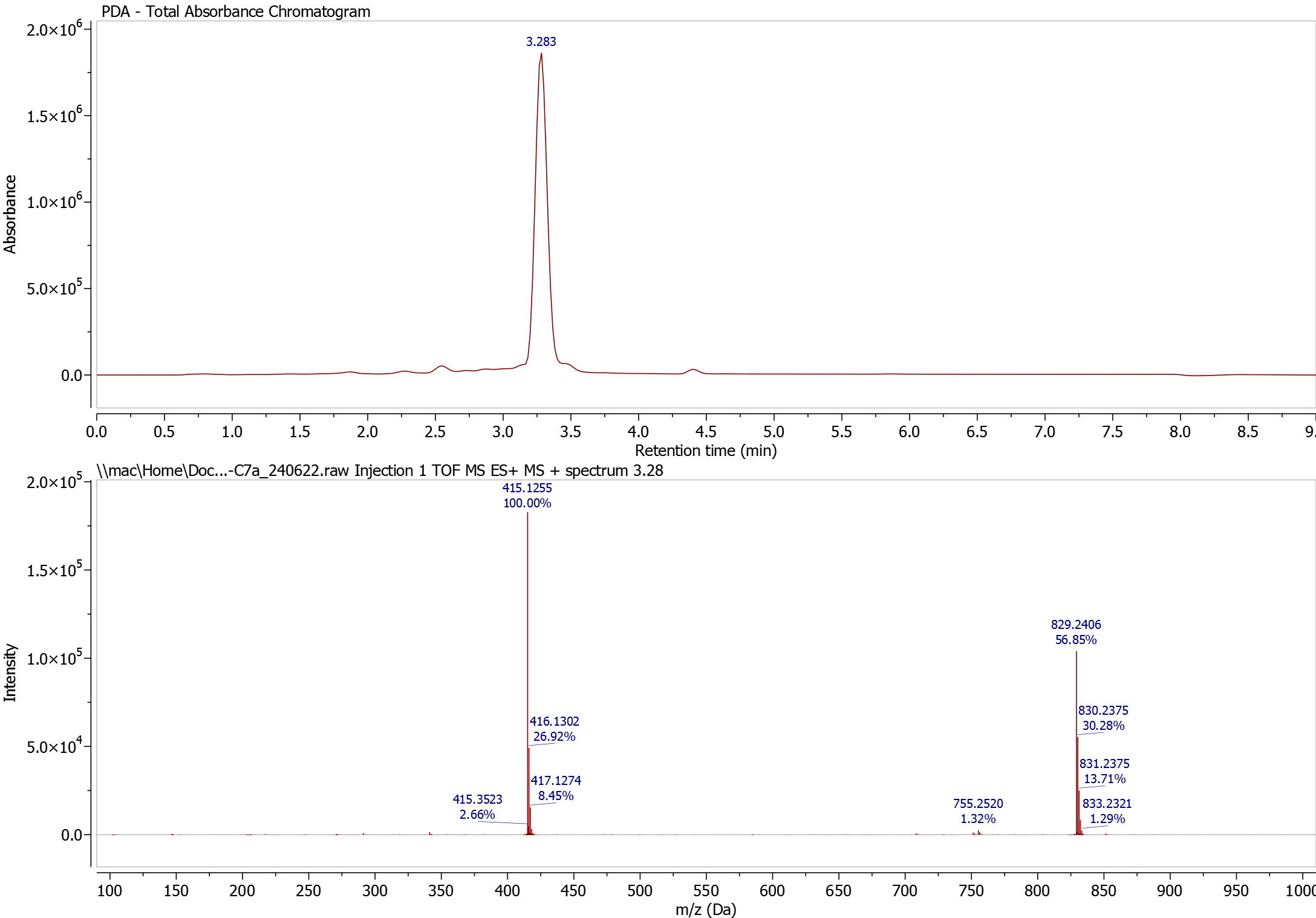


Figure S20. LC-MS Spectrum of Compound **8**


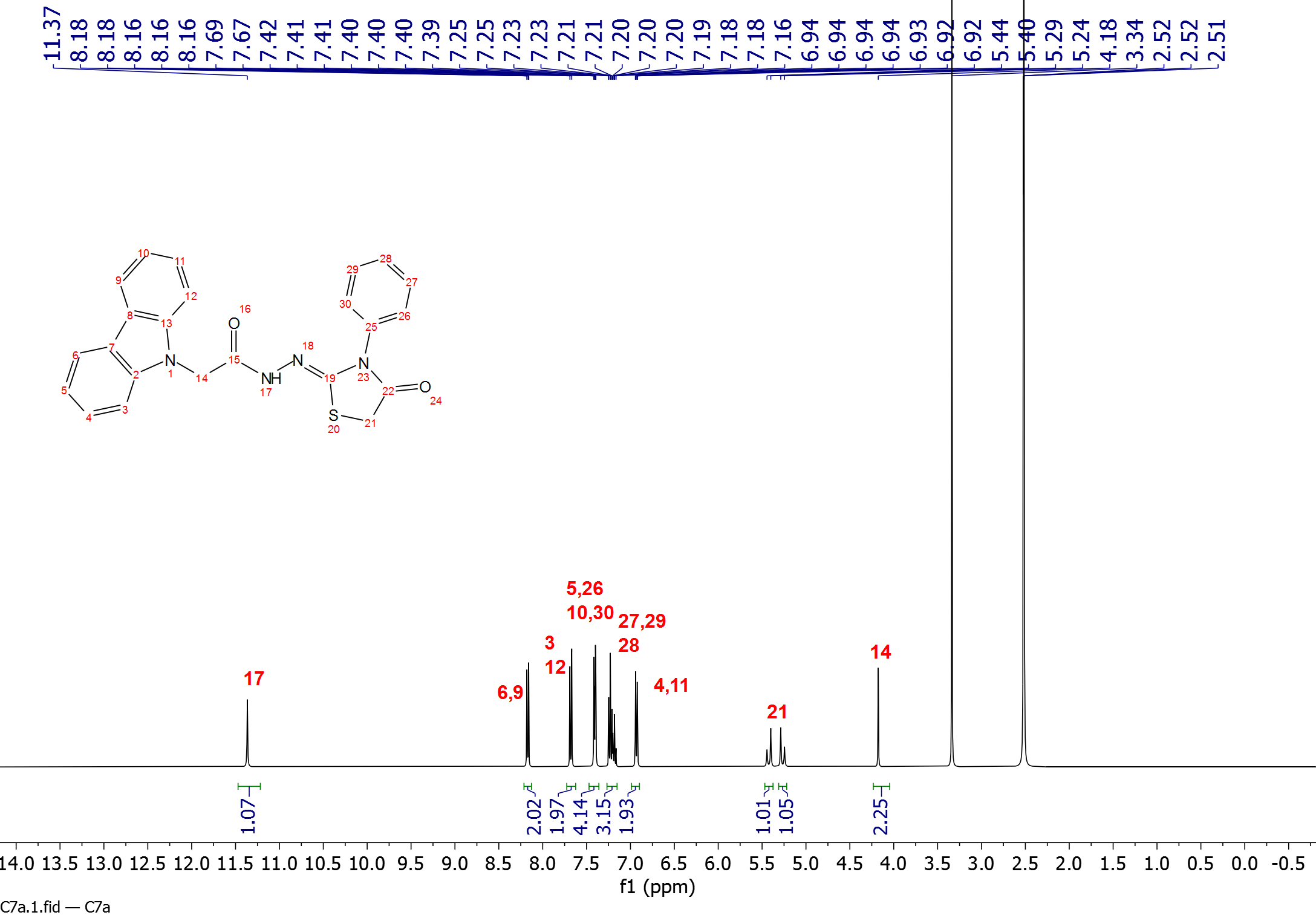
Figure S21. ^1^H-NMR Spectra of Compound **8**


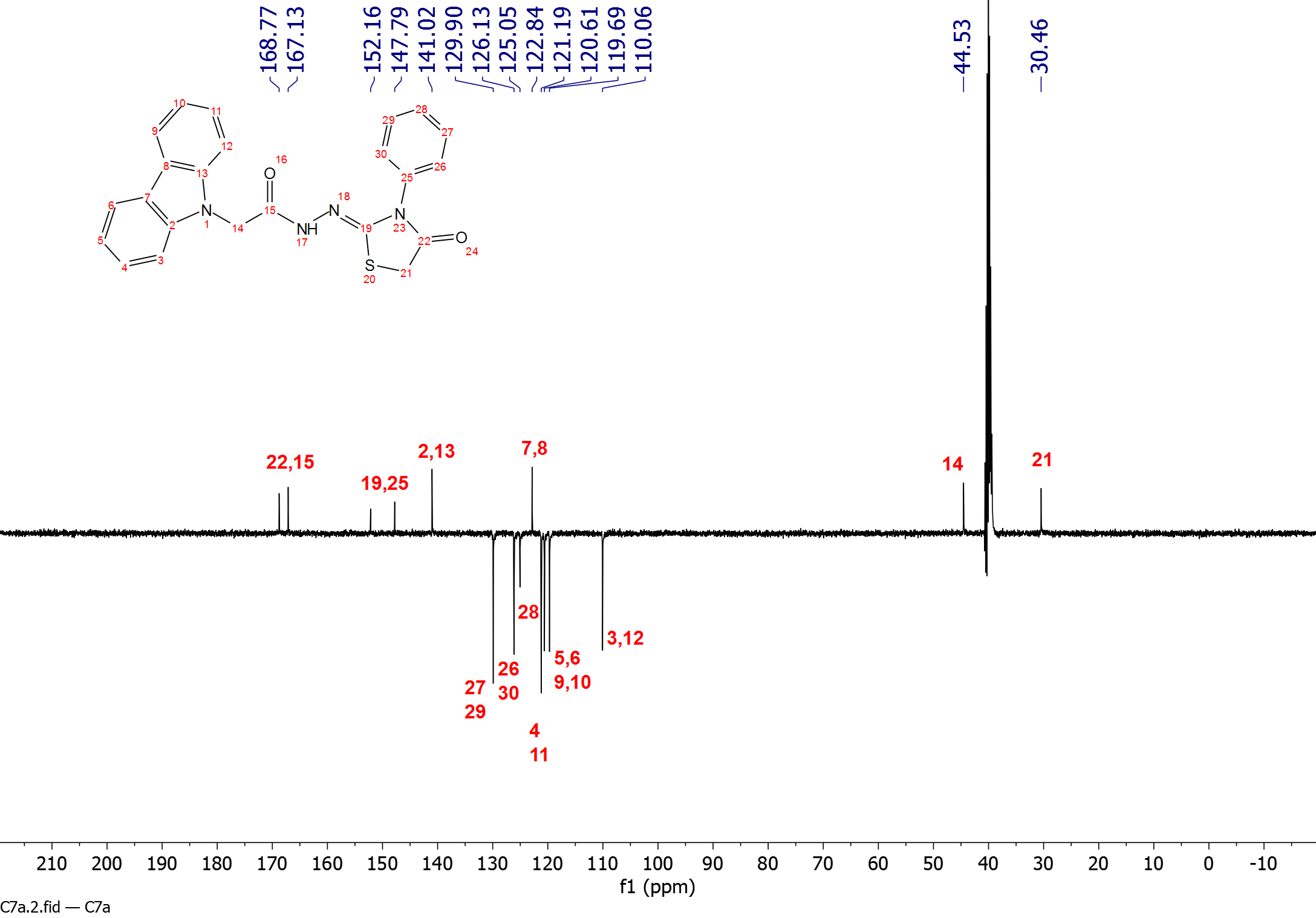


Figure S22. ^13^C_APT_-NMR Spectra of Compound **8**

# Spectrums of Compound 9


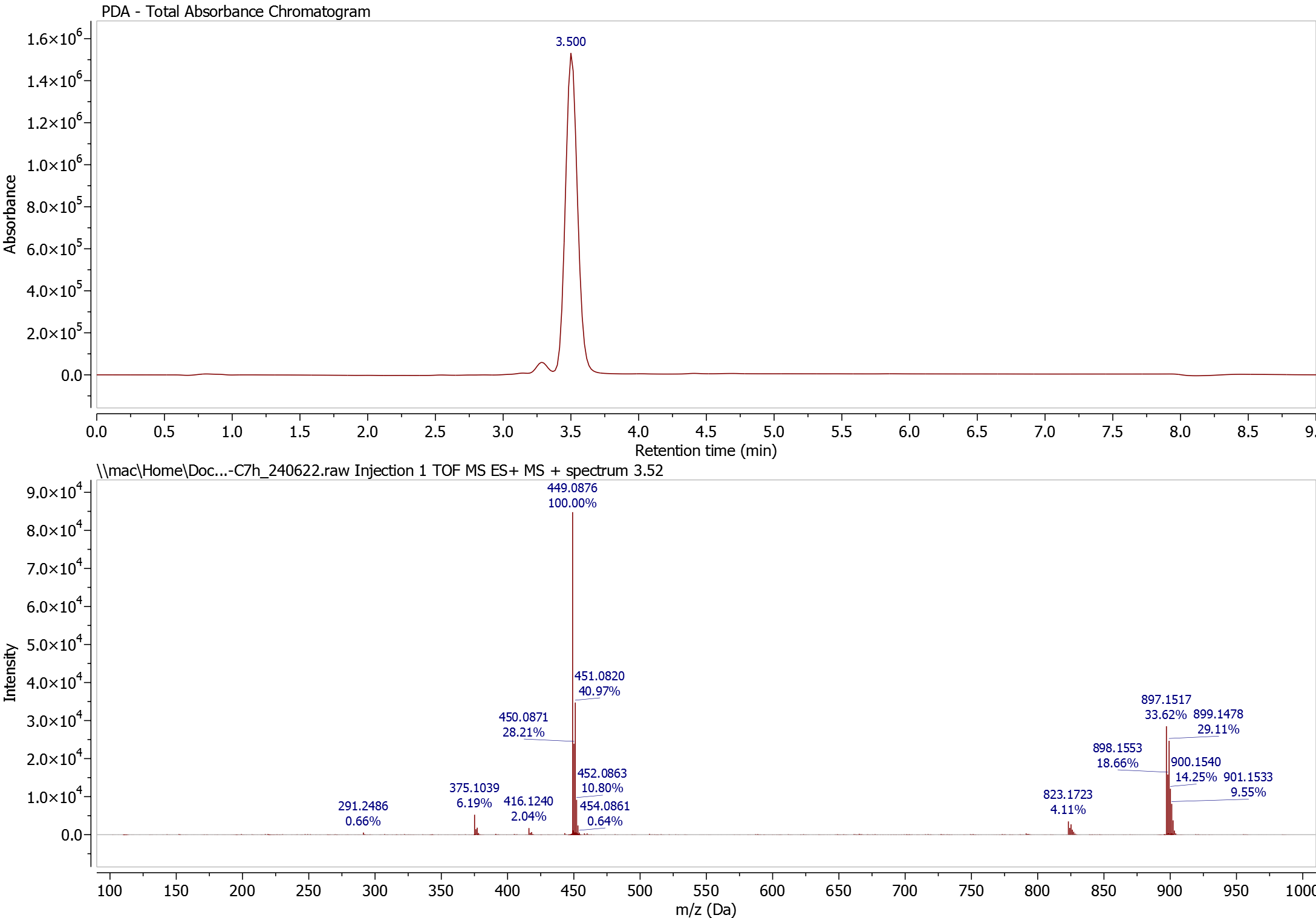


Figure S23. LC-MS Spectrum of Compound **9**


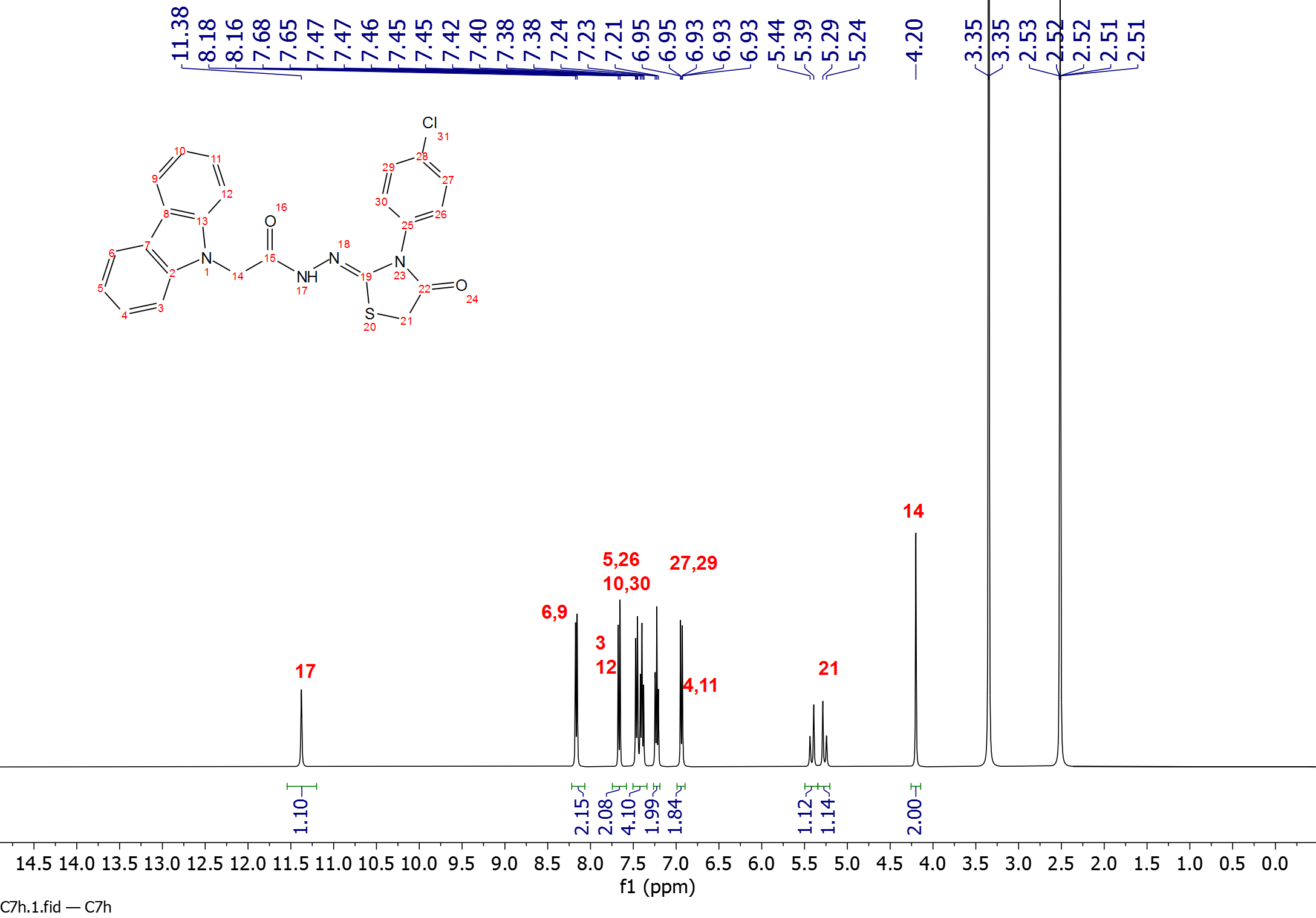
Figure S24. ^1^H-NMR Spectra of Compound **9**


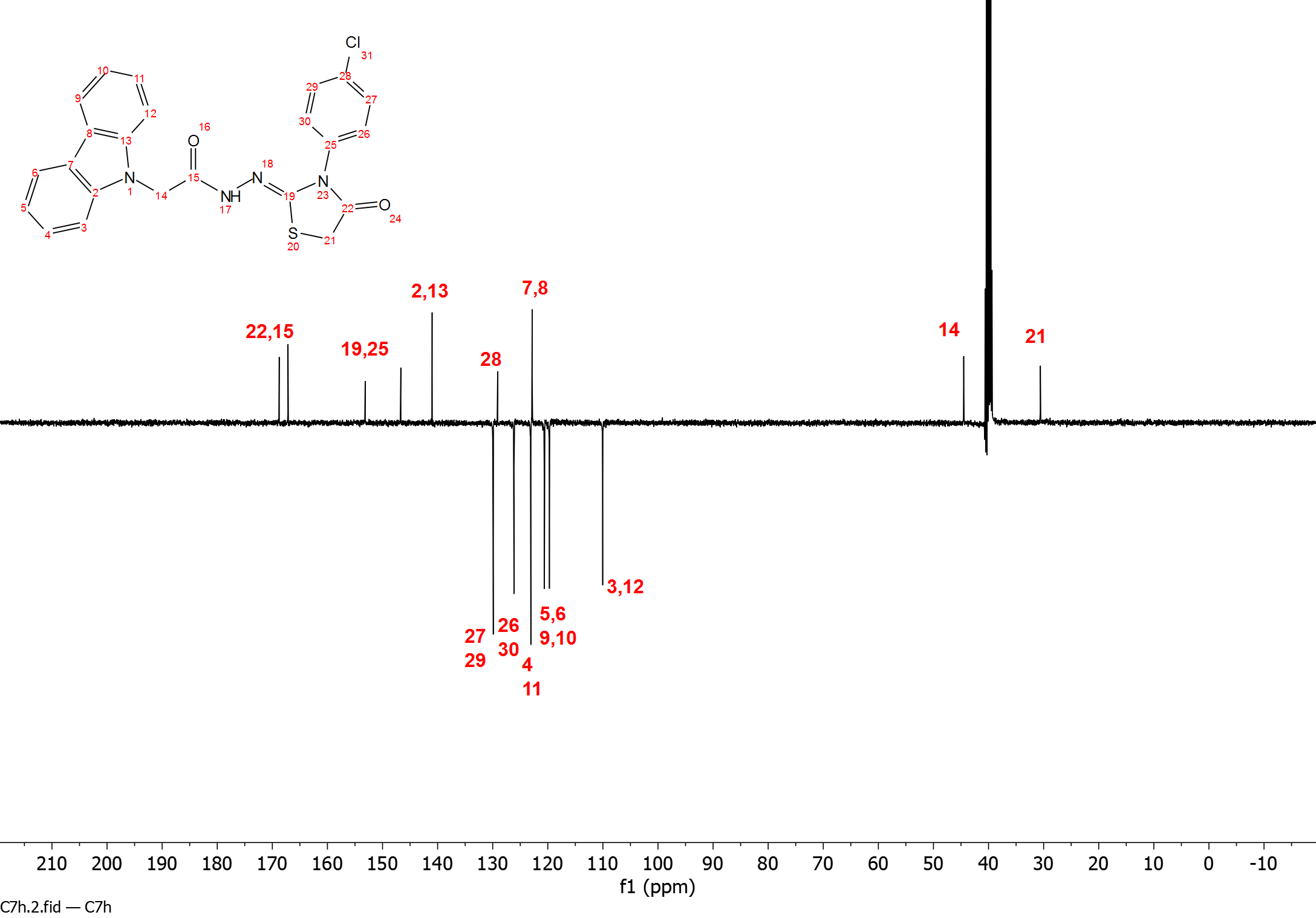


Figure S25. ^13^C_APT_-NMR Spectra of Compound **9**

# Spectrums of Compound 10


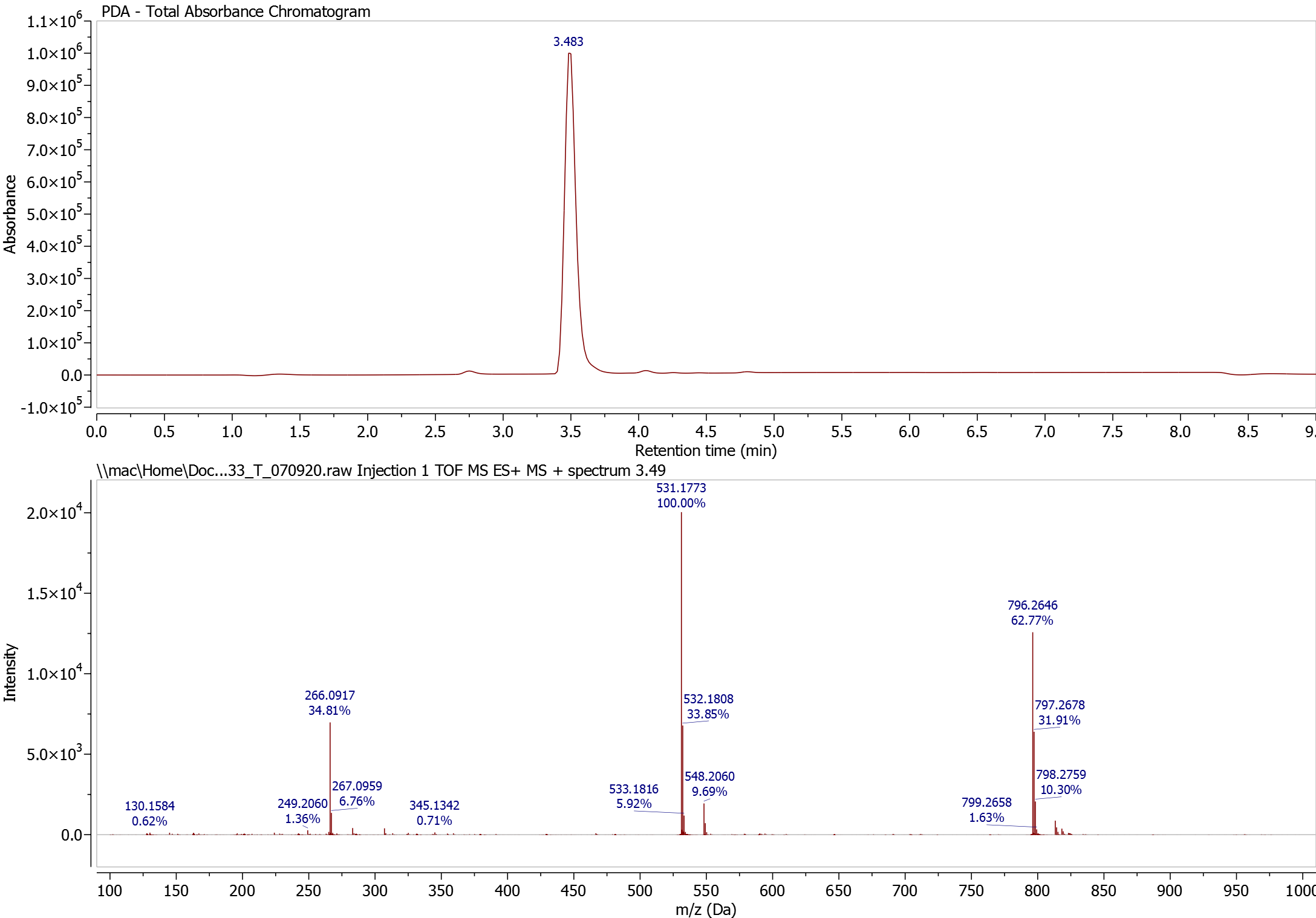


Figure S26. LC-MS Spectrum of Compound **10**


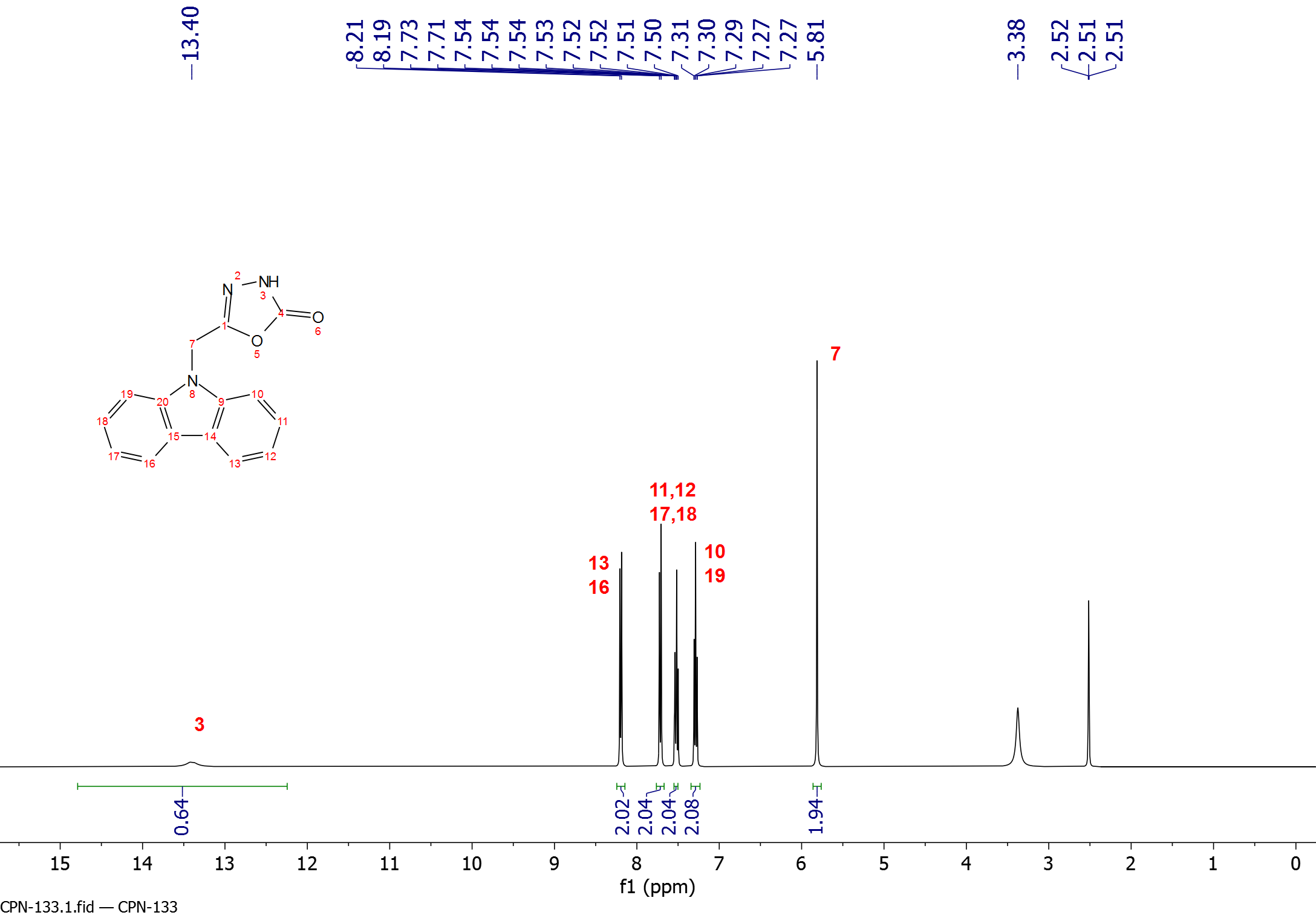

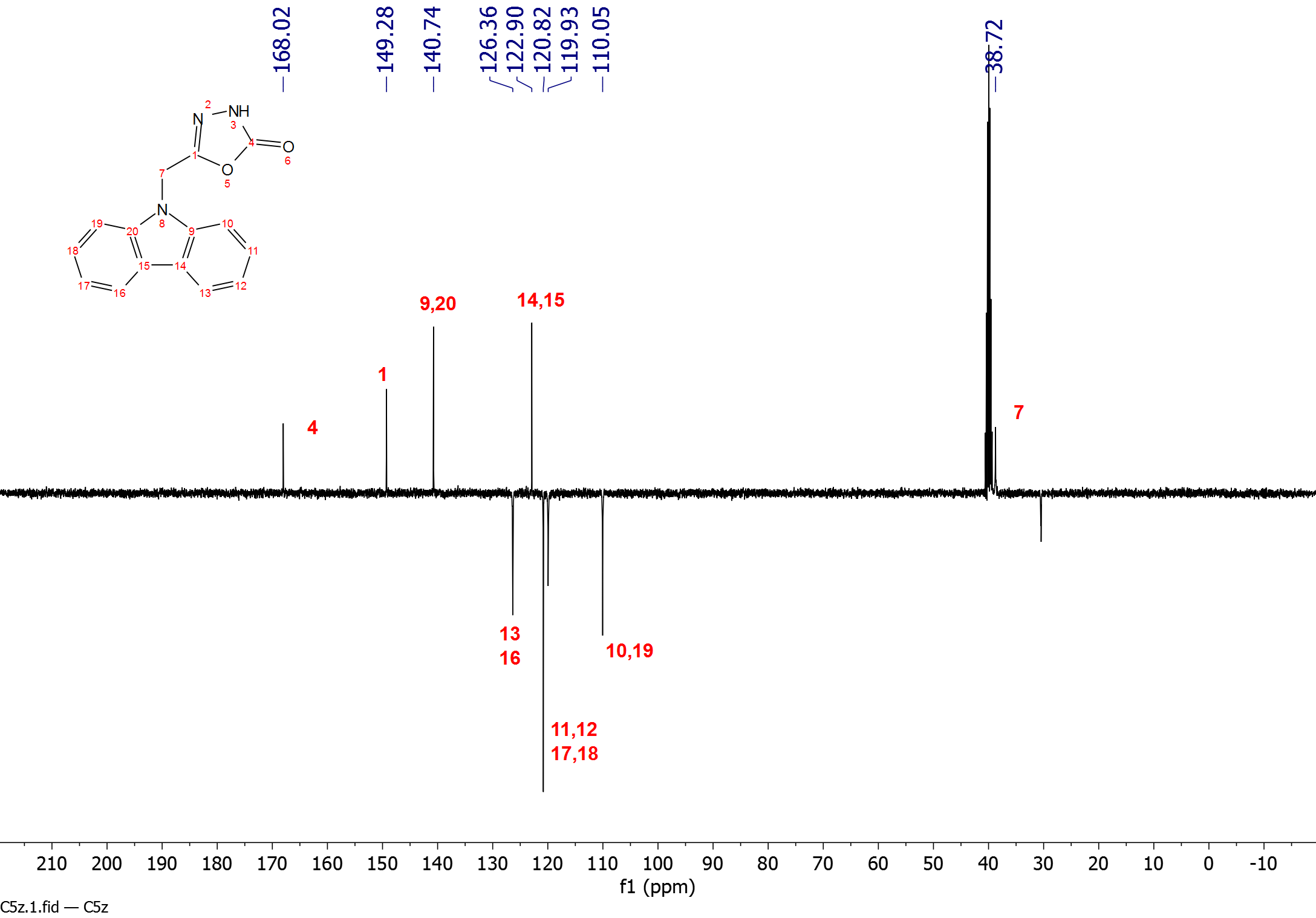
Figure S27. 1H-NMR Spectra of Compound **10**

Figure S28. ^13^C_APT_-NMR Spectra of Compound **10**

# Spectrums of Compound 11


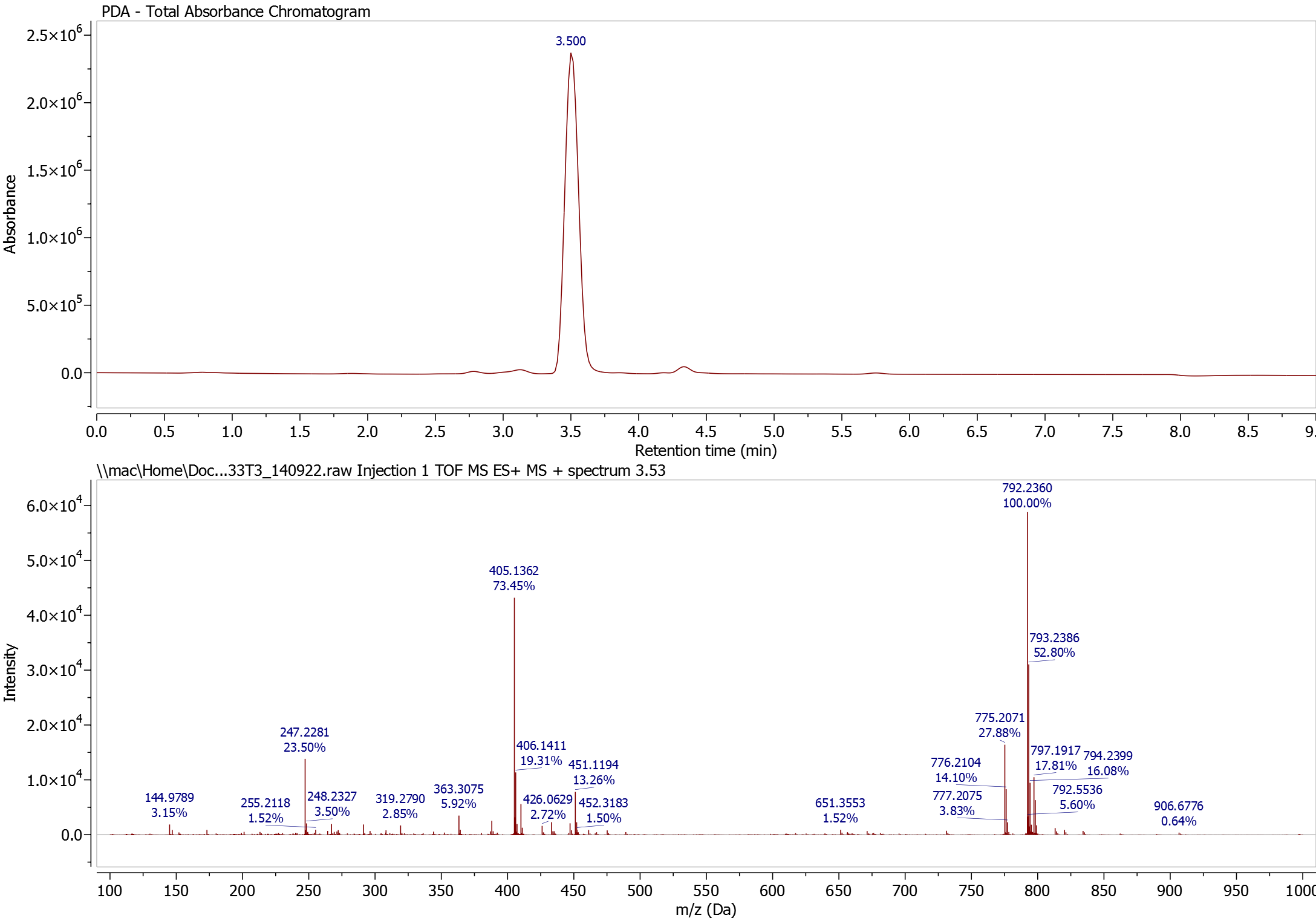


Figure S29. LC-MS Spectrum of Compound **11**


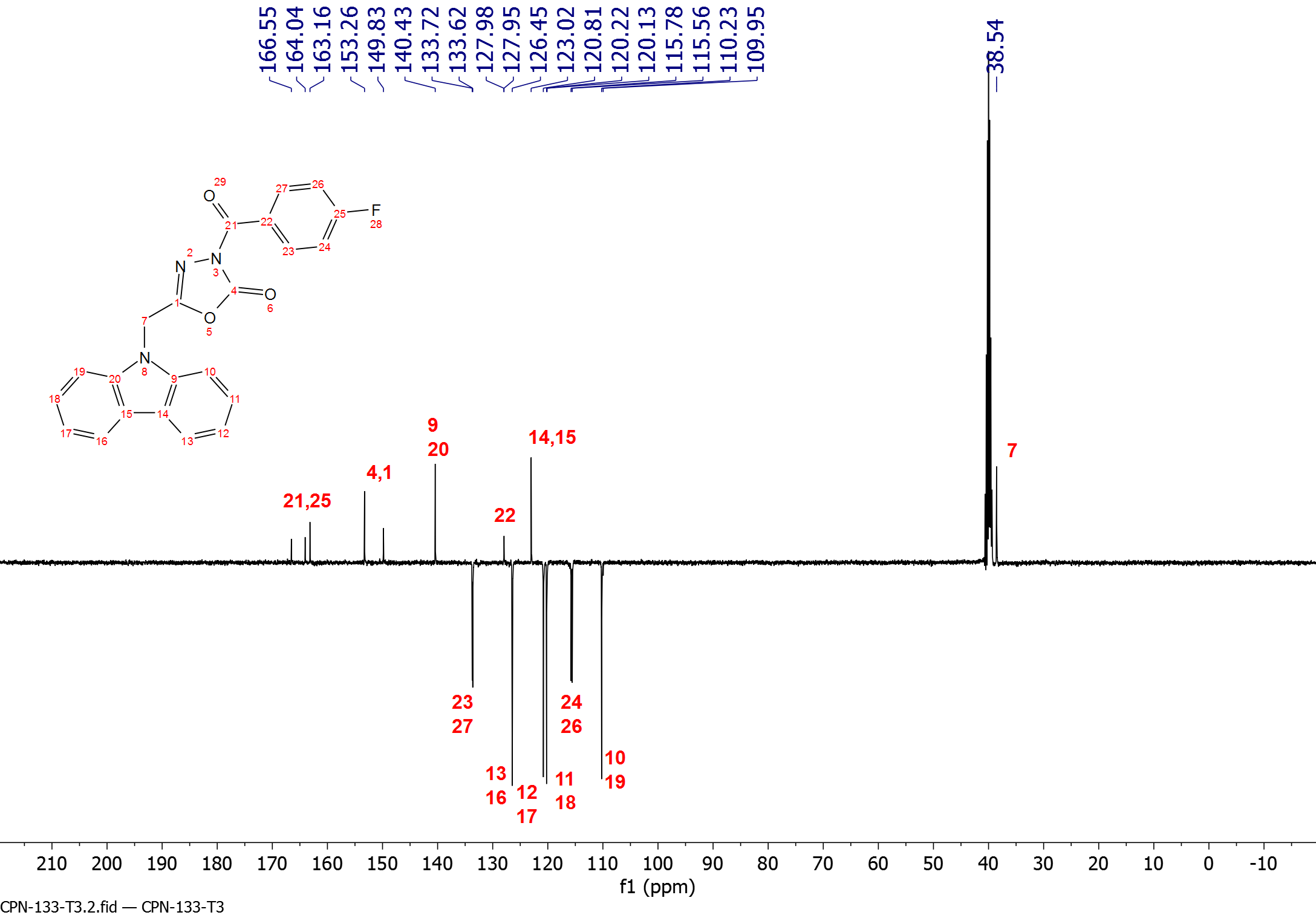

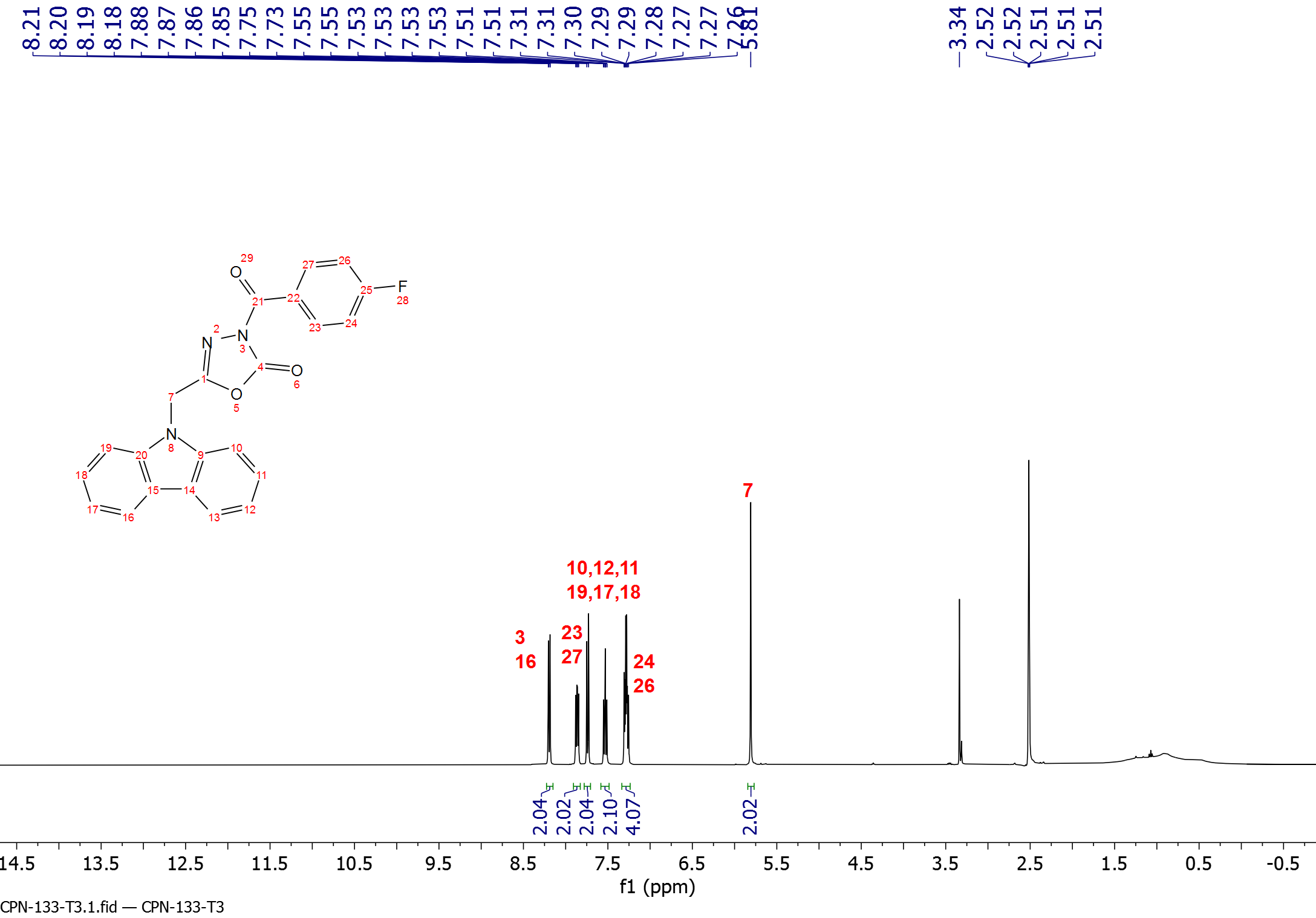
Figure S30. ^1^H-NMR Spectra of Compound **11**

Figure S31. ^13^C_APT_-NMR Spectra of Compound **11**

#
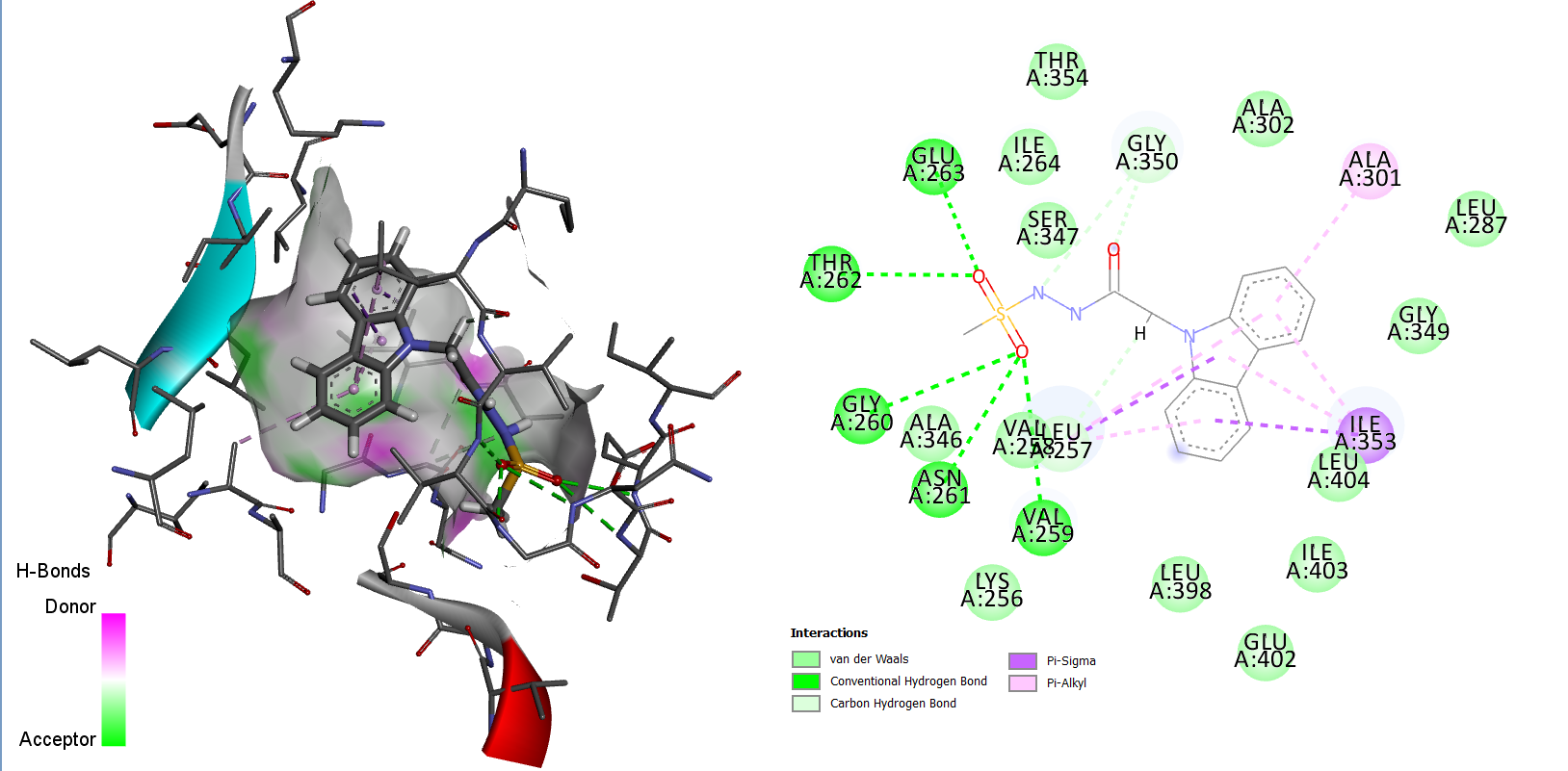
Docking Interactions of Compounds 2, 4, 5, 8, 9, 10, and 11

**Figure S32.** 3D and 2D forms of **2**+3UNI (A Chain).


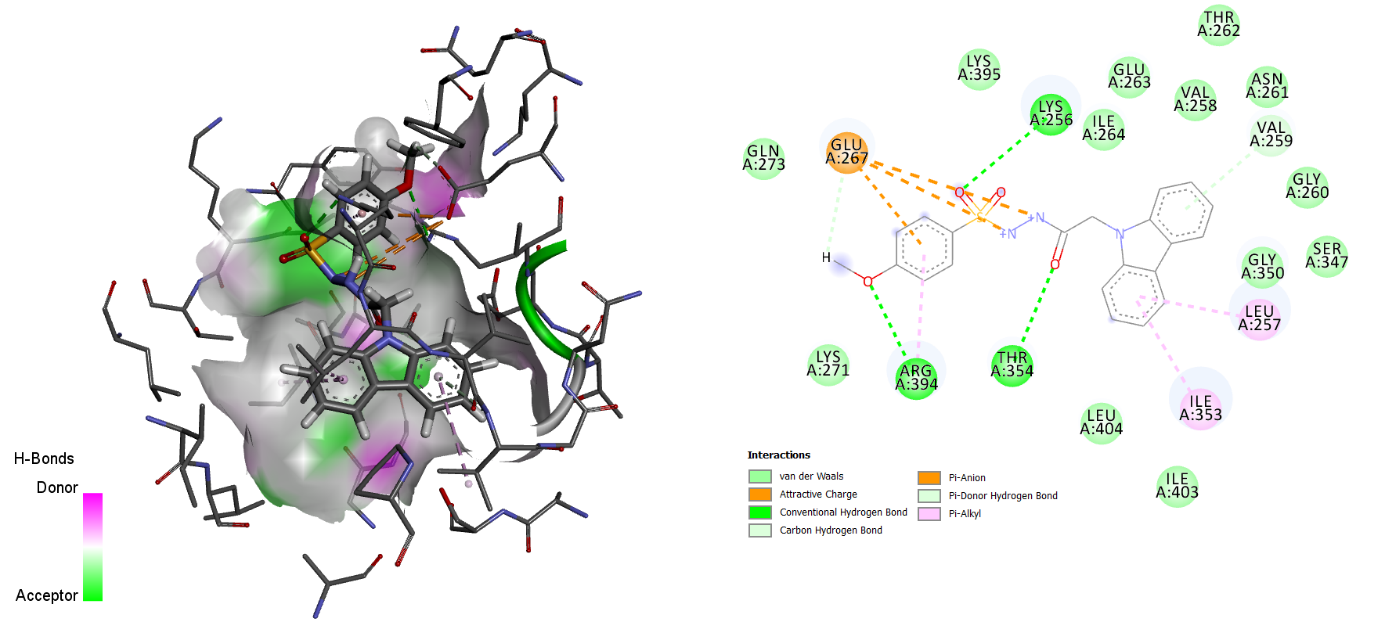


**Figure S33.** 3D and 2D forms of **4**+3UNI (A Chain).


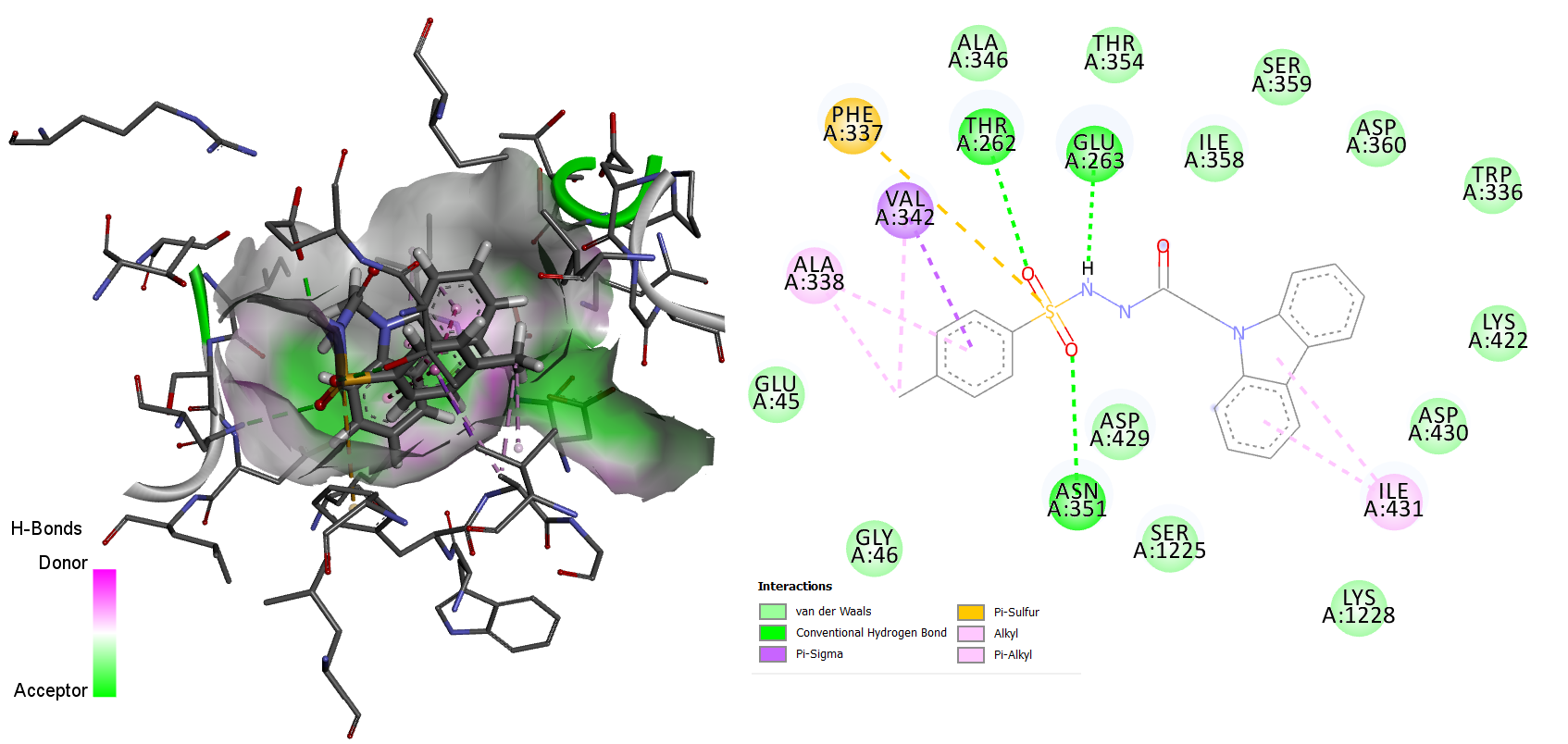


**Figure S34.** 3D and 2D forms of **5**+3UNI (A Chain).


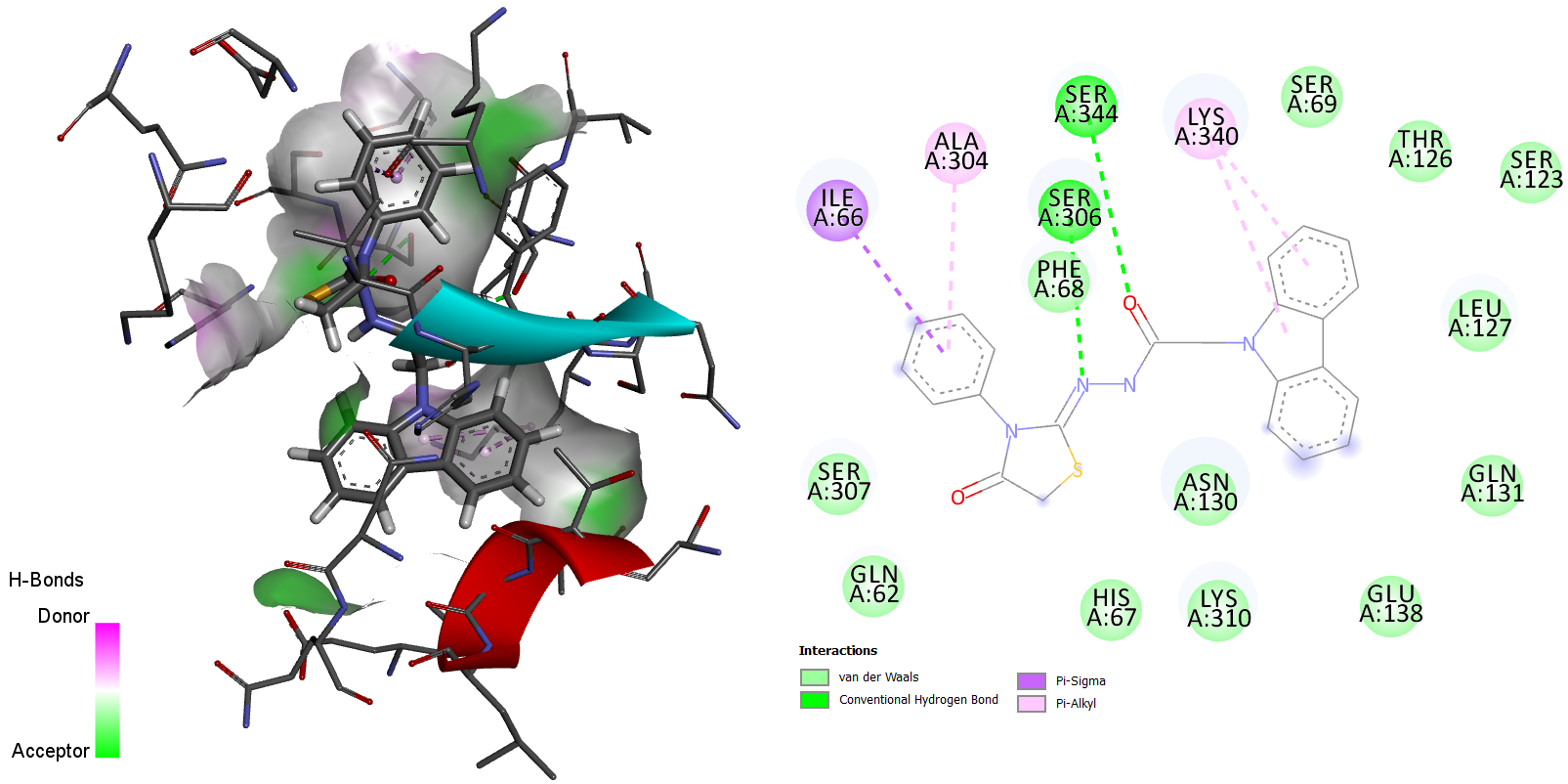


**Figure S35.** 3D and 2D forms of **8**+3UNI (A Chain).


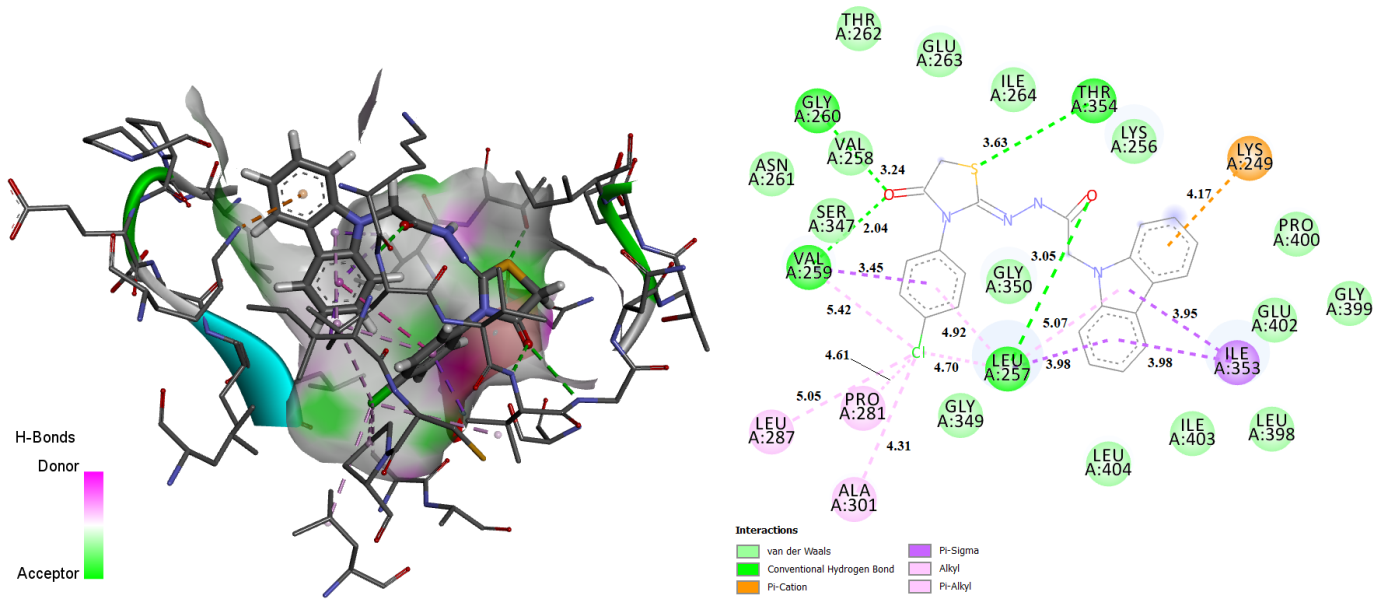


**Figure S36.** 3D and 2D forms of **9**+3UNI (A Chain).


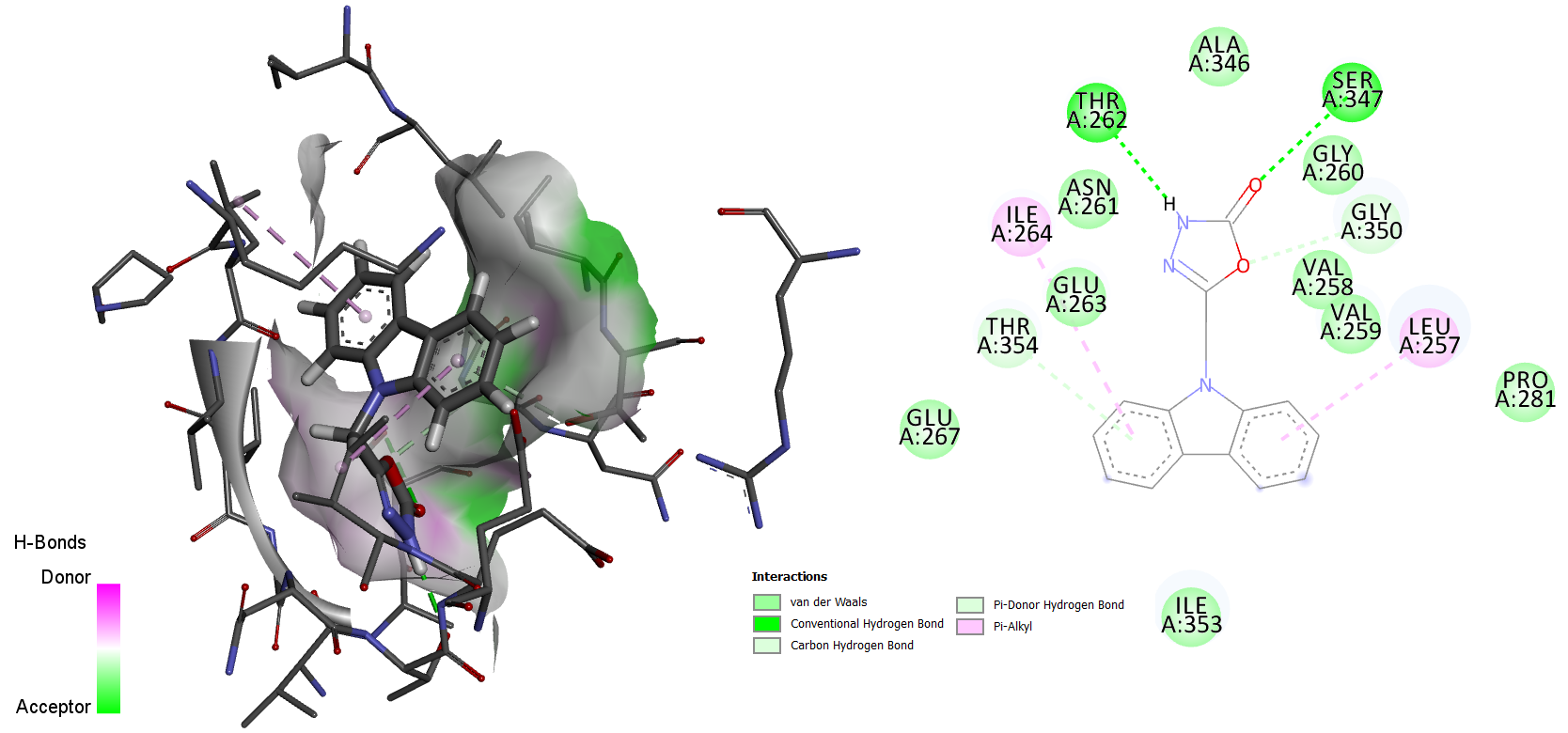


**Figure S37.** 3D and 2D forms of **10**+3UNI (A Chain).


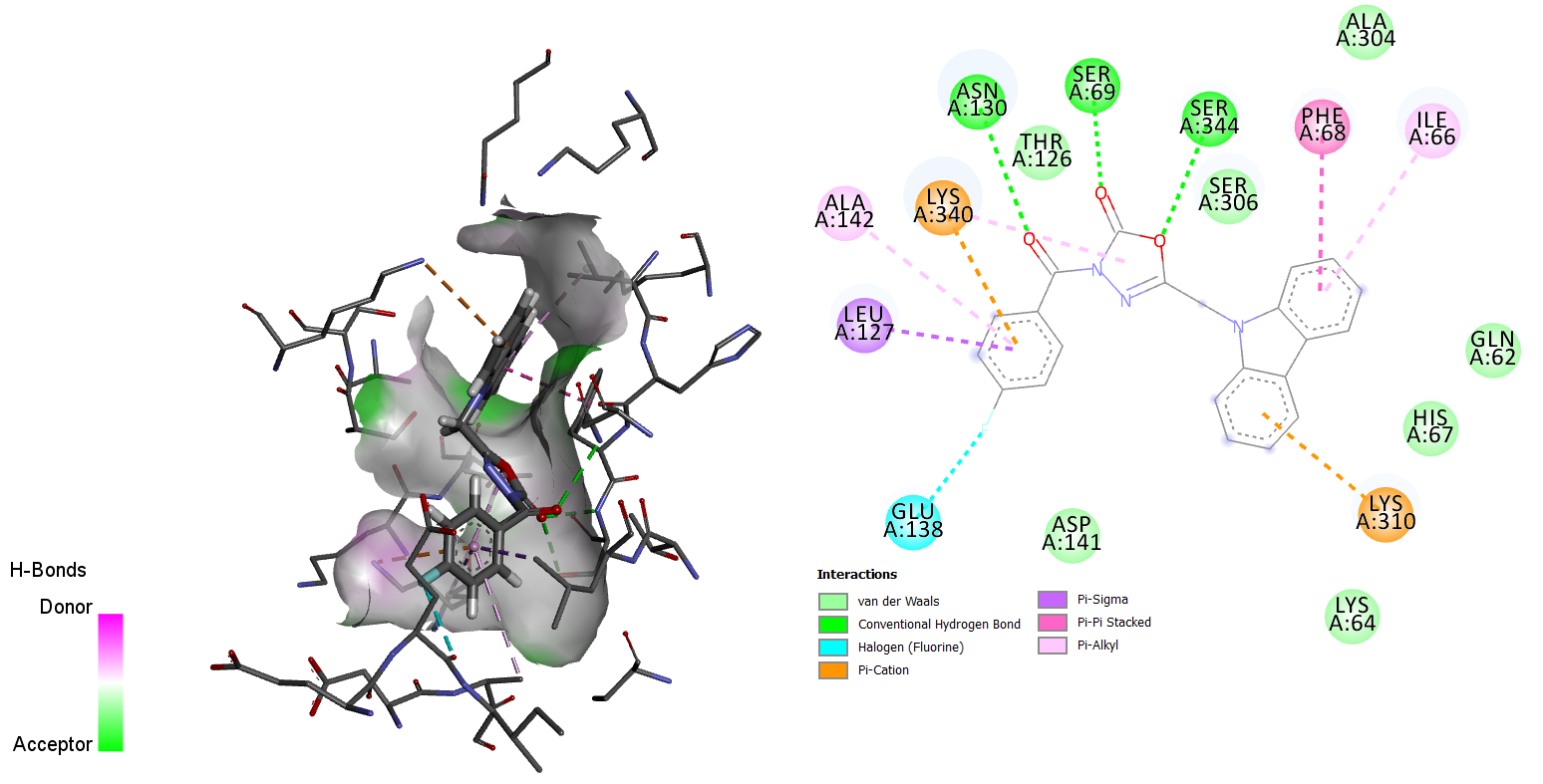


**Figure S38.** 3D and 2D forms of **11**+3UNI (A Chain).


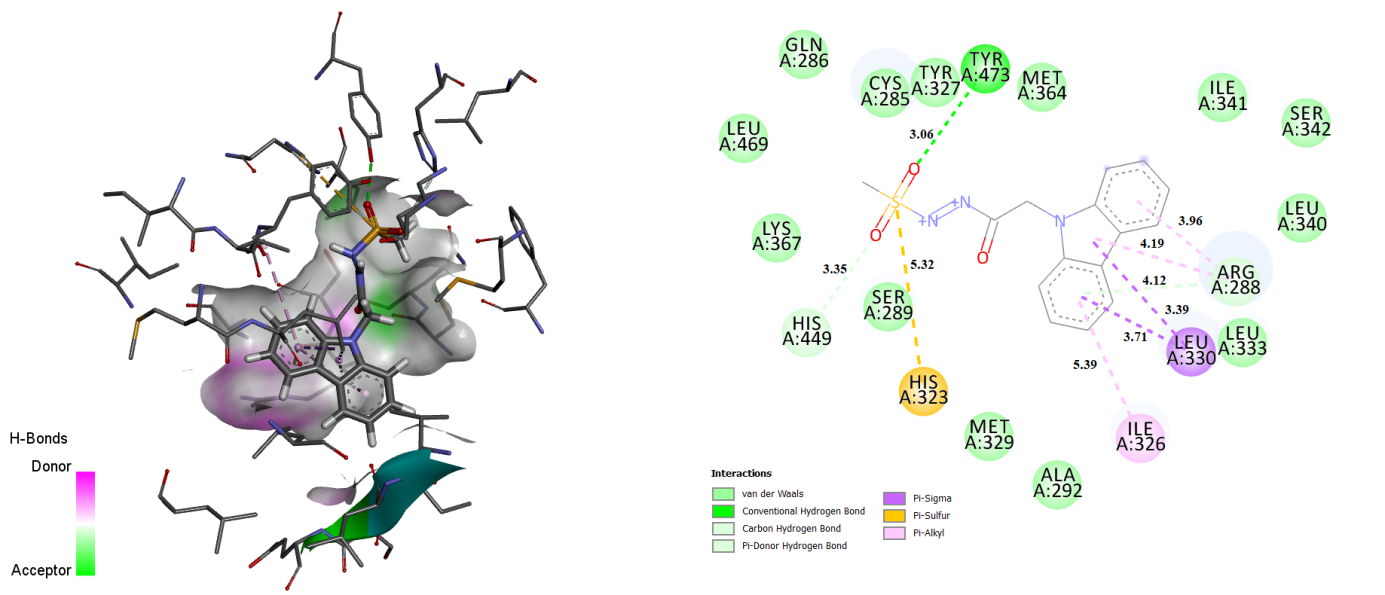


**Figure S39.** 3D and 2D forms of **2**+3VSO (A Chain).


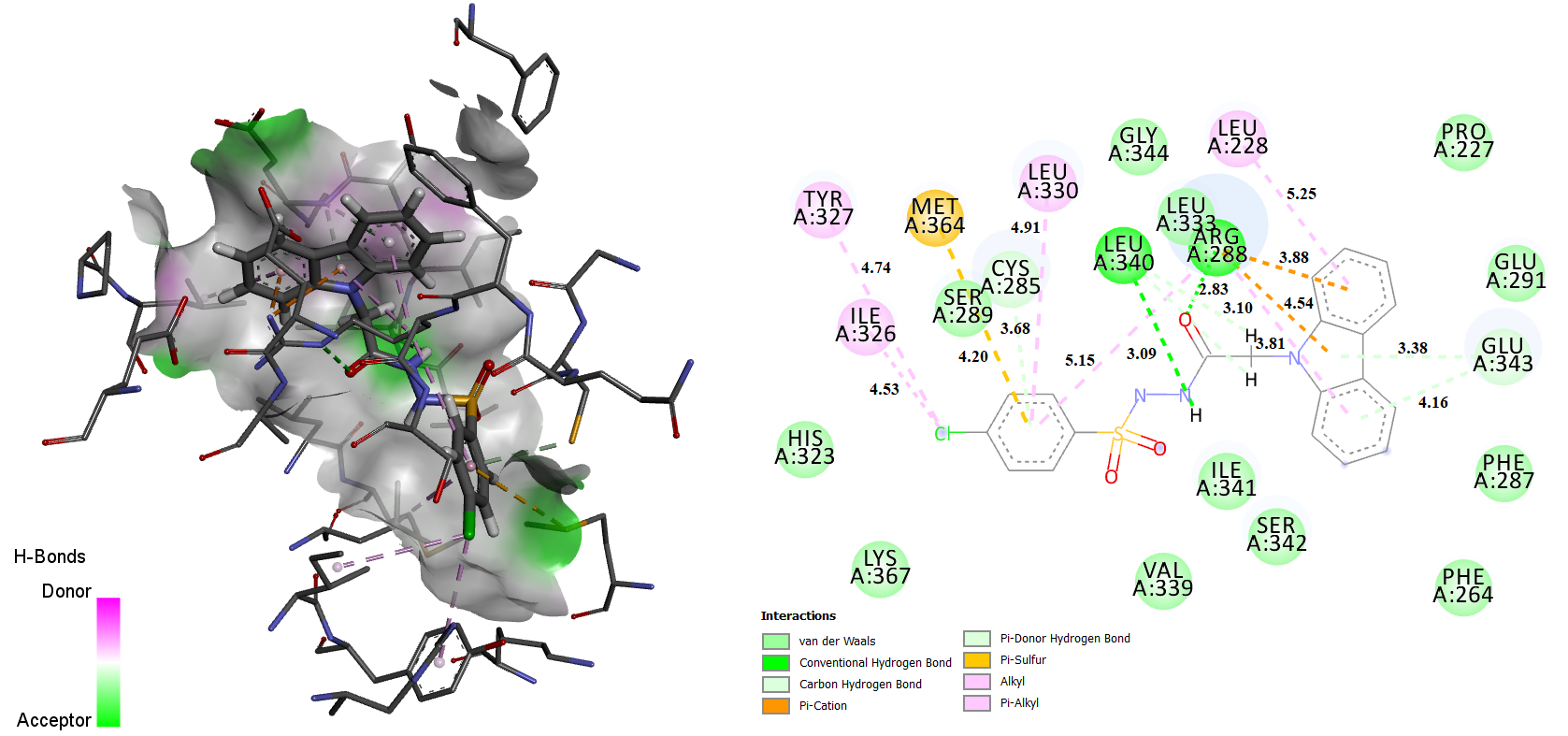


**Figure S40.** 3D and 2D forms of **3**+3VSO (A Chain).


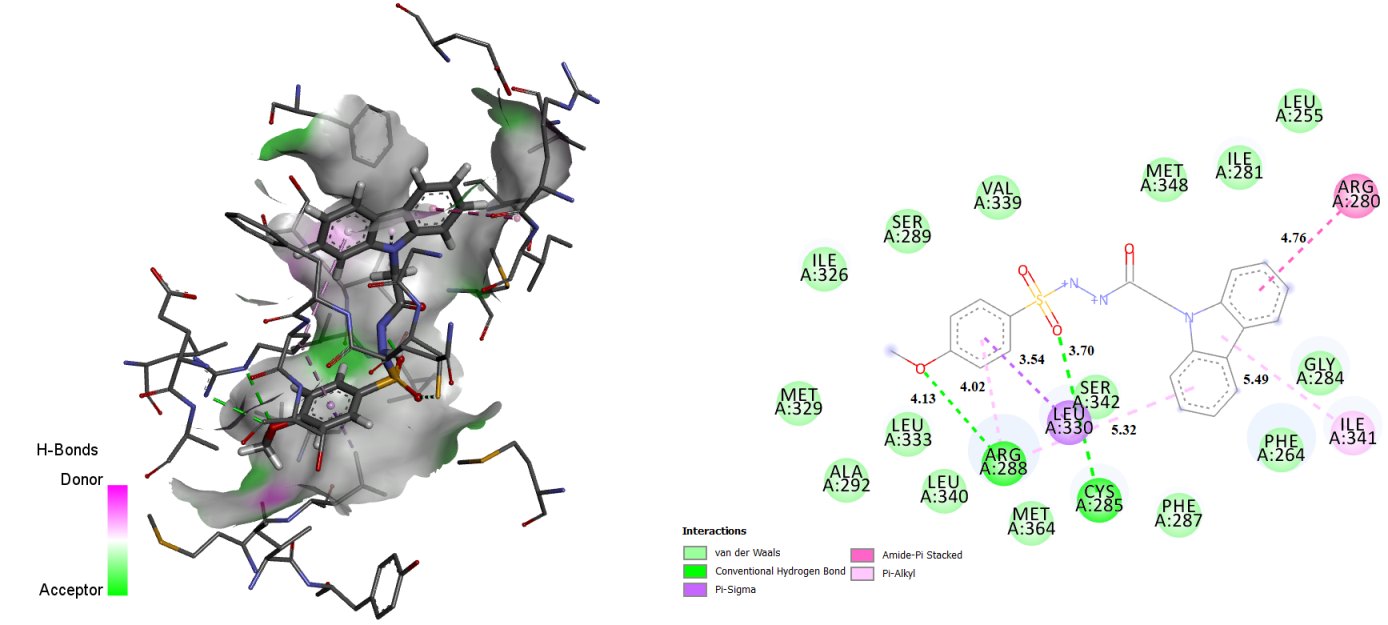


**Figure S41.** 3D and 2D forms of **4**+3VSO (A Chain).


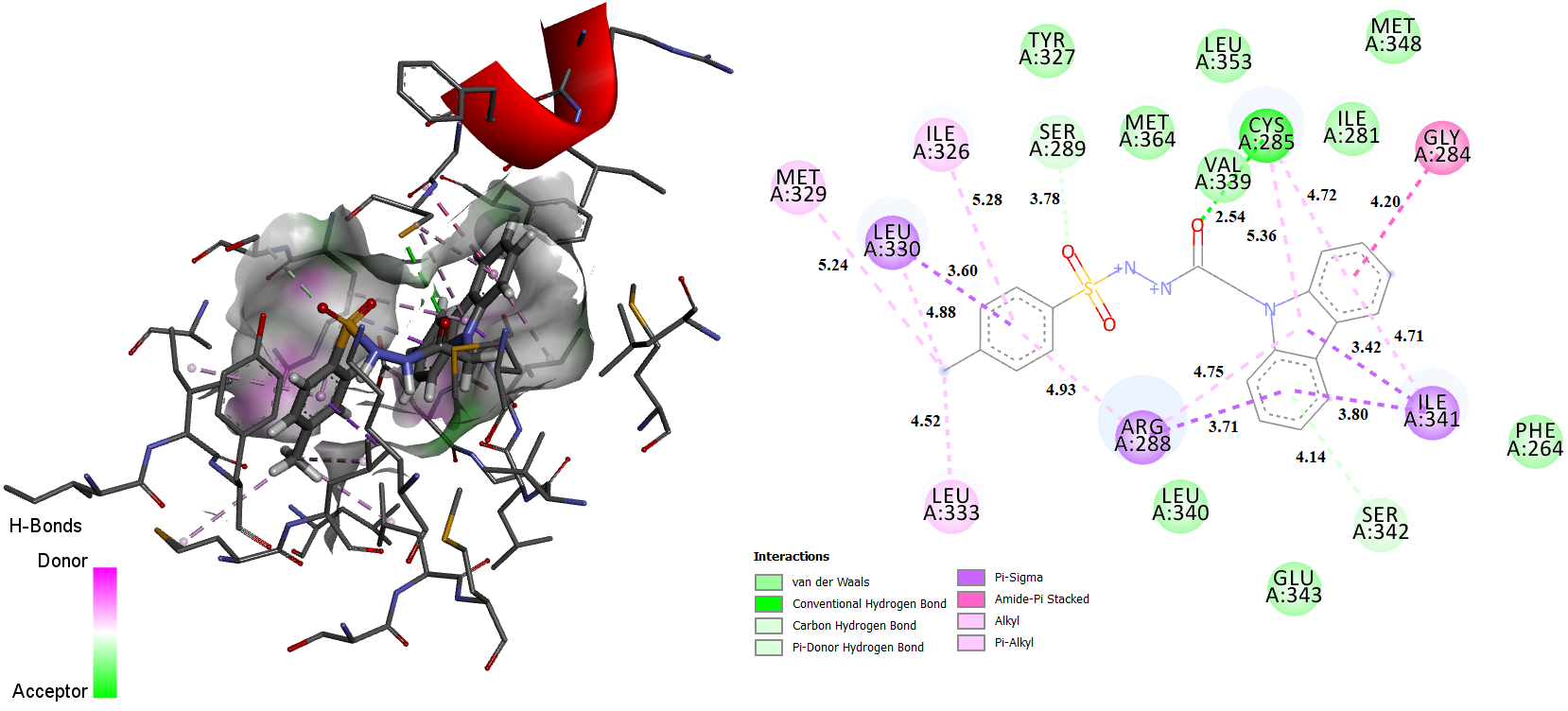


**Figure S42.** 3D and 2D forms of **5**+3VSO (A Chain).


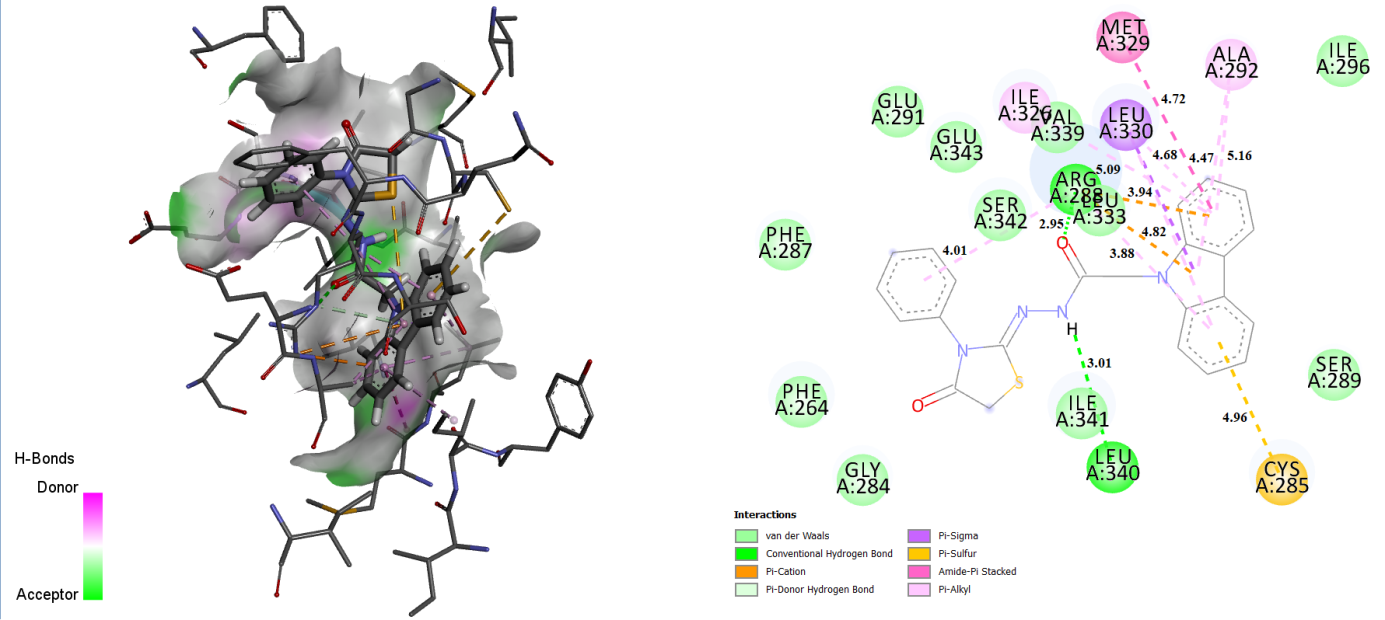


**Figure S43.** 3D and 2D forms of **8**+3VSO (A Chain).


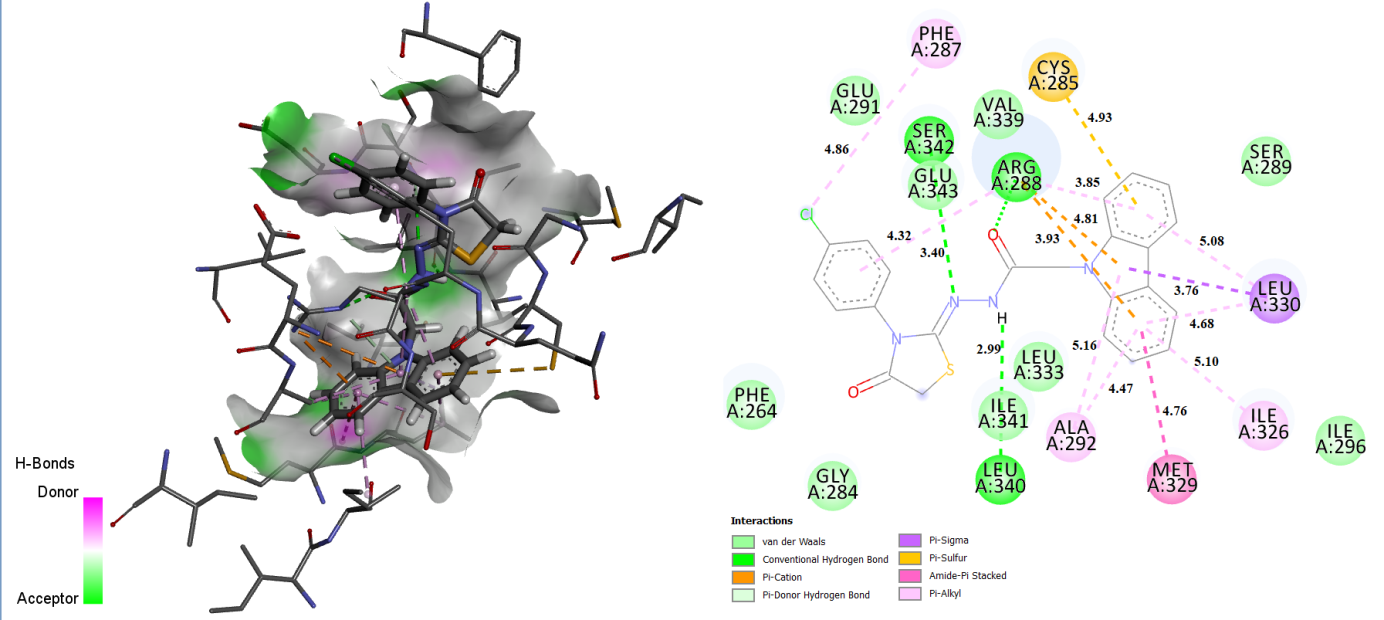


**Figure S44.** 3D and 2D forms of **9**+3VSO (A Chain).


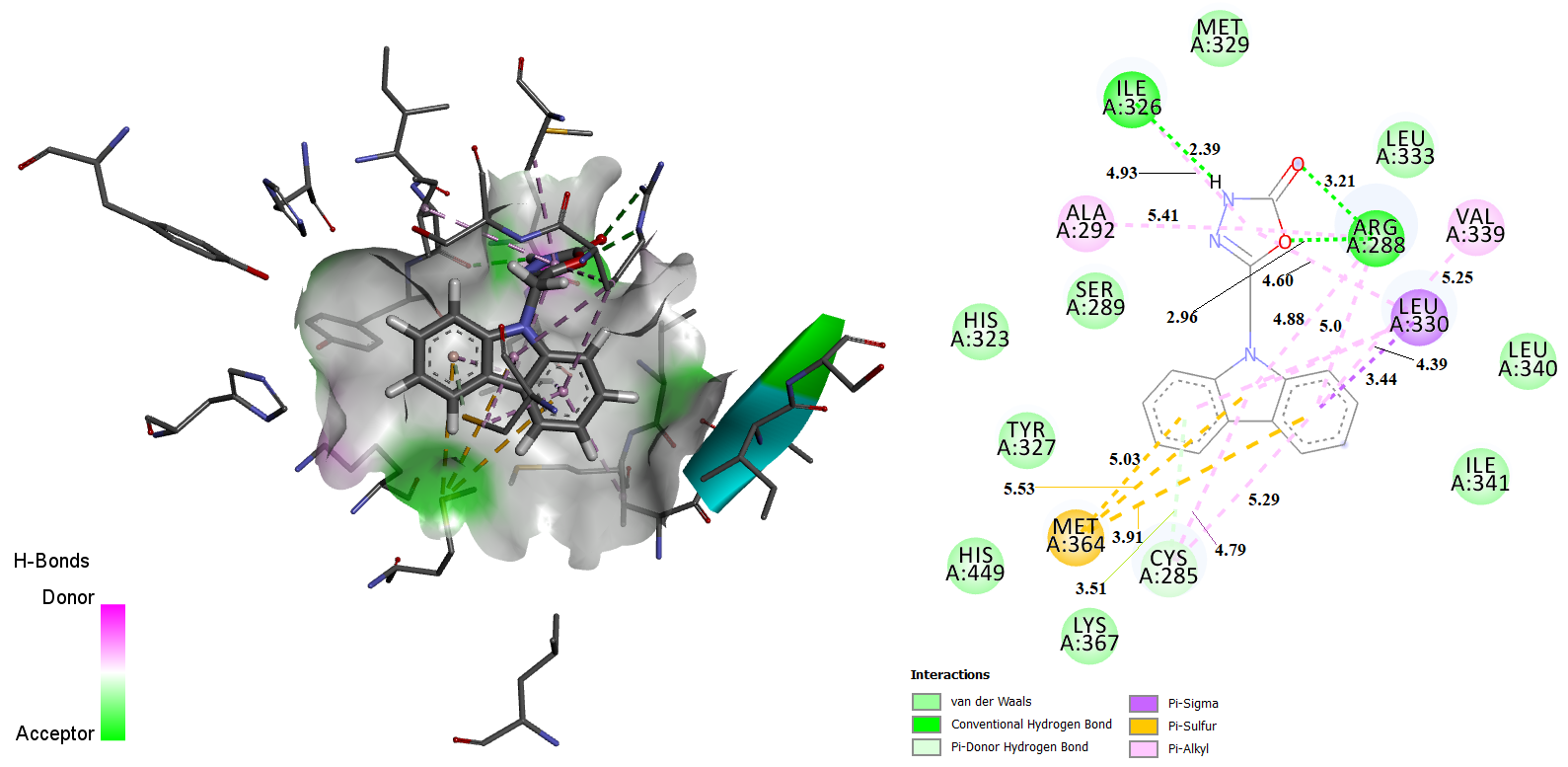


**Figure S45.** 3D and 2D forms of **10**+3VSO (A Chain).
